# Analysis of the global burden of cardiovascular diseases linked to exposure to ambient particulate matter pollution from 1990 to 2019

**DOI:** 10.3389/fpubh.2024.1391836

**Published:** 2024-10-02

**Authors:** Binbin Zou, Ping Wu, Juan Luo, Le Li, Ming Zhou

**Affiliations:** ^1^Department of Hematology, Hunan Provincial People's Hospital, The First Affiliated Hospital of Hunan Normal University, Changsha, China; ^2^Department of Pharmacy, Changde Hospital, Xiangya School of Medicine, Central South University, Hunan, China; ^3^Department of Cardiology, The Affiliated Hospital of Southwest Medical University, Luzhou, China

**Keywords:** ambient PM pollution, cardiovascular diseases, disability-adjusted life years, Global Burden of Disease, sociodemographic index

## Abstract

**Background:**

This research endeavors to scrutinize the temporal trends and global burden of cardiovascular diseases (CVDs) associated with ambient particulate matter (PM) pollution spanning from 1990 to 2019.

**Methods:**

Age-standardized death rates (ASDRs) and age-standardized disability-adjusted life years (DALYs) for CVDs, as well as their estimated annual percentage changes (EAPCs), were calculated using data from the Global Burden of Disease Study 2019 (GBD 2019).

**Results:**

The global ASDR and age-standardized DALYs due to CVDs associated with PM pollution increased from 1990 to 2019, with a higher increase in males. The burden was higher among middle-aged and older adults. The ASDR and DALYs increased in low-Socio-demographic Index (SDI), low–middle-SDI, and middle-SDI countries, while they decreased in high-SDI countries. The highest burden was observed in Central Asia, North Africa, the Middle East, East Asia, and South Asia. The highest burdens were reported in Iraq, Egypt, and Uzbekistan at the national level.

**Conclusion:**

The burden of CVDs linked to PM pollution has grown significantly from 1990 to 2019, with variations across regions and countries, highlighting the need for targeted prevention and pollution management strategies.

## 1 Introduction

Cardiovascular diseases (CVDs), encompassing atrial fibrillation, stroke, ischemic heart disease, heart failure, among others, are the leading causes of mortality and disability worldwide. Between 1990 and 2019, there was a global increase in the total number of CVD cases from 271 million to 523 million, with the corresponding rise in CVD-related deaths from 12.1 million to 18.6 million. Additionally, the disability-adjusted life years (DALYs) attributed to CVDs escalated from 17.7 million to 34.4 million person-years during this period (1). In 2019, China, India, Russia, the United States, and Indonesia were identified as the top five nations with the highest CVD fatalities. Meanwhile, Uzbekistan, the Solomon Islands, and Tajikistan exhibited the highest age-standardized death rates (ASDR) for CVDs, while France and Japan had the lowest ASDRs (2). According to the China Cardiovascular Health and Disease Report 2020, there were an estimated 330 million CVD patients in China in 2020.

Numerous risk factors contribute to the incidence of Cardiovascular Diseases (CVDs), with the eight most significant contributors to the global burden of CVD being high systolic blood pressure, unhealthy diet, elevated levels of low-density lipoprotein cholesterol, ambient particulate matter (PM) pollution, smoking, high fasting blood sugar levels, a high body-mass index, and renal dysfunction (2, 3). A substantial body of evidence indicates that ambient PM pollution is a major environmental risk factor for both the incidence and mortality associated with CVD (2, 4–6). This type of pollution comprises solid or liquid substances suspended in the air, including chemical species such as minerals or organic compounds like polycyclic aromatic hydrocarbons, and biological species such as pollen, fungi, and bacteria (7, 8). These chemical substances primarily originate from automobile exhaust and industrial emissions, including those from power plants and garbage incinerators (9). PM is classified based on aerodynamic diameter into three main types: coarse PM (PM10-2.5), fine PM (PM2.5), and ultrafine PM (PM0.1) (10). The Global Burden of Disease Study (GBD) 2019 reported that worldwide exposure to PM over a long period resulted in approximately 4.2 million deaths in 2015, with the DALYs attributed to CVDs amounting to 103 million (11). In 2016, environmental PM was ranked as the sixth leading contributor to CVDs, whereas it had been seventh in 1990. A positive correlation has been observed between long-term exposure to PM_2.5_ and high systolic blood pressure, which is a known risk factor for CVD (12). Furthermore, exposure to elevated concentrations of PM_2.5_ is associated with an increased risk of CVD mortality, with a hazard ratio of 1.19 (95% confidence interval [CI]: 1.10, 1.28) (13). The study also found a significant correlation between the concentrations of nitrogen dioxide and nitrogen oxides in the environment and both the incidence and mortality of CVD (13). Assessments conducted within the GBDs have suggested that environmental air pollution exerts a more substantial impact on mortality than other major modifiable risk factors such as low physical activity and a high sodium diet. Moreover, adherence to air-quality standards for PM_2.5_ could potentially prevent premature deaths from CVDs (14).

Only a handful of studies have quantified and juxtaposed the burden of CVDs associated with exposure to ambient PM pollution at a global scale. However, comprehending the spatial distribution and temporal progression of CVD burdens related to ambient PM pollution is essential for the prevention of CVDs and their associated conditions, as well as for air pollution management. To this end, we utilized data from the GBD 2019 database to estimate both the burden and mortality rates of CVDs associated with exposure to ambient PM pollution across various regions and countries worldwide in 2019, as well as their temporal trends from 1990 to 2019.

## 2 Methods

### 2.1 Data sources

All data were obtained from the GBD 2019 database, which contains highly reliable data based on the assessment of 204 global environmental, occupational, and metabolic risk factors, 87 hazardous behaviors, and 369 diseases and injuries (15). Mortality and illness burden data for different countries and regions worldwide from 1990 to 2019 were extracted from the GBD 2019 database. These data included numbers of deaths, numbers of DALYs, ASDRs, and age-standardized DALY rates due to CVDs associated with exposure to ambient PM pollution, as well as Socio-demographic Index (SDI) data. The SDI is a new composite indicator developed by the GBD 2019 that is based on *per capita* income, average years of education, and total fertility rate. According to their SDI values, 204 countries and regions can be classified into five areas: low-SDI (<0.46), low–middle-SDI (0.46–0.60), middle-SDI (0.61–0.69), high–middle-SDI (0.70–0.81), and high-SDI (>0.81) areas.

In the International Classification of Diseases (ICD-10) revision 10, CVD codes are denoted as I00–I99. The GBD 2019 employed a counterfactual analysis approach based on comparative risk assessment (CRA). This method assumes that exposure levels to other risk factors remain constant while comparing the acute exposure distribution of a target population to PM pollution with the theoretical minimum risk exposure level (TMREL). Subsequently, it computes the proportion of the total CVD burden attributable to ambient PM pollution within the target population, thereby enabling the calculation of the Population Attributable Fraction (PAF). We selected mortality and DALYs, multiplied by CVD-specific mortality and DALYs using PAF, to determine the mortality and DALYs associated with CVDs due to exposure to ambient PM pollution. The result was then divided by the respective country or region’s population to calculate the mortality rate and DALY rate for CVDs linked to exposure to ambient PM pollution.

We used the following general formula for consecutive risks to calculate the population linked score (PAF) by age, gender, location, and year for each risk factor *j*:


$$PAF_{\mathrm{joasgt}}=\frac{\int_{x=l}^{u}RR_{\mathrm{joasg}}\left(x\right)P_{\mathrm{jasgt}}\left(x\right)dx-RR_{\mathrm{joasg}}\left(\mathrm{TMRE}L_{jas}\right)}{\int_{x=l}^{u}RR_{\mathrm{joasg}}\left(x\right)P_{\mathrm{jasgt}}\left(x\right)dx}$$

where PAF*_joasgt_* is the PAF for *o* in age group *a*, gender *s*, location *g*, in year *t*; and RR*_joasg_ (x)* is the relative risk function of risk factor *j* at exposure level *x*. Confounding causes o were controlled as the lowest observed exposure level in age group *a*, gender *s*, and location g is *l*, and the highest is *u*. P_jasgt_ *(x)* represents the exposure distribution of age group a, gender s, location g, in year t at point x; and TMREL*_jas_* are TMRELs associated with risk causes *j*, age group a, and genders. When the risk exposure is classified into two or more categories, the formula is simplified to a discrete form. The estimation of PAF takes into account the risk function and individual exposure distribution for each age, gender, location, and year. PM data from 1990 to 2019 were obtained from the GBD 2019 database. Different levels of PM were considered, with specific focus on PM2.5. The calculation formula and principle of RR values involved in PAF in this study have been described in detail in previous study (16). Therefore, by sampling 1,000 from the risk function; generating 1,000 exposure distributions for each age, sex, location and year; and then sampling 1,000 from TMRELs, we propagated all sources of uncertainty into the PAF distribution. This allowed us to evaluate the attribution burden of PAF and risk factor combinations.

### 2.2 Statistical analysis

The results were stratified by gender, age, and SDI level. Mortality rates, DALYs, ASDRs, age-standardized DALYs, and estimated annual percentage changes (EAPCs) were calculated to assess the variations and trends in mortality rates and disease burdens (EAPCs) of CVD associated with exposure to ambient PM pollution among the global population from 1990 to 2019. EAPCs were fitted using the age-standardized rate (ASR) on the regression line, i.e., ln (ASR) = *α* + *β* x + Å， where x = year, and EAPC = 100 × (exp(β) − 1) (17). Pearson correlation analysis was employed to investigate the relationship between SDIs and ASDRs, age-standardized DALYs, and EAPCs. Locally estimated scatterplot smoothing, a locally weighted regression smoothing method, was utilized to fit the ASDR, age-standardized DALYs, and EAPC curves for SDIs. Case data and corresponding cases (per 10,000 people) were presented with 95% confidence intervals (CIs), and a bilateral *p* value of less than 0.05 was considered statistically significant. All analyses were performed using R core team (version 3.5.2). All data were sourced from the publicly available GBD database [Global Health Data Exchange (GHDx)]^1^.

## 3 Results

### 3.1 Global CVD burden linked to exposure to ambient PM pollution

In 1990, the global number of CVD deaths attributable to exposure to ambient PM pollution was 1,115, 609.56, with an ASDR of 30.75 (22.33, 39.66) and a gender ratio of 1.54. The corresponding number of DALYs was 27,446,835.53 (19,940,175.10, 36,011,833.88), the corresponding age-standardized DALYs was 681.83 (494.72, 888.87), and their gender ratio was 1.71. By 2019, these figures had risen to 2,475,395.02 (2,051,050.10, 2,871,325.06) for CVD deaths attributable to exposure to ambient PM pollution, with an ASDR of 30.85 (25.52, 35.78) and a gender ratio of 1.71. The corresponding number of DALYs was 60,910,841.13 (50,072,396.80, 70,319,155.87), the corresponding age-standardized DALYs was 737.21 (606.86, 850.91), and their gender ratio was 1.81. Over this period from 1990 to 2019, there was a consistent upward trend in crude deaths from CVDs and their corresponding number of DALYs, ASDR (EAPC = 0.06), and age-standardized DALYs (EAPC = 0.30) worldwide due to exposure to ambient PM pollution, as shown in Table 1.

**Table 1:** The global CVD burden due to ambient particulate matter pollution from 1990 to 2019 in different regions.

Characteristics	1990						2019						EAPC (1990–2019)	
	Death cases	ASDR per 10,000		DALYs	Age-standardized DALY rate per 10,000		Death cases	ASDR per 10,000		DALYs	Age-standardized DALY rate per 10,000		ASDR	Age-standardized DALY rate
	No. (95%UI)	No. (95%UI)	Male/female	No. (95%UI)	No. (95%UI)	Male/female	No. (95%UI)	No. (95%UI)	Male/female	No. (95%UI)	No. (95%UI)	Male/female	No. (95%CI)	No. (95%CI)
Global	1115609.56 (808072.92, 1442889.58)	30.75 (22.33, 39.66)	1.54	27446835.53 (19940175.10, 36011833.88)	681.83 (494.72, 888.87)	1.71	2475395.02 (2051050.10, 2871325.06)	30.85(25.52, 35.78)	1.71	60910841.13 (50072396.80, 70319155.87)	737.21 (606.86, 850.91)	1.81	0.06 (−0.01, 0.13)	0.30 (0.23, 0.38)
Sex	–	–	–	–	–	–	–	–	–	–	–	–	–	–
Female	492053.06 (354661.45, 635608.72)	24.61 (17.71, 31.82)	–	10794529.49 (7727342.10, 14305911.54)	507.90 (361.42, 671.61)	–	1013790.91 (818744.93, 1213728.78)	23.15 (18.71, 27.72)	–	22991745.53 (18718210.56, 27367406.75)	529.51 (430.51, 629.95)	–	−0.24 (−0.29, −0.19)	0.09 (0.04, 0.15)
Male	623556.50 (447043.72, 813718.08)	37.88 (27.08, 48.89)	–	16652306.03 (11972641.81, 21998875.99)	869.38 (628.57, 1133.87)	–	1461604.12 (1205992.74, 1699214.75)	39.62 (32.66, 46.02)	–	37919095.60 (30830629.10, 44189975.70)	959.33 (782.41, 1116.63)	–	0.26 (0.17, 0.35)	0.43 (0.34, 0.52)
Sociodemographic index	–	–	–	–	–	–	–	–	–	–	–	–	–	–
Low SDI	23050.82 (8255.98, 48790.33)	10.77 (3.97, 22.36)	1.87	657019.85 (234711.27, 1379522.42)	256.67 (92.73, 539.95)	1.93	109645.96 (68428.77, 159433.51)	22.84 (14.36, 33.04)	1.68	3087062.30 (1900859.03, 4487795.54)	543.34 (338.04, 792.56)	1.74	2.98 (2.78, 3.17)	2.98 (2.79, 3.17)
Low-middle SDI	86688.15 (40399.26, 155036.04)	16.06 (7.61, 28.48)	1.73	2450431.65 (1123451.94, 4446849.31)	380.36 (176.67, 684.41)	1.84	485314.74 (345662.29, 609832.62)	37.46 (26.96, 47.03)	1.69	13029044.36 (9194241.94, 16439228.91)	905.19 (639.92, 1141.44)	1.79	3.25 (3.13, 3.38)	3.36 (3.22, 3.50)
Middle SDI	304555.91 (197656.72, 428158.00)	33.32 (21.70, 46.73)	1.60	8354228.33 (5503237.96, 11712029.21)	768.04 (503.11, 1079.14)	1.66	1073672.98 (903949.90, 1234176.88)	46.89 (39.41, 54.05)	1.64	26767987.51 (22614856.37, 30573383.80)	1058.63 (895.55, 1208.40)	1.74	1.35 (1.12, 1.59)	1.25 (1.04, 1.46)
High-middle SDI	446652.41 (316899.77, 570385.63)	45.65 (32.54, 58.54)	1.55	10561492.68 (7533995.55, 13508486.11)	988.11 (707.69, 1265.54)	1.77	660131.58 (549539.83, 770732.37)	32.90 (27.39, 38.46)	1.75	14776311.85 (12339655.28, 17032130.25)	736.04 (614.41, 847.36)	1.89	−1.23 (−1.46, −1.00)	−1.17 (−1.41, −0.93)
High SDI	254186.80 (161478.46, 356862.95)	24.24 (15.44, 33.91)	1.71	5412149.83 (3538702.94, 7448080.65)	529.86 (348.54, 724.54)	1.91	145713.69 (109251.66, 186322.16)	7.57 (5.75, 9.55)	1.92	3226941.99 (2488358.67, 4038534.33)	197.15 (153.01, 245.66)	2.03	−4.31 (−4.44, −4.18)	−3.65 (−3.79, −3.51)
Region	–	–	–	–	–	–	–	–	–	–	–	–	–	–
Andean Latin America	3966.79 (1874.76, 6696.16)	20.29 (9.57, 34.37)	1.61	105659.72 (50094.63, 177366.66)	474.26 (223.09, 797.08)	1.65	7959.49 (5525.10, 10721.21)	14.48 (10.04, 19.64)	1.43	195451.50 (136815.91, 262055.76)	338.83 (236.79, 455.10)	1.51	−1.07 (−1.45, −0.69)	−1.10 (−1.46, −0.73)
Australasia	1980.83(221.03,4650.47)	8.59(0.97,20.39)	1.74	41763.90(4780.02, 98527.09)	179.83(20.58, 423.01)	1.98	1100.54(270.83, 2031.69)	2.08(0.52,3.79)	1.76	20350.22(5080.68, 36753.90)	44.03(10.80,80.05)	1.96	−5.45(−5.76, −5.14)	−5.31(−5.62, −4.99)
Caribbean	5668.40 (2329.58, 9992.05)	22.58 (9.22, 39.76)	1.40	135138.37 (55697.49, 236463.68)	508.62 (208.90, 893.59)	1.51	9724.79 (5297.78, 15581.98)	18.80 (10.25, 30.18)	1.51	230422.42 (124959.80, 373461.82)	446.31 (241.89, 721.91)	1.63	−0.65 (−0.90, −0.39)	−0.44 (−0.70, −0.19)
Central Asia	23048.69 (11867.48, 38710.58)	52.81 (27.15, 88.92)	1.77	571485.05 (296612.85, 944720.21)	1201.16 (627.24, 1994.91)	1.97	51384.46 (36499.57, 67267.05)	80.34 (57.52, 105.38)	1.63	1316956.41 (935489.90, 1729342.16)	1739.73 (1237.81, 2274.08)	1.82	1.09 (0.72, 1.47)	0.86 (0.48, 1.24)
Central Europe	93964.06 (52196.41, 138468.93)	67.81 (37.41, 100.24)	1.68	2115771.25 (1179963.93, 3096897.95)	1459.01 (810.87, 2134.89)	1.97	67116.42 (54443.88, 80960.41)	30.48 (24.69, 36.59)	1.65	1287810.86 (1044194.41, 1538824.30)	628.18 (511.21, 753.28)	1.91	−3.18 (−3.39, −2.97)	−3.36 (−3.57, −3.15)
Central Latin America	17055.52 (8703.50, 27972.25)	22.00 (11.18, 36.02)	1.42	445661.45 (229227.06, 715675.42)	497.38 (255.62, 803.57)	1.52	38152.38 (28658.96, 48208.65)	16.56 (12.42, 20.98)	1.60	914327.32 (691804.72, 1156170.52)	379.69 (287.80, 479.56)	1.77	−1.28 (−1.45, −1.11)	−1.23(−1.40, −1.06)
Central sub-Saharan Africa	1992.01 (631.91, 4453.25)	10.09 (3.28, 22.62)	1.68	56713.97 (17765.32, 129076.51)	233.68 (74.21, 529.33)	1.86	8265.14 (4049.47, 14536.16)	17.73 (8.82, 30.79)	1.53	235427.83 (113839.80, 416956.31)	407.58 (199.60, 715.09)	1.67	1.71 (1.25, 2.17)	1.68 (1.22, 2.15)
East Asia	230327.73 (109663.45, 386178.67)	30.97 (15.02, 51.55)	1.74	6035807.79 (2850487.70, 10110655.25)	677.66 (321.12, 1137.37)	1.73	933674.32 (764349.81, 1118569.42)	49.79 (40.83, 59.47)	1.84	21409338.85 (17377566.76, 25558253.25)	1050.67 (855.62, 1248.94)	1.87	2.06 (1.67, 2.44)	1.86 (1.53, 2.20)
Eastern Europe	162648.23 (75452.52, 262449.77)	62.30 (28.92, 101.00)	1.64	3618887.37 (1697765.84, 5796624.09)	1318.83 (615.75, 2119.65)	1.92	114066.31 (71761.28, 156339.22)	33.02 (20.89, 45.30)	1.83	2416937.36 (1522624.50, 3306189.12)	728.9 2(461.98, 993.14)	2.22	−2.49 (−3.06, −1.92)	−2.43 (−3.05, −1.80)
Eastern sub-Saharan Africa	3460.21 (1225.75, 8096.61)	5.14 (1.89, 11.79)	1.89	98471.30 (34604.34, 234252.24)	121.67 (43.29, 284.72)	2.01	15225.67 (8002.34, 25541.44)	10.51 (5.52, 17.59)	1.75	424358.57 (223980.28, 714305.00)	240.13 (127.92, 405.09)	1.91	2.88 (2.72, 3.04)	2.72 (2.58, 2.87)
High-income Asia Pacific	34905.63 (14732.17, 59357.84)	18.46 (7.65, 31.59)	1.50	853088.54 (378722.47, 1416020.82)	425.10 (188.22, 708.20)	1.63	31228.06 (22222.15, 41175.89)	6.36 (4.71, 8.27)	1.95	644787.11 (481481.87, 838456.45)	169.51 (127.40, 219.19)	1.93	−3.93 (−4.30, −3.55)	−3.35 (−3.70, −3.01)
High-income North America	66178.22 (24944.78, 119589.42)	18.53 (7.01, 33.40)	1.80	1422003.66 (541198.37, 2534947.28)	419.29 (160.35, 744.56)	1.96	27606.31 (14617.18, 42191.44)	4.32 (2.29, 6.59)	1.74	599714.40 (319673.38, 904875.10)	104.83 (56.44, 158.51)	1.80	−5.53 (−5.82, −5.22)	−5.18 (−5.43, −4.92)
North Africa and middle East	103381.94 (81600.58, 124783.42)	65.89 (52.35, 79.54)	1.37	2867384.57 (2269892.52, 3468804.61)	1548.38 (1225.57, 1873.71)	1.49	253355.47 (211407.43, 298025.71)	63.66 (53.44, 74.27)	1.25	6771940.54 (5618660.23, 8034634.77)	1459.36 (1219.47, 1715.54)	1.37	−0.23 (−0.36, −0.11)	−0.34 (−0.46, −0.22)
Oceania	251.91 (72.34, 646.15)	8.86 (2.59, 22.60)	2.26	8193.81 (2355.02, 21027.75)	234.03 (67.96, 591.82)	2.48	928.38 (293.40, 2075.02)	13.51 (4.41, 30.63)	1.95	30130.98 (9473.68, 67862.66)	357.38 (115.43, 803.71)	2.17	1.39 (1.28, 1.51)	1.40 (1.28, 1.52)
South Asia	112860.23 (52073.19, 196693.90)	21.81 (10.07, 37.81)	1.81	3306918.19 (1508748.55, 5797687.82)	526.34 (241.80, 921.48)	1.95	599910.02 (457265.15, 734409.91)	44.87 (34.54, 54.90)	1.64	16438120.40 (12430524.99, 20148884.19)	1099.64 (832.62, 1349.27)	1.75	2.77 (2.64, 2.91)	2.88 (2.73, 3.04)
Southeast Asia	48799.42 (22174.37, 85276.97)	20.75 (9.42, 36.34)	1.67	1433299.29 (660324.88, 2480713.61)	513.11 (235.04, 889.08)	1.75	166561.68 (126152.89, 208668.21)	29.27 (22.18, 36.72)	1.67	4648949.30 (3550992.42, 5810741.45)	721.08 (548.82, 899.46)	1.83	1.24 (0.92, 1.55)	1.22 (0.92, 1.52)
Southern Latin America	10795.27 (4466.37, 18948.97)	24.40 (10.07, 42.78)	1.80	258982.66 (109385.14, 450659.63)	560.17 (236.37, 973.82)	2.00	9804.10 (6854.76, 13009.13)	11.68 (8.19, 15.51)	1.79	218101.82 (151554.70, 291346.30)	269.10 (187.31, 359.14)	1.94	−2.62 (−2.89, −2.36)	−2.64 (−2.89, −2.40)
Southern sub-Saharan Africa	5289.00 (3851.71, 6929.22)	20.00 (14.42, 26.35)	1.61	156805.07 (114838.45, 201507.06)	512.24 (374.45, 664.17)	1.76	12789.47 (9867.43, 15962.16)	25.07 (19.33, 31.33)	1.47	336619.36 (263130.19, 418546.20)	572.00 (444.13, 714.14)	1.67	0.90 (0.45, 1.36)	0.48 (0.04, 0.92)
Tropical Latin America	18414.84 (8392.44, 32278.21)	21.00 (9.58, 37.29)	1.67	529153.09 (242808.59, 924109.09)	524.87 (240.07, 917.09)	1.82	24480.46 (17191.42, 32360.95)	10.24 (7.19, 13.54)		635961.83 (449134.32, 836490.95)	256.63 (181.35, 337.24)	1.75	−2.55 (−2.78, −2.31)	−2.57 (−2.80, −2.33)
Western Europe	160779.40 (73307.46, 262064.79)	27.24 (12.48, 44.28)	1.74	3132065.69 (1455816.07, 5052472.54)	559.05 (261.08, 897.14)	2.03	61381.11 (44714.31, 79429.05)	6.02 (4.40,7.70)	1.77	1053989.22 (776003.60, 1342912.41)	124.83 (92.37,159.49)	2.02	−5.40 (−5.51, −5.28)	−5.32 (−5.43, −5.22)
Western sub-Saharan Africa	9841.22(4335.36, 19185.75)	12.98(5.81, 25.23)	1.37	253580.78(110130.17, 503613.16)	281.36(123.64, 553.15)	1.47	40680.43(26461.27, 58529.65)	25.19(16.50, 35.75)	1.24	1081144.85(680273.47, 1576408.53)	544.94(344.45, 786.83)	1.34	2.43 (2.24, 2.62)	2.42 (2.23, 2.60)

### 3.2 CVD burden linked to exposure to ambient PM pollution among different genders and age groups

**Figure 1:**
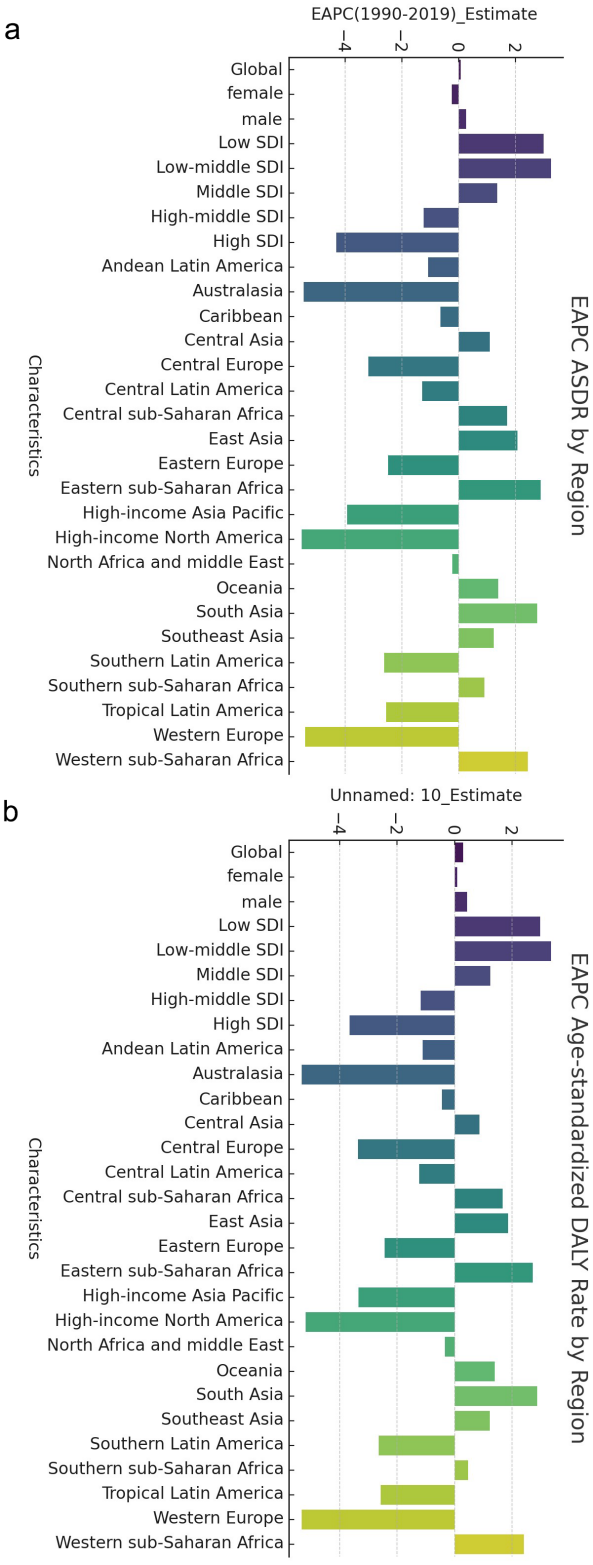
EAPC of global cerebrovascular disease burden due to ambient particulate matter pollution, by regions. (a) EAPC of ASDR; (b) EAPC of age standardized DALY rate. ASDR, age standardized death rate; DALY, disability adjusted life-year.

Globally, in both 1990 and 2019, the crude mortality rate from CVDs, the total number of DALYs attributed to CVDs, the ASDR for CVDs, and the age-standardized DALYs due to CVDs associated with exposure to ambient PM pollution were higher in men than in women (Table 1). When comparing data from 1990 to 2019, it was observed that while the crude mortality rate from CVDs and the total number of DALYs due to CVDs related to exposure to ambient PM pollution increased in women, the ASDR from CVDs decreased (EAPC = −0.24 [−0.29, −0.19]) and the age-standardized DALYs due to CVDs slightly increased (EAPC = 0.09 [0.04, 0.15]). Conversely, when comparing data from 1990 to 2019 in men, there was a significant increase in the crude number of deaths from CVDs, the total number of DALYs due to CVDs, the ASDR for CVDs (EAPC = 0.26 [0.17, 0.35]), and the age-standardized DALYs due to CVDs related to exposure to ambient PM pollution (EAPC = 0.43 [0.34, 0.52]) (Table 1; Figure 1).

**Figure 2:**
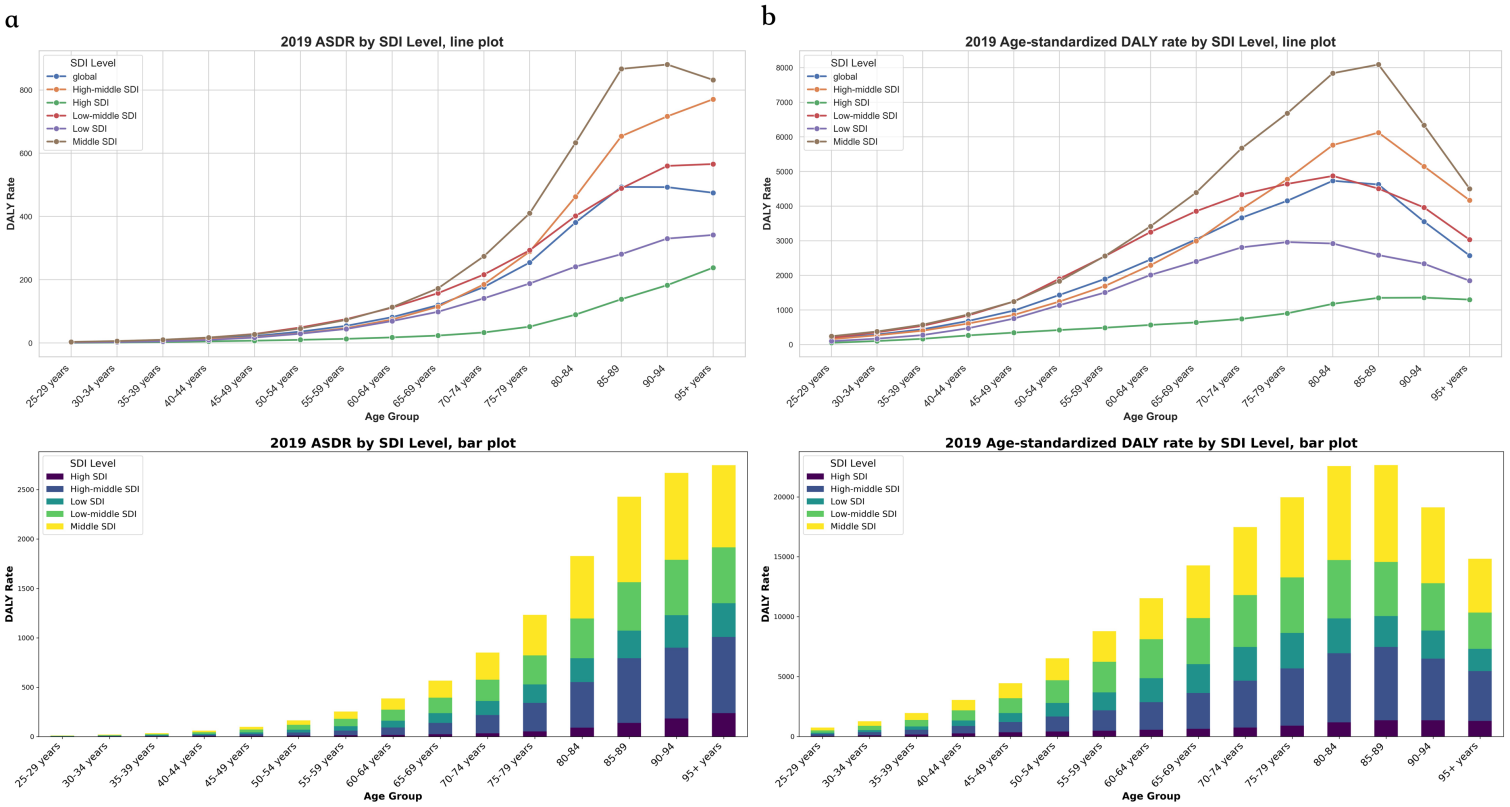
The Rate for global cerebrovascular disease burden due to ambient particulate matter pollution, by ages and regions. (a) Death rate; (b) DALY rate. DALY, disability adjusted life-year.

The global mortality rate for CVDs associated with exposure to ambient PM pollution demonstrated an upward trend with increasing age, peaking in individuals aged 85–89, and subsequently remaining stable in older age groups. The DALYs attributed to CVDs linked to ambient PM pollution also reached their highest point in individuals aged 80–84, followed by a gradual decline in those aged 85–89, and a rapid decrease in those over the age of 89. Across all regions, the ASDR for CVDs related to exposure to ambient PM pollution was most pronounced in individuals aged 75–89, succeeded by those aged 90–94. Furthermore, the proportion of DALYs due to CVDs associated with exposure to ambient PM pollution was highest (exceeding 50%) in individuals aged 65–89 (Figures 2a,b).

### 3.3 CVD burden linked to exposure to ambient PM pollution in various countries and areas

In 1990, the highest crude mortality, number of DALYs, ASDR and age-standardized DALYs for CVDs associated with exposure to ambient PM pollution were observed in high–middle-SDI areas, followed by middle-SDI areas and low-SDI areas. In 2019, the highest crude mortality, number of DALYs, ASDR and age-standardized DALYs were observed in middle-SDI areas, followed by high–middle-SDI areas and then high-SDI areas (which had much lower values than other areas). The mortality rates and DALYs in different SDI areas were higher in men than in women. Overall, from 1990 to 2019, the ASDRs and age-standardized DALY for CVDs associated with exposure to ambient PM pollution in low-SDI, low–middle-SDI and middle-SDI areas increased continuously, with the fastest increases observed in low–middle-SDI areas (ASDR: EAPC = 3.25 [3.13, 3.38]; age-standardized DALYs: EAPC = 3.36 [3.22, 3.50]; Table 1; Figure 1).

In contrast, the ASDRs and age-standardized DALYs for CVDs associated with exposure to ambient PM pollution in high–middle- and high-SDI areas decreased during 1990–2019, with the most pronounced declines observed in high-SDI areas (ASDR: EAPC = −4.31 [−4.44, −4.18]; age-standardized DALYs: EAPC = −3.65 [−3.79, −3.51]; Table 1; Figure 1).

**Figure 3:**
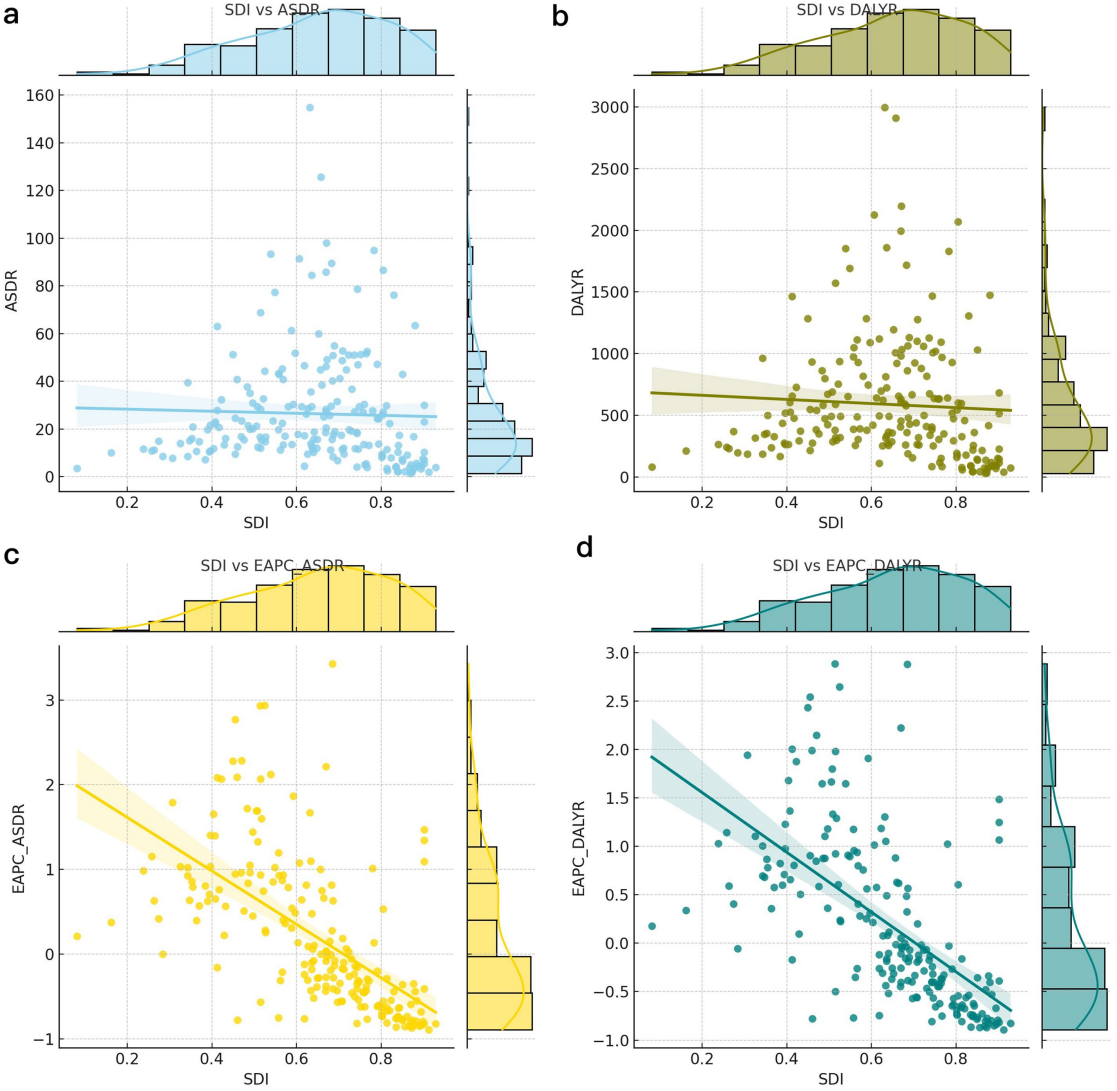
The relationship between different rates and SDI for global cerebrovascular disease burden due to ambient particulate matter pollution. (a) ASDR; (b) DALYR; (c) EAPC of ASDR (d) EAPC of age standardized DALY rate. DALY, disability adjusted life-year; ASDR, age standardized death rate.

The correlation analysis between SDI and ASDR, age-standardized DALYs, and corresponding EAPCs revealed that as the SDI increased, there was a slight downward trend in both ASDR and age-standardized DALYs for CVDs associated with exposure to ambient PM pollution (Figures 3a,b). Conversely, the EAPCs for these parameters exhibited a significant downward trend (Figures 3c,d). With advancing age, the ASDR for CVDs linked to ambient PM pollution consistently rose across each SDI region. For individuals aged over 49, the ASDR for CVDs in each age group within high-SDI areas was markedly lower than in their counterparts in other-SDI regions. However, for those aged over 69, the ASDR for CVDs in each age group within high–middle-SDI areas surged, surpassing the national average. The ASDR for CVDs was notably higher in low–middle-SDI areas compared to all other age groups and regions, except for those aged 70–74 in high–middle-SDI areas. Among all age cohorts, low-SDI areas had the highest ASDR for CVDs related to ambient PM exposure, succeeded by low–middle-SDI areas (Figure 2a). In general, age-standardized DALYs due to CVDs across various SDI regions initially increased and then decreased with age. High-SDI areas reported the lowest age-standardized DALYs for CVDs, which gradually escalated with age; low-SDI areas reached their peak age-standardized DALYs among those aged 70–74 years, followed by a decline in older age groups; and low–middle-SDI areas peaked at age-standardized DALYs among those aged 75–79. Furthermore, age-standardized DALYs due to CVD in high–middle-SDI areas exceeded the national average for those aged 65–69 and then rapidly surged to a peak among those aged 80–84. The highest age-standardized DALYs due to CVDs across all age groups were observed in low-SDI areas, followed by low–middle-SDI areas (Figure 2b).

In 1990, the three regions with the highest crude death number due to CVDs associated with exposure to ambient PM pollution were East Asia (230,327.73 [109,663.45, 386,178.67]), Eastern Europe (162648.23 [75452.52, 262,449.77]), and Western Europe (160,779.4 [73,307.46, 262,064.79]). The highest ASDRs for CVDs were in Central Europe (67.81 [37.41, 100.24]), North Africa and the Middle East (65.89 [52.35, 79.54]), and Western Europe (62.3 [28.92, 101.00]). The three regions with the highest DALYs due to CVDs were East Asia (6035807.79 [2,850,487.71, 11,110,655.25]), Western Europe (3,618,887.37 [1,697,765.84, 5,796,624.09]), and South Asia (3,306,918.19 [1,508,748.55, 5,797,687.82]). The regions with the highest age-standardized DALYs due to CVDs were North Africa and the Middle East (1548.38 [1,225.57, 1,873.71]), Central Europe (1,459.01 [810.87, 2,134.89]), and Eastern Europe (1318.83 [615.75, 2,119.65]). In 2019, the three regions with the highest ASDRs for CVDs were Central Asia (80.34), North Africa and Middle East (63.66), and East Asia (49.79), while the three regions with the lowest ASDRs for CVDs were Western Europe (6.02), High-income North America (4.32), and Oceania (2.08). The three regions with the highest age-standardized DALYs due to CVDs were Central Asia (1739.73), North Africa and the Middle East (1459.36), and South Asia (1099.64), while the three regions with the lowest age-standardized DALYs due to CVDs were Western Europe (124.83), High-income North America (104.83), and Oceania (44.03).

**Figure 4:**
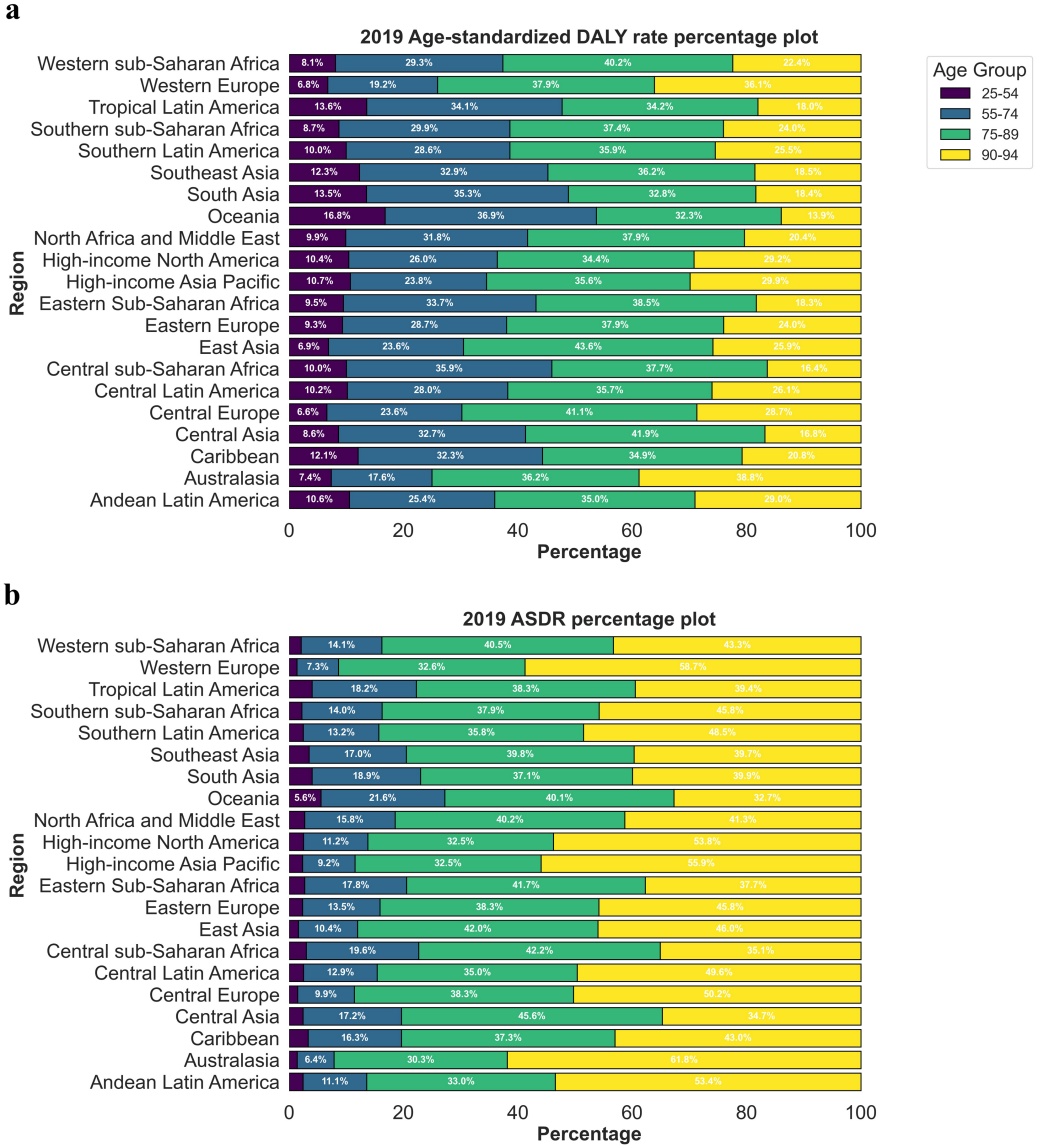
The proportion ASRs of global cerebrovascular disease burden due to ambient particulate matter pollution in 2019, by age groups and regions. (a) ASDR; (b) age-standardized DALY rate. ASDR, age standardized death rate; DALY, disability adjusted life-year.

Between 1990 and 2019, the three regions with the most significant increase in crude mortality rates due to CVDs associated with exposure to ambient PM pollution were South Asia (5.32), Eastern sub-Saharan Africa (4.40), and Central sub-Saharan Africa (4.15). The three regions with the most substantial rise in the number of DALYs due to CVDs were South Asia (4.97), Eastern sub-Saharan Africa (4.31), and Western sub-Saharan Africa (4.26). Conversely, the three regions with the most notable decrease in the number of DALYs due to CVDs were Australasia (death number: 0.56; DALYs: 0.49), High-income North America (death number: 0.42; DALYs: 0.42), and Western Europe (death number: 0.38; DALYs: 0.34). The three regions with the most rapid increase in the ASDRs and age-standardized DALYs due to CVDs were Eastern sub-Saharan Africa (EAPC = 2.88; 2.72), South Asia (EAPC = 2.77; 2.88), and Western sub-Saharan Africa (EAPC = 2.43; 2.42). Meanwhile, the three regions with the most pronounced decrease in the ASDRs and age-standardized DALYs due to CVDs were High-income North America (EAPC = −5.53; −5.18), Oceania (EAPC = −5.45; −5.31), and Western Europe (EAPC = −5.40; −5.32) (Table 1). Among the 21 geographical areas, those aged between 75 and 89 had the highest ASDR due to CVDs, accounting for approximately 50%, followed by those aged between 90 and 94. Those aged under 54 had the lowest ASDR due to CVDs, accounting for less than 10% (Figure 4a). The age-standardized DALYs were highest in those aged between 65 and 79, followed by those aged between 80 and 90, while they were lowest in those aged between 25 and 44, accounting for no more than 10% (Figure 4b).

**Figure 5:**
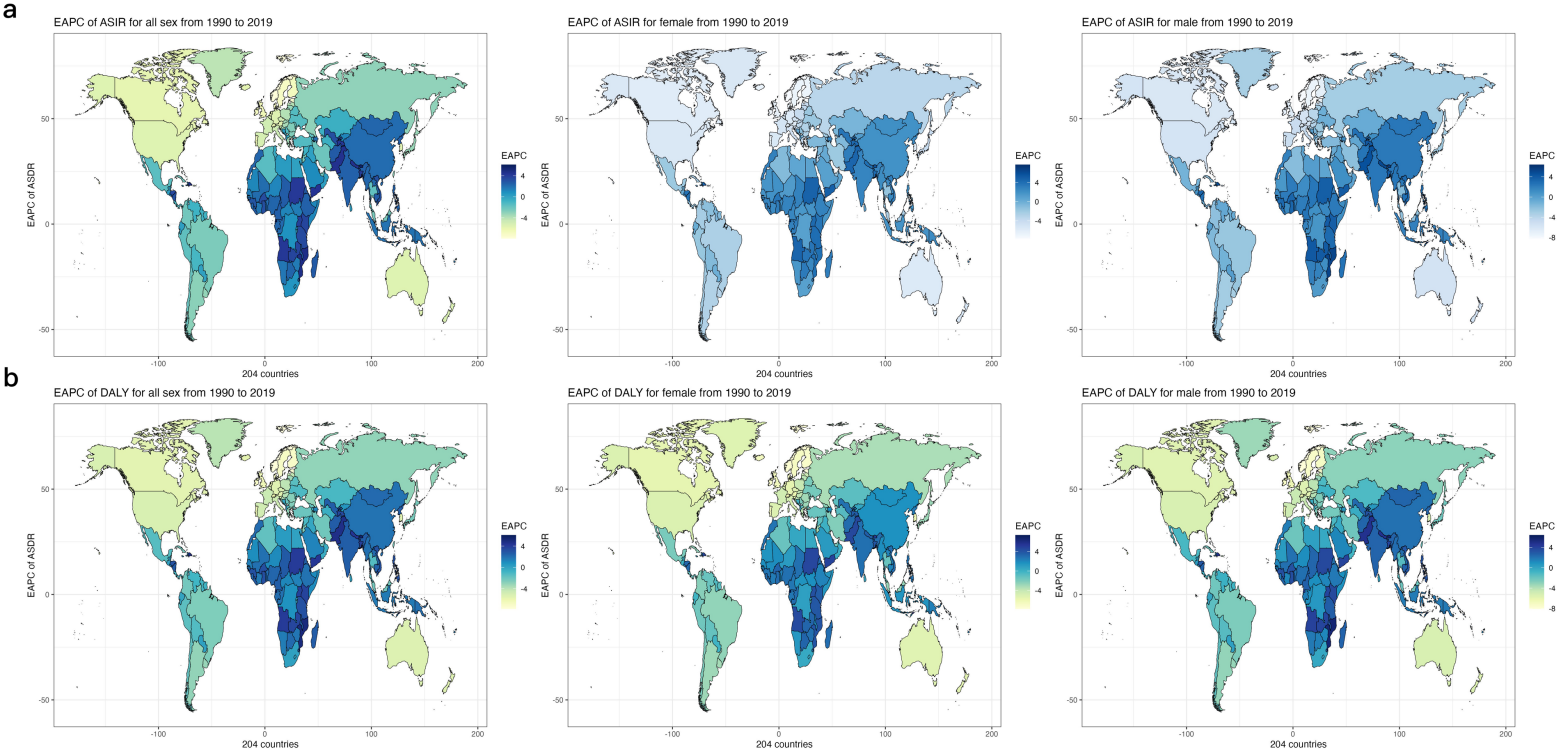
The EAPC Heat Map of global cerebrovascular disease burden due to ambient particulate matter pollution for both genders in 204 countries from 1990 to 2019. (a) The EAPC of ASDR (b) The EAPC of age-standardized DALY rate. EAPC, estimated annual percentage change; ASDR, age standardized death rate.

In 2019, Iraq (98.04; 2198.11), Egypt (125.55; 2912.41) and Uzbekistan (154.77; 2996.10) had the highest ASDRs and age-standardized DALYs due to CVDs associated with exposure to ambient PM pollution among the 204 countries worldwide, while Iceland (1.46; 30.88), Sweden (1.68; 34.58) and Norway (1.91; 41.25) had the lowest values of these parameters. From 1990 to 2019, Equatorial Guinea (9.86; 9.28), Timor-Leste (12.12; 10.22) and Djibouti (12.95; 12.21) had the highest crude mortality and DALYs increase, while Norway (0.16; 0.16), Sweden (0.19; 0.18) and Estonia (0.20; 0.17) had the lowest increase in crude mortality and DALYs among the countries worldwide from 1990 to 2019.From 1990 to 2019, Bhutan (EAPC = 5.66; 5.41), Timor-Leste (EAPC = 6.09; 6.06) and Equatorial Guinea (EAPC = 6.10; 5.58) had the fastest increasing trends in ASDRs and age-standardized DALYs, while Norway (EAPC = −7.93), Estonia (EAPC = −7.39) and Sweden (EAPC = −7.10) had the fastest decreasing trends in ASDRs among the countries worldwide from 1990 to 2019, and Norway (EAPC = −7.81), Estonia (EAPC = −7.57) and Finland (EAPC = −7.09) had the fastest decreasing trends in age-standardized DALYs among the countries worldwide from 1990 to 2019. Figures 5a,b show the trend of ASDRs and age-standardized DALYs by country worldwide from 1990 to 2019, respectively.

## 4 Discussion

This study, based on the GBD 2019 database, provides a comprehensive analysis of the spatial pattern and temporal trends of CVD burden associated with exposure to ambient PM pollution across 204 countries and regions from 1990 to 2019. The findings indicate that between 1990 and 2019, there was a global increase in both CVD deaths and DALYs attributed to exposure to ambient PM pollution, consistent with the results reported by Rakesh et al. (18). Specifically, compared to 1990, the number of deaths and DALYs in 2019 increased by 121.9 and 121.9%, respectively, while the ADSR and age-standardized DALYs rose by 0.33 and 8.12%, respectively. However, these trends varied among different countries and regions. The primary driver of the significant rise in CVD deaths and DALYs due to exposure to ambient PM pollution is attributed to global population growth and aging.

Between 1990 and 2019, the burden of CVD associated with exposure to ambient PM pollution was higher in males than in females, displaying an increasing trend. Conversely, the ASDR for women significantly decreased. This pattern aligns with previous research by He and Guo et al., indicating that men with CVDs have a poorer prognosis than women with CVDs. Studies have shown that smoking rates are considerably higher in males than in females, and young men are exposed to more environmental PM in their workplaces than women. The protective effects of estrogen on the cardiovascular system may contribute to the higher burden of CVDs in males (19–21). The burden of CVDs related to ambient PM pollution also varied among different age groups, with both ASDRs and age-standardized DALYs increasing with age. As individuals age, blood vessels can degrade due to damage and reduced activity of endothelial cells, leading to endothelial dysfunction in older adults (22). Numerous studies have identified ambient PM pollution as a risk factor for endothelial and microvascular dysfunction. This is associated with the acute endothelial response, which triggers the release of endothelin-1, resulting in vasoconstriction (23), and increases in fibrinogen concentration and plasma viscosity (24). Other animal researches have shown a connection between air pollution and an increase in biomarkers of systemic inflammation, such as C-reactive proteins or pro-inflammatory cytokines. For example, a study during the Beijing Olympics found that strict restrictions on air pollution were associated with significant and rapid reductions in concentrations of biomarkers of inflammation and thrombosis (24). The physical regulation and metabolic ability, immunity, and resistance are lower in elder people than in younger people, which may account for the higher burden of CVDs linked to ambient PM pollution in older adult than in younger populations.

It is important to note that our analysis assumes the exposure-response relationship between PM pollution and CVD outcomes is uniform across all regions and populations. While this simplifies the analysis, it may not fully account for regional differences in susceptibility, baseline health conditions, or environmental factors. Variations in socioeconomic status, healthcare access, or genetic predispositions could lead to underestimation or overestimation of the true burden in some areas. Although we did not conduct sensitivity analyses to test this assumption, we acknowledge this limitation and suggest that future research should explore these regional differences to refine estimates of CVD burden related to PM exposure.

PM is a complex and heterogeneous mixture that serves as a significant environmental mediator for the high global morbidity and mortality associated with cardiovascular disease. It is typically classified based on size into coarse (aerodynamic diameter < 10 μm; PM10), fine (diameter < 2.5 μm; PM_2.5_), or ultrafine (<0.1 μm; PM0.1) (25–27). Among these, PM_2.5_ has been the most extensively studied and identified as particularly harmful air pollutant, with both short-term and long-term exposure linked to increased rates of CVD (28, 29). A British cohort study found that CVD mortality risk rose by 30 and 16% for every 10 μg/m3 increase in PM_2.5_ and PM_10_ concentrations over a one -year period, respectively (30). A recent systematic review of 104 cohort studies reported a pooled risk ratio for CVD mortality per 10 μg/m3 increase in PM_2.5_ exposure of 1.11 (31). Furthermore, a prospective cohort study from the Netherlands demonstrated that long-term exposure to ultrafine particles was associated with an increased risk for all incident CVD (HR: 1.18, 95%CI: 1.03–1.34), myocardial infarction (HR: 1.34, 95%CI: 1.00–1.79), and heart failure (HR: 1.76, 95%CI: 1.17–2.66) (32).

Several mechanisms may elucidate the associations between PM exposure and adverse cardiovascular effects. Firstly, autonomic mechanisms are implicated; PM deposited in the pulmonary tree can directly stimulate lung nerve reflexes via irritant receptors, leading to an imbalance in the autonomic control of the heart and a reduction in heart rate variability. This is a significant risk factor for sudden death and severe arrhythmias. Secondly, the inhalation of PM can trigger the release of pro-oxidative and/or pro-inflammatory biological mediators, acute-phase reactants, and vasoactive hormones from the lungs into the systemic circulation. These substances can then indirectly mediate cardiovascular responses. Thirdly, PM can inhibit the production of the endogenous vasodilator nitric oxide, which may contribute to increased blood pressure due to its reduced bioavailability. Finally, nanoscale particles and/or soluble PM constituents can translocate into the systemic circulation after inhalation, where they directly interact with the cardiovascular system. The primary mechanisms are illustrated in Supplementary Figure S1.

Inhaled PM_2.5_ deposit deep in pulmonary tissues (e.g., alveoli), interact/activate local cells (e.g., resident macrophages, dendritic cells, alveolar/endothelial cells) and modify endogenous structures (e.g., cell membranes, surfactant lipids, antioxidants) (33). Exposure to these pollutants can cause oxidative stress via a variety of cellular mechanisms including uncoupling of nitric oxide synthetase (producing reactive nitrogen species), mitochondrial dysfunction and formation of reactive oxygen species (34). Pro-oxidant species can be directly generated from certain particles within biological tissues (eg, metals) or from lung-based cells in response to interactions with PM (eg, NADPH oxidase of resident macrophages) or both. Redox-sensitive pathways (eg, nuclear factor κB) become activated leading to the production of proinflammatory cytokines (eg, IL-6 and Tumor necrosis factor *α*) and chemokines that amplify the immune response (as well as the oxidative stress), presumably as part of the coordinated attempt to clear or sequester particles (35). In addition, a “spillover” of proinflammatory or oxidative stress mediators generated in the lungs into the systemic circulation, activation of alveolar sensory receptors leads to the triggering of neural afferent events resulting in a decrease in parasympathetic activity, an increase in sympathetic activity or the release of endocrine molecules, triggering an imbalance in the autonomic nervous system and the penetration of certain particles or components directly into cardiovascular tissues (33, 36). Finally, numerous subclinical physiological alterations induced by PM inhalation including endothelial dysfunction, vasoconstriction, heightened arrhythmia potential and prothrombotic and coagulant hematologic changes likely determine the actual ostensible cardiovascular event (eg, stroke, heart failure or Acute Coronary Syndromes) triggered in any individual (37) (Supplementary Figure S2).

A minor proportion of particles directly enter the circulation, where elevated levels of PM_2.5_ penetrate the blood vessel interiors. These fine particles stimulate the expression of various adhesion molecules and induce cell activation. Furthermore, endothelial damage escalates the release of inflammatory cytokines, leading to the aggregation of blood mononuclear cells on activated endothelial monolayers and their infiltration into subendothelial spaces. This contributes significantly to the reduction in the function of vascular endothelial cells (VECs) that line the inner surfaces of the vasculature (38, 39) (Supplementary Figure S3).

The burden of CVDs associated with exposure to ambient PM pollution varied across areas with different SDIs. As SDI values increased, the overall CVD burden exhibited a slight downward trend, and the EAPCs in both mortality rate and DALYs rate significantly decreased. The CVD burden in low-SDI, low–middle-SDI, and middle-SDI areas demonstrated an upward trend, whereas it showed a downward trend in high–middle-SDI and high-SDI areas. In 2019, the highest CVD burden was observed in middle-SDI areas, while the lowest was found in high-SDI areas. Previous studies have indicated that over the past few decades, middle-SDI areas have experienced rapid population growth and urbanization and industrialization, which have had a significant impact on the environment. Consequently, the burden of various pulmonary, cardiovascular, cerebrovascular diseases, and cancers caused by environmental pollution is relatively high in these areas. The GBD 2019 also revealed that exposure to ambient PM pollution increased from low-SDI to middle-SDI areas, while it decreased in high-SDI areas (39). This may explain the decrease in the CVD burden in the latter areas, potentially due to changes in air quality management and energy use patterns. Industrialization in low-SDI to middle-SDI areas leads to an increase in ambient PM pollution, but in high-SDI areas, the implementation of pollution control policies results in a reduction in people’s exposure to this pollution (40, 41). Due to economic constraints, low-SDI areas are unlikely to prioritize the management of ambient PM pollution, leading to a clear upward trend in their pollution levels (42). Therefore, global regulatory policies should be tailored to low-to-middle-SDI areas with high and increasing exposure to ambient PM pollution, as this can have a profound impact on health conditions.

The impact of PM on public health is particularly pronounced in low- and middle-SDI regions, where rapid urbanization and industrialization have not been accompanied by effective pollution control. Studies have shown that these regions are burdened with high levels of pollution and limited healthcare resources (43, 44), which further exacerbates the effects of PM on public health. The economic costs of CVDs are substantial, as they lead to increased healthcare costs and lost productivity. Moreover, studies have shown that even long-term exposure to lower concentrations of PM2.5 can cause severe cardiovascular effects. These findings highlight the importance of implementing strict air quality standards and monitoring efforts (45, 46). Additionally, the interaction between PM pollution and other social determinants of health (e.g., socioeconomic status and access to healthcare) should be investigated to develop effective public health strategies. Future research should also focus on longitudinal studies to evaluate the long-term effects of air quality interventions and the role of policy changes in reducing the cardiovascular burden associated with PM pollution.

The spatial distribution of CVD burdens, associated with exposure to ambient PM pollution, varied significantly. In 2019, the disparities between regions with the highest and lowest ASDRs and age-standardized DALYs were most pronounced in Central Asia (38.6 times and 39.5 times respectively) and Oceania (39.5 times). The gap between the country with the highest ranking (Uzbekistan) and the lowest ranking (Iceland) was 106 times and 97 times, respectively. The GBD 2019 indicated that environmental PM exposure levels in Central Asia, including Uzbekistan, were considerably higher than those in Oceania and other regions (39). Given Central Asia’s geographical isolation from oceans, sparse precipitation, and extensive desert areas, it serves as a significant dust source in the Northern Hemisphere, thereby substantially impacting population health. Altyn et al. demonstrated that CVDs are the primary cause of death in Central Asia, where the mortality rate is generally higher than in other regions (47). However, Oceania, being surrounded by the sea, exhibits significantly lower concentrations of environmental PM compared to other regions. The minimum annual threshold for PM_2.5_ as stipulated in the air quality guidelines for Australasia is a mere 8 μg/m3. Between 1990 and 2019, Eastern sub-Saharan Africa, South Asia, and Western sub-Saharan Africa witnessed the most rapid escalation in CVD burden associated with ambient PM pollution. This surge may be attributed to the pronounced increase in CVD burden resulting from economic, medical, and climatic factors in the Sahara region. Lim et al. demonstrated that PM_2.5_ concentrations are on the rise in developing countries in South America and Southern sub-Saharan Africa, paralleling the population growth in the latter region (48). The regions exhibiting the most significant reductions in CVD burden associated with ambient PM pollution were High-income North America, Oceania, and Western Europe. These predominantly consist of developed nations that may reap benefits from advanced economic and health infrastructures, as well as robust environmental pollution control policies. Studies have indicated that PM_2.5_ concentrations in these regions are relatively low and on a declining trajectory. In conclusion, the CVD burden associated with ambient PM pollution exhibited variability across countries and regions from 1990 to 2019, attributable to differences in geography, economies, populations, and policy frameworks.

This study employed the GBD2019 database in a systematic manner for the first time to categorize the global burden and developmental trends of CVDs associated with ambient PM pollution, taking into account age, gender, and SDIs. The findings have significant implications for the creation of prevention and control strategies by national governments. However, despite the systematic annual updates of the global disease burden by the GBD database, it has certain limitations. These include a lack of high-quality data from low-income regions, which could potentially result in inaccurate estimations of disease burdens in these areas. Furthermore, this study only considered overall ambient PM pollution, neglecting the effects of PM of varying diameters. Future research could explore the impacts of all sizes of PMs.

## 5 Conclusion

In conclusion, over the past two decades, the global burden of CVDs associated with exposure to ambient PM pollution has increased, with a more pronounced rise in males and older adults compared to females and non-elderly individuals. As the SDI increased, there was a general decline in the overall CVD burden attributable to ambient PM exposure; however, regions with low and low-to-middleSDI levels experienced the most significant increase, highlighting the need for targeted interventions in these areas. Geographically, Central Asia, North Africa and the Middle East, East Asia, and South Asia bore the highest burden of CVDs due to ambient PM exposure, while Western Europe, high-income North America, and Oceania had the lowest. At the national level, Iraq, Egypt, and Uzbekistan were identified as having the highest burden of CVDs related to ambient PM exposure. Future efforts should focus on tailoring air-quality management strategies to local environmental conditions and disease profiles, and on enhancing CVD prevention and management to mitigate this burden.

## Data Availability

The original contributions presented in the study are included in the article/Supplementary material, further inquiries can be directed to the corresponding author.

## Footnotes

^1^ http://ghdx.healthdata.org/gbd-results-tool
