# Supplementary material for: Analysis of the global burden of cardiovascular diseases linked to exposure to ambient particulate matter pollution from 1990 to 2019
Source: Front Public Health. 2024 Oct 2;12:1391836. doi: 10.3389/fpubh.2024.1391836 (PMC11479877; doi:10.3389/fpubh.2024.1391836)
Supplement: Supplementary file 1 [file Data_Sheet_1.docx]

Supplementary Material

# Supplementary Figures and Tables

For more information on Supplementary Material and for details on the different file types accepted, please see [here](https://www.frontiersin.org/guidelines/author-guidelines#supplementary-material).

## Supplementary Figures


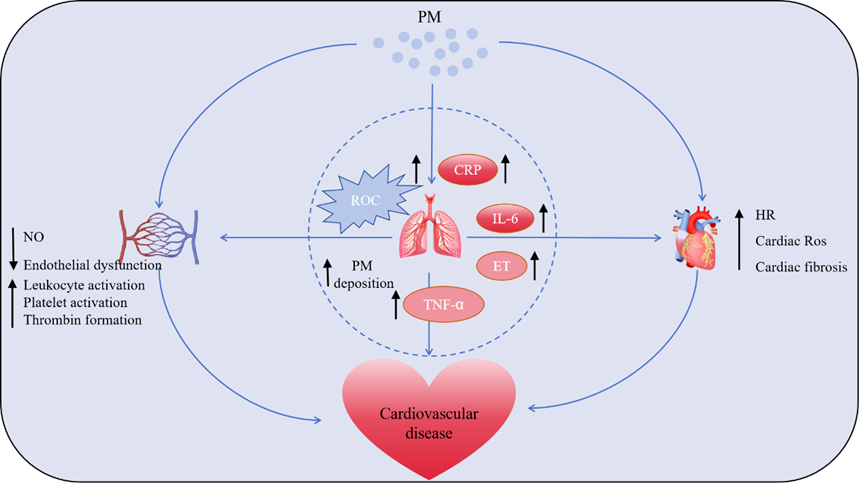


**Supplementary Figure 1.** Main pathogenic mechanisms of the inhalation of particulate matter (PM) on cardiovascular disease.

Note: PM, particulate matter; NO, nitric oxide; ROS, reactive oxygen species; HR, heart rate; CRP, C-reactive protein; IL-6, interleukin-6; ET, endothelins; TNF-α, tumor necrosis factor.

**
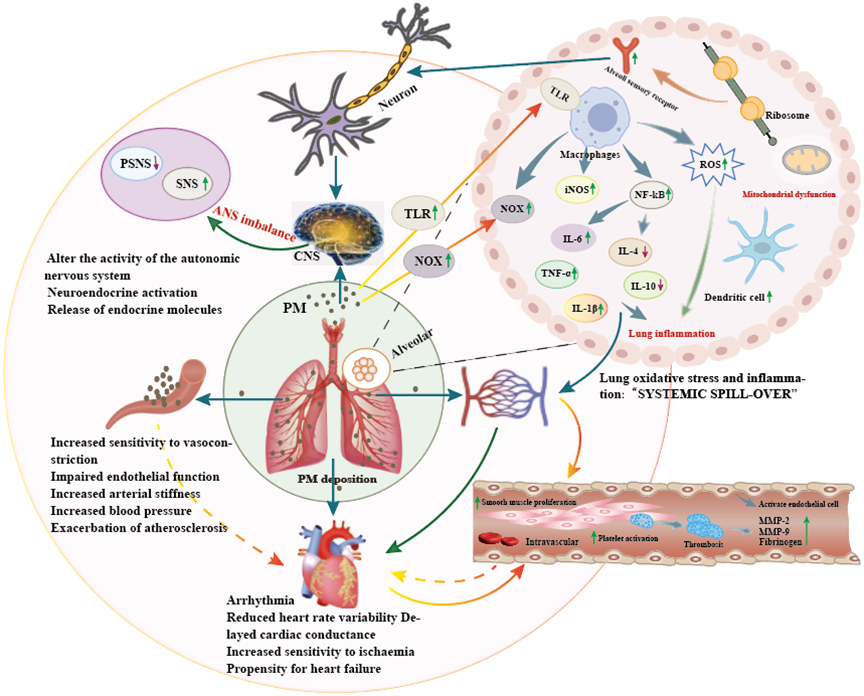
**

**Supplementary Figure 2.** Main pathogenic mechanisms of the inhalation of particulate matter (PM) on cardiovascular disease.

Note: PM, particulate matter; ANS, autonomic nervous system; CNS, central nervous system; SNS,sympathetic nervous system; PSNS, parasympathetic nervous system; ROS, reactive oxygen species; IL-4, interleukin-4; IL-6, interleukin-6; IL-10, interleukin-10; TNF-α, tumor necrosis factor; TLR, toll-like receptor; MMP, metalloproteinase; NOX, NADPH oxidase; NF-kB, nuclear factor kappa-B; iNOS, inducible nitric oxide synthase; The symbol ↑means increase, ↓means decrease.


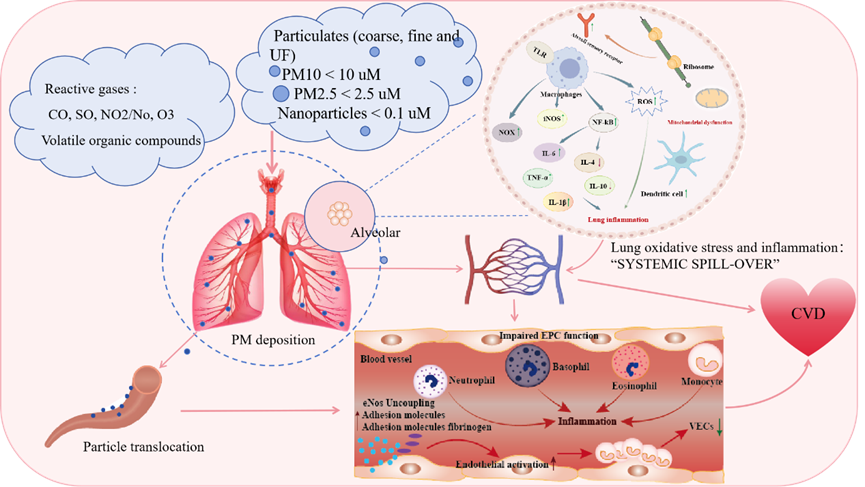


**Supplementary Figure 3.** Main pathogenic mechanisms of the inhalation of particulate matter (PM) on cardiovascular disease.

Note: PM, particulate matter; CO, carbon monoxide; SO, sulfur monoxide; NO, nitric oxide; NO2, nitrogen dioxide; O3, ozone; ROS, reactive oxygen species; IL-4, interleukin-4; IL-6, interleukin-6; IL-10, interleukin-10; TNF-α, tumor necrosis factor; TLR, toll-like receptor; MMP, metalloproteinase; NOX, NADPH oxidase; NF-kB, nuclear factor kappa-B; iNOS, Inducible nitric oxide synthase; eNOS, endothelial nitric oxide synthase; VECs, vascular endothelial cells; CVD, cardiovascular disease. The symbol ↑means increase, ↓means decrease.

## Supplementary Tables

**Supplementary Table 1.** The top three and the bottom three regions of global CVD burden due to ambient particulate matter pollution.

| Measure | sex | Top three regions | | | Bottom three regions | | |
| --- | --- | --- | --- | --- | --- | --- | --- |
| 2019 ASR (per 100,000 people) |  |  |  |  |  |  |  |
| ASDR |  |  |  |  |  |  |  |
|  | both | East Asia (49.79) | North Africa and Middle East (63.66) | Central Asia (80.34) | Australasia (2.08) | High-income North America (4.32) | Western Europe (6.02) |
|  | female | East Asia (36.25) | North Africa and Middle East (56.29) | Central Asia (63.34) | Australasia (1.53) | High-income North America (3.21) | High-income Asia Pacific (4.38) |
|  | male | East Asia (66.86) | North Africa and Middle East (70.61) | Central Asia (103.28) | Australasia (2.69) | High-income North America (5.58) | Western Europe (7.84) |
| Age-standardized DALY rate |  |  |  |  |  |  |  |
|  | both | South Asia (1099.64) | North Africa and Middle East (1459.36) | Central Asia (1739.73) | Australasia (44.03) | High-income North America (104.83) | Western Europe (124.83) |
|  | female | South Asia (800.02) | North Africa and Middle East (1227.20) | Central Asia (1274.97) | Australasia (30.06) | High-income North America (75.78) | Western Europe (83.81) |
|  | male | South Asia (1401.50) | North Africa and Middle East (1675.78) | Central Asia (2315.35) | Australasia (59.04) | High-income North America (136.49) | Western Europe (169.26) |
| 1990-2019 increase times |  |  |  |  |  |  |  |
| Death (cases) |  |  |  |  |  |  |  |
|  | both | Central sub-Saharan Africa (4.15) | Eastern Sub-Saharan Africa (4.40) | South Asia (5.32) | Western Europe (0.38) | High-income North America (0.42) | Australasia (0.56) |
|  | female | Central sub-Saharan Africa (4.75) | Eastern Sub-Saharan Africa (4.85) | South Asia (6.35) | Western Europe (0.37) | High-income North America (0.40) | Australasia (0.56) |
|  | male | East Asia (4.06) | Eastern Sub-Saharan Africa (4.17) | South Asia (4.83) | Western Europe (0.39) | High-income North America (0.43) | Australasia (0.56) |
| DALY (cases) |  |  |  |  |  |  |  |
|  | both | Western sub-Saharan Africa (4.26) | Eastern Sub-Saharan Africa (4.31) | South Asia (4.97) | Western Europe (0.34) | High-income North America (0.42) | Australasia (0.49) |
|  | female | Eastern Sub-Saharan Africa (4.54) | Western sub-Saharan Africa (4.79) | South Asia (5.82) | Western Europe (0.32) | High-income North America (0.41) | Australasia (0.49) |
|  | male | Central sub-Saharan Africa (3.95) | Eastern Sub-Saharan Africa (4.20) | South Asia (4.59) | Western Europe (0.35) | High-income North America (0.43) | Australasia (0.49) |
| EAPC |  |  |  |  |  |  |  |
| Death |  |  |  |  |  |  |  |
|  | both | Western sub-Saharan Africa (2.43) | South Asia (2.77) | Eastern Sub-Saharan Africa (2.88) | High-income North America (-5.53) | Australasia (-5.45) | Western Europe (-5.40) |
|  | female | Western sub-Saharan Africa (2.68) | South Asia (2.98) | Eastern Sub-Saharan Africa (3.11) | Western Europe (-5.54) | Australasia (-5.50) | High-income North America (-5.50) |
|  | male | Western sub-Saharan Africa (2.29) | South Asia (2.77) | Eastern Sub-Saharan Africa (2.82) | High-income North America (-5.64) | Australasia (-5.49) | Western Europe (-5.41) |
| DALY |  |  |  |  |  |  |  |
|  | both | Western sub-Saharan Africa (2.42) | Eastern Sub-Saharan Africa (2.72) | South Asia (2.88) | Western Europe (-5.32) | Australasia (-5.31) | High-income North America (-5.18) |
|  | female | Western sub-Saharan Africa (2.72) | Eastern Sub-Saharan Africa (2.86) | South Asia (3.10) | Western Europe (-5.40) | Australasia (-5.31) | High-income North America (-5.04) |
|  | male | Western sub-Saharan Africa (2.31) | Eastern Sub-Saharan Africa (2.71) | South Asia (2.87) | Western Europe (-5.36) | Australasia (-5.34) | High-income North America (-5.33) |

**Supplementary Table 2.** The top three and the bottom three countries of global CVD burden due to ambient particulate matter pollution.

| Measure | sex | Top three countries | | | Bottom three countries | | |
| --- | --- | --- | --- | --- | --- | --- | --- |
| 2019 ASR (per 100,000 people) |  |  |  |  |  |  |  |
| ASDR |  |  |  |  |  |  |  |
|  | both | Iraq (98.04) | Egypt (125.55) | Uzbekistan (154.77) | Iceland (1.46) | Sweden (1.68) | Norway (1.91) |
|  | female | Qatar(102.44) | Uzbekistan(131.70) | Egypt (135.18) | Iceland (0.81) | Sweden (1.24) | Norway (1.41) |
|  | male | Egypt(124.18) | Mongolia(127.81) | Uzbekistan (184.22) | Sweden (2.17) | Iceland (2.18) | Norway (2.45) |
| Age-standardized DALY rate |  |  |  |  |  |  |  |
|  | both | Iraq(2198.11) | Egypt(2912.41) | Uzbekistan(2996.10) | Iceland(30.88) | Sweden(34.58) | Norway(41.25) |
|  | female | Saudi Arabia(1804.65) | Uzbekistan(2354.64) | Egypt(2794.24) | Iceland(15.87) | Sweden(23.84) | Finland(26.27) |
|  | male | Mongolia(2999.63) | Egypt(3090.34) | Uzbekistan(3774.02) | Sweden(45.74) | Iceland(46.49) | Norway(54.56) |
| 1990-2019 increase times |  |  |  |  |  |  |  |
| Death (cases) |  |  |  |  |  |  |  |
|  | both | Equatorial Guinea(9.86) | Timor-Leste(12.12) | Djibouti(12.95) | Norway(0.16) | Sweden(0.19) | Estonia(0.20) |
|  | female | Angola(11.45) | Djibouti(13.88) | Equatorial Guinea(15.06) | Norway(0.17) | Sweden(0.19) | Estonia(0.21) |
|  | male | Bhutan(9.62) | Djibouti(12.49) | Timor-Leste(12.63) | Norway(0.15) | Sweden(0.18) | Estonia(0.19) |
| DALY (cases) |  |  |  |  |  |  |  |
|  | both | Equatorial Guinea(9.28) | Timor-Leste(10.22) | Djibouti(12.21) | Norway(0.16) | Estonia(0.17) | Sweden(0.18) |
|  | female | Angola(11.01) | Djibouti(13.17) | Equatorial Guinea(14.05) | Norway(0.16) | Estonia(0.17) | Sweden(0.18) |
|  | male | Bhutan(8.08) | Timor-Leste(10.53) | Djibouti(11.77) | Norway(0.15) | Estonia(0.16) | Sweden(0.17) |
| EAPC |  |  |  |  |  |  |  |
| Death |  |  |  |  |  |  |  |
|  | both | Bhutan(5.66) | Timor-Leste(6.09) | Equatorial Guinea(6.10) | Norway(-7.93) | Estonia(-7.39) | Sweden(-7.10) |
|  | female | Bhutan(5.33) | Timor-Leste(5.65) | Equatorial Guinea(7.71) | Norway(-7.69) | Estonia(-7.67) | Bermuda(-7.14) |
|  | male | Mozambique(5.24) | Bhutan(5.69) | Timor-Leste(6.40) | Norway(-8.19) | Sweden(-7.37) | Estonia(-7.23) |
| DALY |  |  |  |  |  |  |  |
|  | both | Bhutan(5.41) | Equatorial Guinea(5.58) | Timor-Leste(6.06) | Norway(-7.81) | Estonia(-7.57) | Finland(-7.09) |
|  | female | Lesotho(5.20) | Timor-Leste(5.56) | Equatorial Guinea(7.35) | Estonia(-7.78) | Norway(-7.46) | Finland(-7.10) |
|  | male | Mozambique(5.51) | Bhutan(5.55) | Timor-Leste(6.40) | Norway(-8.08) | Estonia(-7.50) | Sweden(-7.29) |

**Supplementary Table 3.** The death of global CVD burden due to ambient particulate matter pollution, and its temporal trends from 1990 to 2019.

| Nation | Sex | Death Cases No. (95% UI) | | Change in absolute number (%) | ASDR per 100,000 No.(95% UI) | | 1990-2019 EAPC No. (95%CI) |
| --- | --- | --- | --- | --- | --- | --- | --- |
|  |  | 1990 | 2019 |  | 1990 | 2019 |  |
| Afghanistan | both | 1291.22(316.57,3419.55) | 4754.53(2095.59,9039.83) | 2.68 | 19.45(4.92,50.77) | 39.35(17.59,72.56) | 2.73(2.16,3.30) |
| Albania | both | 540.41(261.45,881.04) | 1245.44(873.51,1712.25) | 1.30 | 29.69(14.52,48.65) | 29.55(20.86,40.45) | 0.56(0.22,0.90) |
| Algeria | both | 7972.62(5103.90,11155.96) | 16863.68(11579.58,22690.67) | 1.12 | 83.08(53.50,115.06) | 59.86(41.61,80.09) | -1.48(-1.70,-1.27) |
| American Samoa | both | 2.59(0.83,6.63) | 4.41(1.69,8.57) | 0.70 | 11.67(3.67,30.20) | 9.56(3.72,18.59) | -0.95(-1.21,-0.69) |
| Andorra | both | 4.85(1.52,9.13) | 4.90(2.59,7.69) | 0.01 | 10.29(3.23,19.53) | 3.30(1.73,5.21) | -3.77(-3.93,-3.61) |
| Angola | both | 249.30(72.58,607.77) | 2272.48(1144.17,3855.55) | 8.12 | 7.10(2.18,16.94) | 23.34(11.53,39.34) | 4.19(3.92,4.45) |
| Antigua and Barbuda | both | 15.33(4.15,30.25) | 17.07(6.47,29.43) | 0.11 | 28.00(7.59,55.23) | 18.17(6.94,31.22) | -1.86(-2.16,-1.56) |
| Argentina | both | 7899.95(3080.36,14112.24) | 6166.08(3879.43,8730.23) | -0.22 | 25.49(9.93,45.50) | 11.36(7.15,16.09) | -2.93(-3.25,-2.61) |
| Armenia | both | 1495.07(728.87,2403.64) | 2210.04(1510.26,2939.22) | 0.48 | 63.80(30.88,102.41) | 54.89(37.55,73.05) | -0.80(-1.03,-0.58) |
| Australia | both | 1668.30(195.27,3906.12) | 928.43(239.74,1718.74) | -0.44 | 8.71(1.03,20.42) | 2.08(0.54,3.80) | -5.49(-5.81,-5.16) |
| Austria | both | 4024.24(2004.02,6270.38) | 1469.49(1077.31,1902.49) | -0.63 | 32.81(16.40,51.07) | 7.31(5.39,9.35) | -5.55(-5.73,-5.37) |
| Azerbaijan | both | 2320.27(1077.07,4045.45) | 6414.25(3929.74,9278.73) | 1.76 | 49.27(23.03,85.97) | 89.45(54.70,129.81) | 2.07(1.76,2.38) |
| Bahrain | both | 205.20(167.12,245.00) | 354.05(277.15,447.54) | 0.73 | 135.68(110.38,161.44) | 51.28(40.83,64.34) | -3.63(-3.92,-3.33) |
| Bangladesh | both | 6029.15(1863.78,13322.76) | 46206.63(28833.26,66486.25) | 6.66 | 13.82(4.46,30.32) | 37.61(23.57,53.94) | 3.90(3.64,4.17) |
| Barbados | both | 95.22(28.23,177.73) | 90.99(40.20,150.07) | -0.04 | 31.60(9.46,58.54) | 18.58(8.23,30.67) | -2.30(-2.68,-1.92) |
| Belarus | both | 8564.06(4479.68,13087.20) | 7582.03(5278.54,10446.90) | -0.11 | 68.26(35.44,104.17) | 47.03(32.73,64.69) | -1.37(-1.94,-0.79) |
| Belgium | both | 4435.12(2132.76,7071.04) | 1675.50(1241.86,2127.97) | -0.62 | 28.43(13.70,45.10) | 6.57(4.92,8.30) | -5.16(-5.36,-4.96) |
| Belize | both | 14.16(3.24,31.99) | 46.85(17.89,82.40) | 2.31 | 15.62(3.57,35.35) | 17.72(6.60,31.23) | -0.55(-1.19,0.09) |
| Benin | both | 158.62(53.44,357.94) | 692.58(316.98,1246.14) | 3.37 | 8.62(2.92,19.28) | 16.01(7.51,28.77) | 2.12(1.82,2.41) |
| Bermuda | both | 11.09(2.51,27.96) | 4.73(0.84,9.22) | -0.57 | 18.54(4.21,46.77) | 3.52(0.63,6.85) | -6.27(-6.77,-5.77) |
| Bhutan | both | 14.39(3.90,34.56) | 128.71(77.05,188.51) | 7.95 | 6.50(1.78,15.66) | 24.50(14.88,35.79) | 5.66(5.29,6.03) |
| Bolivia (Plurinational State of) | both | 849.10(309.12,1586.19) | 1817.11(1041.43,2746.26) | 1.14 | 28.64(10.58,53.17) | 22.46(12.99,33.73) | -1.03(-1.50,-0.57) |
| Bosnia and Herzegovina | both | 1472.06(736.90,2317.81) | 2574.31(1928.55,3282.86) | 0.75 | 42.29(21.03,67.12) | 44.93(33.74,57.13) | 0.24(-0.12,0.61) |
| Botswana | both | 87.05(41.36,152.04) | 429.05(267.35,640.61) | 3.93 | 16.92(8.19,29.38) | 35.53(22.32,51.81) | 2.35(1.74,2.97) |
| Brazil | both | 18185.39(8276.62,31933.01) | 23866.10(16740.24,31519.47) | 0.31 | 21.28(9.66,37.72) | 10.22(7.15,13.48) | -2.60(-2.83,-2.36) |
| Brunei Darussalam | both | 15.26(5.72,30.57) | 19.50(5.84,34.75) | 0.28 | 17.31(6.50,34.73) | 7.85(2.25,14.06) | -2.43(-3.14,-1.72) |
| Bulgaria | both | 9943.64(5554.50,14791.24) | 7558.98(5865.30,9515.78) | -0.24 | 90.47(50.57,135.11) | 51.66(39.93,65.30) | -2.74(-3.13,-2.34) |
| Burkina Faso | both | 189.51(49.25,476.09) | 876.42(306.73,1850.06) | 3.62 | 5.04(1.36,12.75) | 10.84(3.83,22.49) | 3.33(3.07,3.58) |
| Burundi | both | 187.77(52.49,489.41) | 341.51(112.70,794.56) | 0.82 | 8.47(2.44,21.77) | 8.49(2.91,19.28) | -0.21(-0.51,0.09) |
| Cambodia | both | 350.02(101.41,864.77) | 1799.00(862.19,3045.62) | 4.14 | 8.61(2.52,21.02) | 16.87(8.17,28.22) | 2.66(2.43,2.88) |
| Cameroon | both | 656.93(288.94,1185.33) | 3704.93(2124.01,5417.33) | 4.64 | 17.07(7.69,29.65) | 34.93(20.60,50.67) | 2.58(2.40,2.76) |
| Canada | both | 3805.50(961.63,7659.78) | 1873.20(880.41,3022.31) | -0.51 | 11.89(3.01,23.98) | 2.61(1.23,4.19) | -5.88(-6.19,-5.56) |
| Cabo Verde | both | 26.43(11.24,48.83) | 183.32(126.15,241.19) | 5.94 | 11.24(4.72,20.80) | 44.23(30.69,58.13) | 4.15(3.59,4.71) |
| Central African Republic | both | 113.68(29.18,279.02) | 296.03(90.63,676.80) | 1.60 | 10.55(2.69,25.47) | 14.95(4.73,33.60) | 1.25(1.01,1.48) |
| Chad | both | 151.00(38.69,414.99) | 580.93(209.50,1266.57) | 2.85 | 5.89(1.52,15.93) | 11.68(4.28,25.36) | 2.67(2.52,2.82) |
| Chile | both | 2289.84(1128.84,3782.61) | 3249.34(2603.63,3925.01) | 0.42 | 24.92(12.17,41.27) | 13.60(10.90,16.42) | -1.95(-2.09,-1.82) |
| China | both | 223214.60(106294.37,375432.65) | 915668.37(749376.62,1099607.64) | 3.10 | 31.23(15.06,52.03) | 50.86(41.70,60.76) | 2.11(1.72,2.51) |
| Colombia | both | 4033.15(1918.62,6975.18) | 7526.34(5086.67,10590.42) | 0.87 | 24.63(11.68,42.74) | 14.00(9.43,19.74) | -2.24(-2.39,-2.09) |
| Comoros | both | 9.95(3.30,23.12) | 42.83(20.46,75.23) | 3.30 | 4.85(1.63,11.10) | 9.44(4.50,16.45) | 2.13(1.98,2.27) |
| Congo | both | 206.81(75.82,426.30) | 932.95(501.48,1510.98) | 3.51 | 21.06(7.81,43.86) | 40.80(21.76,65.95) | 2.19(1.91,2.47) |
| Costa Rica | both | 235.36(109.40,412.67) | 578.63(397.35,796.21) | 1.46 | 14.00(6.47,24.68) | 11.28(7.73,15.50) | -1.04(-1.28,-0.79) |
| Côte d'Ivoire | both | 472.29(164.09,1053.98) | 2131.09(1036.19,3620.34) | 3.51 | 13.93(5.03,30.55) | 23.01(11.28,38.72) | 1.70(1.38,2.01) |
| Croatia | both | 3718.37(1987.49,5574.59) | 2247.28(1689.47,2889.92) | -0.40 | 62.78(33.24,94.43) | 24.28(18.12,31.21) | -3.39(-3.61,-3.17) |
| Cuba | both | 3321.29(1261.26,5977.28) | 3614.56(1803.04,5944.04) | 0.09 | 32.87(12.44,59.28) | 18.56(9.25,30.45) | -2.17(-2.43,-1.91) |
| Cyprus | both | 246.08(103.08,412.76) | 233.29(176.34,306.51) | -0.05 | 36.28(15.18,61.26) | 13.07(9.86,17.07) | -4.12(-4.41,-3.83) |
| Czechia | both | 10771.98(5771.34,15954.44) | 4319.15(3288.81,5586.95) | -0.60 | 79.51(42.44,118.31) | 19.82(15.11,25.43) | -4.93(-5.04,-4.81) |
| Democratic Republic of the Congo | both | 1288.51(368.14,3105.43) | 4182.53(1698.30,8193.57) | 2.25 | 9.51(2.82,22.47) | 13.34(5.57,25.89) | 0.76(0.12,1.42) |
| Denmark | both | 2575.01(1067.96,4323.93) | 570.59(350.72,808.24) | -0.78 | 30.72(12.70,51.58) | 4.68(2.90,6.60) | -6.81(-7.06,-6.55) |
| Djibouti | both | 16.12(5.27,35.48) | 208.79(103.15,327.47) | 11.95 | 13.19(4.54,29.13) | 40.72(20.17,63.50) | 4.49(4.00,4.97) |
| Dominica | both | 14.88(5.37,29.43) | 17.33(7.28,30.12) | 0.16 | 20.21(7.27,40.35) | 19.08(7.98,33.23) | -0.40(-0.59,-0.21) |
| Dominican Republic | both | 375.80(115.17,862.97) | 2771.73(1258.71,5040.89) | 6.38 | 10.59(3.23,24.52) | 30.36(13.75,55.57) | 4.80(4.46,5.14) |
| Ecuador | both | 872.31(398.62,1524.38) | 2233.20(1434.46,3131.45) | 1.56 | 16.99(7.77,29.94) | 15.65(10.11,21.99) | 0.15(-0.22,0.51) |
| Egypt | both | 33460.12(26315.61,39771.82) | 71756.87(52155.86,93005.82) | 1.14 | 125.80(98.84,149.77) | 125.55(92.42,160.73) | 0.07(-0.07,0.22) |
| El Salvador | both | 367.60(138.59,706.57) | 1047.68(643.09,1620.99) | 1.85 | 12.51(4.77,24.24) | 17.04(10.39,26.52) | 1.11(0.87,1.35) |
| Equatorial Guinea | both | 16.55(4.24,45.41) | 163.19(91.91,252.83) | 8.86 | 9.06(2.36,24.36) | 40.12(22.82,60.51) | 6.10(5.78,6.42) |
| Eritrea | both | 84.93(24.03,204.98) | 521.23(217.55,968.19) | 5.14 | 9.02(2.55,21.68) | 21.68(9.30,39.81) | 2.77(2.50,3.04) |
| Estonia | both | 618.54(246.20,1146.02) | 124.43(46.15,224.15) | -0.80 | 30.95(12.32,57.54) | 4.29(1.56,7.67) | -7.39(-8.12,-6.65) |
| Ethiopia | both | 820.92(230.94,2169.06) | 3138.87(1491.94,5633.36) | 2.82 | 4.43(1.32,11.32) | 8.53(4.09,15.17) | 2.44(2.13,2.74) |
| Micronesia (Federated States of) | both | 7.68(1.64,21.20) | 20.48(6.17,47.31) | 1.67 | 16.43(3.45,45.10) | 29.36(8.92,68.28) | 1.97(1.78,2.15) |
| Fiji | both | 54.86(13.20,148.31) | 177.56(54.98,385.90) | 2.24 | 15.06(3.60,40.35) | 25.05(7.65,54.72) | 1.51(0.94,2.08) |
| Finland | both | 1061.60(126.21,2303.55) | 287.80(58.44,590.67) | -0.73 | 14.87(1.78,32.51) | 2.13(0.43,4.36) | -6.81(-7.33,-6.29) |
| France | both | 14567.27(6518.49,24163.09) | 6417.03(4622.99,8397.68) | -0.56 | 16.84(7.57,27.79) | 4.10(2.98,5.30) | -4.84(-5.05,-4.62) |
| Gabon | both | 117.15(49.75,215.13) | 417.97(254.41,602.58) | 2.57 | 22.43(9.46,41.10) | 44.84(27.44,64.07) | 2.30(1.89,2.71) |
| Georgia | both | 3566.97(1841.18,6093.49) | 2552.54(1759.48,3455.97) | -0.28 | 63.05(32.17,107.83) | 41.21(28.37,55.60) | -1.77(-1.96,-1.57) |
| Germany | both | 48116.53(22814.33,76316.97) | 15999.86(11711.07,20661.03) | -0.67 | 36.83(17.51,58.45) | 7.54(5.57,9.61) | -5.50(-5.74,-5.26) |
| Ghana | both | 1065.51(478.47,1900.74) | 6127.80(3747.22,8785.90) | 4.75 | 19.73(9.02,34.20) | 43.32(26.92,61.56) | 3.24(3.04,3.44) |
| Greece | both | 5232.17(2478.42,8435.17) | 3687.97(2792.78,4591.59) | -0.30 | 35.15(16.78,56.91) | 13.80(10.59,17.00) | -3.53(-3.91,-3.15) |
| Greenland | both | 4.06(0.90,10.80) | 3.14(0.28,8.87) | -0.23 | 13.08(2.95,35.25) | 4.97(0.46,14.18) | -4.13(-4.53,-3.73) |
| Grenada | both | 23.00(6.27,50.67) | 29.39(11.60,49.92) | 0.28 | 30.68(8.48,67.22) | 28.17(11.02,48.03) | -0.87(-1.10,-0.63) |
| Guam | both | 10.93(1.95,28.67) | 21.52(8.84,36.61) | 0.97 | 16.18(2.86,43.84) | 11.49(4.70,19.67) | -1.07(-1.87,-0.27) |
| Guatemala | both | 362.39(124.90,768.50) | 1512.64(849.42,2287.60) | 3.17 | 11.51(4.05,24.35) | 14.76(8.40,22.05) | 0.24(-0.23,0.71) |
| Guinea | both | 204.67(62.45,499.65) | 709.89(303.53,1400.07) | 2.47 | 6.87(2.13,16.64) | 13.99(5.98,27.36) | 2.99(2.84,3.14) |
| Guinea-Bissau | both | 43.08(12.38,106.65) | 137.59(60.01,257.36) | 2.19 | 11.66(3.46,28.28) | 20.90(9.41,38.93) | 2.29(2.19,2.39) |
| Guyana | both | 226.13(59.61,460.69) | 276.99(118.88,503.14) | 0.22 | 61.18(15.66,125.52) | 46.57(19.74,84.87) | -0.81(-0.98,-0.64) |
| Haiti | both | 311.06(84.50,768.38) | 1030.65(370.73,2150.25) | 2.31 | 10.32(2.78,25.60) | 16.12(5.93,33.00) | 1.83(1.70,1.96) |
| Honduras | both | 162.96(53.04,356.95) | 1108.56(600.13,1750.96) | 5.80 | 8.27(2.73,18.10) | 20.19(10.97,31.65) | 3.51(3.13,3.89) |
| Hungary | both | 8905.83(4826.15,13471.10) | 4838.12(3731.89,6150.56) | -0.46 | 63.24(34.31,95.94) | 24.29(18.71,30.89) | -3.64(-3.79,-3.48) |
| Iceland | both | 21.68(1.92,53.77) | 8.78(1.81,18.40) | -0.59 | 7.40(0.66,18.45) | 1.46(0.30,3.05) | -5.76(-6.04,-5.48) |
| India | both | 97450.38(47068.50,163566.53) | 492784.76(379246.89,605597.53) | 4.06 | 24.22(11.89,40.28) | 45.32(34.91,55.76) | 2.44(2.27,2.60) |
| Indonesia | both | 20272.26(8667.05,36592.74) | 75020.56(53653.58,97287.35) | 2.70 | 21.94(9.44,39.76) | 38.43(27.38,50.02) | 1.86(1.63,2.10) |
| Iran (Islamic Republic of) | both | 16199.49(13136.58,19149.19) | 30856.60(26577.85,35109.01) | 0.90 | 74.13(60.06,87.70) | 46.16(39.57,52.59) | -2.05(-2.34,-1.76) |
| Iraq | both | 7018.56(4717.35,9281.30) | 19959.24(15046.50,25201.66) | 1.84 | 95.42(64.35,125.49) | 98.04(75.05,121.03) | 0.08(-0.16,0.33) |
| Ireland | both | 995.64(297.83,1846.73) | 282.33(142.22,433.51) | -0.72 | 24.67(7.41,45.57) | 3.69(1.88,5.66) | -6.94(-7.18,-6.70) |
| Israel | both | 1578.32(847.82,2404.02) | 1064.43(859.86,1269.23) | -0.33 | 33.24(17.84,50.40) | 8.70(7.04,10.37) | -5.25(-5.47,-5.03) |
| Italy | both | 26769.28(14649.62,39814.27) | 13692.92(10630.83,16885.00) | -0.49 | 30.20(16.58,44.97) | 8.08(6.35,9.85) | -4.73(-4.85,-4.61) |
| Jamaica | both | 222.99(98.57,419.24) | 481.48(298.31,704.40) | 1.16 | 12.33(5.43,23.06) | 15.74(9.75,22.95) | 0.81(0.50,1.11) |
| Japan | both | 19793.33(6561.50,36460.99) | 19841.70(13338.57,27525.68) | 0.00 | 12.44(4.12,23.11) | 5.01(3.45,6.83) | -3.28(-3.63,-2.93) |
| Jordan | both | 773.78(614.48,945.92) | 2024.83(1608.47,2536.88) | 1.62 | 67.40(54.14,82.49) | 36.66(29.35,45.70) | -2.59(-2.88,-2.30) |
| Kazakhstan | both | 5815.16(2862.70,9962.48) | 7660.39(5053.06,10672.83) | 0.32 | 49.77(24.29,85.64) | 51.07(33.86,70.76) | -0.57(-1.07,-0.07) |
| Kenya | both | 391.96(170.42,756.95) | 2406.61(1363.16,3836.50) | 5.14 | 5.33(2.34,10.19) | 12.40(6.93,19.95) | 3.63(3.28,3.99) |
| Kiribati | both | 5.24(1.27,14.14) | 12.10(3.57,29.96) | 1.31 | 13.18(3.27,35.88) | 16.55(4.96,41.13) | -0.10(-0.39,0.20) |
| Kuwait | both | 397.41(349.26,444.43) | 1112.06(907.86,1365.80) | 1.80 | 66.94(57.92,75.78) | 42.95(35.04,52.58) | -1.03(-1.50,-0.57) |
| Kyrgyzstan | both | 1022.85(438.62,1862.43) | 2094.32(1284.42,3051.41) | 1.05 | 34.82(14.84,63.62) | 51.77(31.79,76.32) | 1.21(0.76,1.67) |
| Lao People's Democratic Republic | both | 193.83(54.05,456.36) | 793.74(397.41,1297.39) | 3.09 | 10.17(2.91,24.18) | 19.97(10.07,32.30) | 2.29(2.02,2.55) |
| Latvia | both | 2324.24(1175.24,3606.36) | 956.43(661.73,1335.17) | -0.59 | 65.70(33.44,101.51) | 22.17(15.26,30.84) | -3.98(-4.53,-3.43) |
| Lebanon | both | 1218.03(859.23,1611.12) | 2517.51(1747.41,3228.67) | 1.07 | 58.68(41.57,76.54) | 48.63(33.76,62.04) | -0.43(-0.66,-0.21) |
| Lesotho | both | 97.75(43.28,186.20) | 335.66(179.37,535.89) | 2.43 | 10.88(4.90,20.50) | 29.29(15.56,46.67) | 4.18(3.86,4.50) |
| Liberia | both | 93.52(32.21,210.01) | 267.58(122.87,501.00) | 1.86 | 9.43(3.25,20.81) | 14.79(6.92,27.27) | 2.65(2.17,3.13) |
| Libya | both | 792.58(492.79,1120.65) | 2586.11(1813.28,3617.98) | 2.26 | 44.66(27.72,62.94) | 52.85(37.07,73.24) | 0.35(0.10,0.59) |
| Lithuania | both | 2108.86(993.24,3479.89) | 1084.73(712.60,1553.38) | -0.49 | 47.19(22.11,77.80) | 17.41(11.42,24.92) | -3.26(-3.81,-2.71) |
| Luxembourg | both | 150.97(60.21,260.47) | 48.92(31.16,67.39) | -0.68 | 27.73(11.14,47.83) | 4.53(2.92,6.26) | -6.27(-6.54,-6.00) |
| North Macedonia | both | 1544.49(921.37,2161.67) | 2182.17(1676.20,2779.13) | 0.41 | 93.22(54.71,131.07) | 78.70(61.16,98.80) | -0.81(-1.16,-0.45) |
| Madagascar | both | 259.01(92.59,585.54) | 1081.44(460.56,2150.61) | 3.18 | 5.33(1.96,11.88) | 10.84(4.73,21.01) | 2.76(2.59,2.92) |
| Malawi | both | 135.21(39.16,353.93) | 475.58(195.58,941.21) | 2.52 | 3.98(1.21,10.30) | 7.18(3.03,14.11) | 2.59(2.36,2.81) |
| Malaysia | both | 4777.25(2236.48,7349.69) | 6889.95(4761.10,9552.81) | 0.44 | 55.07(25.68,85.21) | 27.34(18.88,37.63) | -2.46(-2.59,-2.33) |
| Maldives | both | 12.98(4.08,29.09) | 32.31(20.39,46.74) | 1.49 | 16.39(5.13,37.01) | 11.31(7.12,16.55) | -2.17(-2.51,-1.82) |
| Mali | both | 233.21(60.79,603.90) | 806.06(278.33,1663.67) | 2.46 | 6.84(1.85,17.47) | 11.15(3.87,22.53) | 2.07(1.90,2.24) |
| Malta | both | 128.02(53.27,217.78) | 92.41(66.90,119.07) | -0.28 | 30.90(12.88,52.54) | 9.53(6.94,12.29) | -3.98(-4.29,-3.67) |
| Marshall Islands | both | 2.07(0.47,5.50) | 8.12(2.74,16.93) | 2.93 | 12.61(2.88,33.40) | 23.33(7.91,49.19) | 2.04(1.72,2.36) |
| Mauritania | both | 195.43(79.55,366.24) | 578.18(326.02,835.37) | 1.96 | 21.29(8.83,39.95) | 30.94(17.60,44.37) | 1.17(0.93,1.40) |
| Mauritius | both | 277.08(143.44,428.49) | 287.55(139.75,449.78) | 0.04 | 38.87(19.92,60.69) | 17.32(8.49,27.07) | -3.85(-4.40,-3.29) |
| Mexico | both | 8510.60(4453.19,13389.82) | 17556.51(13039.02,22331.13) | 1.06 | 21.98(11.41,34.71) | 15.68(11.63,19.87) | -1.55(-1.84,-1.26) |
| Republic of Moldova | both | 2125.89(991.44,3561.27) | 1821.14(1044.18,2674.42) | -0.14 | 55.01(25.48,92.95) | 31.44(18.10,46.07) | -1.86(-2.43,-1.28) |
| Mongolia | both | 428.52(188.31,767.03) | 1900.49(1288.41,2622.52) | 3.43 | 43.96(19.16,78.75) | 91.41(62.98,123.94) | 2.30(1.91,2.68) |
| Montenegro | both | 348.20(197.66,508.14) | 449.55(343.41,563.81) | 0.29 | 59.40(33.79,86.81) | 47.26(35.95,59.10) | -0.64(-0.88,-0.40) |
| Morocco | both | 4801.88(2561.53,7642.38) | 21627.67(15992.53,27465.99) | 3.50 | 38.26(20.65,60.62) | 77.35(58.13,96.63) | 2.31(2.15,2.48) |
| Mozambique | both | 144.90(36.43,402.42) | 771.72(324.25,1604.35) | 4.33 | 2.74(0.72,7.47) | 7.65(3.24,15.62) | 4.69(4.29,5.10) |
| Myanmar | both | 4095.05(1378.49,8949.26) | 13910.82(8558.74,20168.69) | 2.40 | 18.77(6.55,40.74) | 32.71(20.38,47.02) | 2.28(1.99,2.56) |
| Namibia | both | 118.33(56.20,206.69) | 375.42(222.60,552.05) | 2.17 | 17.92(8.64,31.14) | 29.44(17.40,42.73) | 1.71(1.41,2.02) |
| Nepal | both | 903.55(249.98,2074.81) | 6612.99(3838.28,9559.93) | 6.32 | 10.51(2.99,24.16) | 32.22(18.99,46.40) | 4.49(4.17,4.81) |
| Netherlands | both | 5411.51(2571.07,8736.00) | 2003.56(1473.90,2564.88) | -0.63 | 26.80(12.73,43.25) | 5.60(4.14,7.12) | -5.89(-6.14,-5.64) |
| New Zealand | both | 312.54(21.43,804.10) | 172.12(31.42,349.15) | -0.45 | 8.04(0.55,20.75) | 2.11(0.39,4.23) | -5.29(-5.55,-5.02) |
| Nicaragua | both | 82.82(28.49,174.22) | 584.11(320.40,926.93) | 6.05 | 5.89(2.03,12.34) | 15.32(8.38,24.35) | 3.18(2.70,3.66) |
| Niger | both | 168.72(37.02,499.17) | 641.24(170.35,1587.44) | 2.80 | 7.25(1.62,21.38) | 9.98(2.75,24.48) | 1.21(1.05,1.37) |
| Nigeria | both | 5510.41(2407.88,11391.27) | 20534.02(12553.59,30100.69) | 2.73 | 14.58(6.44,29.33) | 28.24(17.45,40.67) | 2.34(2.08,2.59) |
| Democratic People's Republic of Korea | both | 3165.30(1253.83,5898.17) | 13269.93(8297.17,19077.49) | 3.19 | 22.24(8.96,41.54) | 42.89(26.94,61.24) | 2.55(2.41,2.68) |
| Northern Mariana Islands | both | 3.65(1.13,8.79) | 6.46(3.40,10.46) | 0.77 | 19.16(6.09,46.65) | 13.12(6.93,21.10) | -1.16(-1.77,-0.55) |
| Norway | both | 1243.57(329.31,2393.84) | 200.29(73.88,349.93) | -0.84 | 17.53(4.65,33.79) | 1.91(0.71,3.35) | -7.93(-8.24,-7.62) |
| Oman | both | 487.51(265.24,772.59) | 1184.78(915.77,1483.73) | 1.43 | 83.50(45.60,131.13) | 94.86(73.57,117.78) | 0.14(-0.34,0.63) |
| Pakistan | both | 8462.77(2887.55,17728.10) | 54176.92(35906.00,74424.29) | 5.40 | 15.61(5.39,32.51) | 51.19(34.14,69.94) | 4.49(4.20,4.78) |
| Palestine | both | 372.71(182.84,601.46) | 1216.20(905.38,1519.88) | 2.26 | 46.86(23.18,74.98) | 61.29(45.72,76.49) | 0.35(-0.10,0.79) |
| Panama | both | 203.98(91.82,369.89) | 350.54(205.41,520.77) | 0.72 | 14.19(6.35,25.90) | 8.36(4.88,12.43) | -1.38(-1.62,-1.14) |
| Papua New Guinea | both | 107.70(19.67,338.65) | 485.05(111.28,1244.50) | 3.50 | 6.12(1.13,19.50) | 10.44(2.47,26.28) | 1.91(1.73,2.09) |
| Paraguay | both | 229.45(99.15,420.70) | 614.37(376.57,952.12) | 1.68 | 10.73(4.65,19.73) | 11.34(6.92,17.63) | 0.06(-0.16,0.29) |
| Peru | both | 2245.38(1053.26,3752.03) | 3909.18(2570.65,5562.82) | 0.74 | 19.60(9.15,32.73) | 12.00(7.89,17.13) | -1.67(-2.15,-1.19) |
| Philippines | both | 3462.11(1698.51,5836.15) | 19943.72(13898.88,27021.66) | 4.76 | 14.52(7.14,24.33) | 26.36(18.46,35.57) | 2.40(1.56,3.24) |
| Poland | both | 29739.99(17098.47,42816.89) | 18731.91(14908.28,23122.36) | -0.37 | 71.01(40.80,102.32) | 25.91(20.71,31.92) | -3.95(-4.13,-3.78) |
| Portugal | both | 2939.92(961.48,5573.40) | 1079.67(633.45,1566.15) | -0.63 | 22.05(7.14,41.70) | 4.03(2.36,5.81) | -6.40(-6.78,-6.02) |
| Puerto Rico | both | 263.07(1.86,868.95) | 180.04(38.18,336.25) | -0.32 | 7.41(0.05,24.41) | 2.45(0.51,4.58) | -4.64(-5.25,-4.03) |
| Qatar | both | 118.92(95.51,146.93) | 366.08(270.69,485.57) | 2.08 | 136.75(110.51,164.73) | 76.20(60.10,95.67) | -2.14(-2.42,-1.87) |
| Romania | both | 13947.54(7173.82,21711.15) | 11758.29(9067.10,14833.24) | -0.16 | 55.44(28.65,86.48) | 30.35(23.17,38.50) | -2.76(-3.14,-2.38) |
| Russian Federation | both | 101524.77(43891.93,168184.37) | 63389.25(36686.87,91974.56) | -0.38 | 60.77(26.12,101.04) | 27.11(15.67,39.14) | -3.06(-3.72,-2.40) |
| Rwanda | both | 292.82(87.74,660.26) | 688.70(283.19,1307.58) | 1.35 | 10.94(3.30,24.42) | 13.40(5.61,24.76) | 0.22(-0.17,0.61) |
| Saint Lucia | both | 23.21(7.97,47.87) | 42.64(18.64,72.74) | 0.84 | 28.24(9.56,58.27) | 20.45(8.84,34.96) | -1.89(-2.30,-1.48) |
| Saint Vincent and the Grenadines | both | 18.31(5.34,40.30) | 36.72(14.72,64.14) | 1.01 | 26.28(7.64,57.98) | 28.62(11.41,49.95) | -0.10(-0.39,0.18) |
| Samoa | both | 14.27(3.50,36.59) | 26.60(7.52,59.68) | 0.86 | 17.03(4.12,43.88) | 18.74(5.31,42.05) | 0.09(-0.02,0.20) |
| Sao Tome and Principe | both | 4.85(1.89,10.04) | 25.49(13.20,42.20) | 4.25 | 8.50(3.41,17.35) | 27.31(14.21,44.98) | 4.58(4.33,4.84) |
| Saudi Arabia | both | 2998.44(1660.66,4700.40) | 14565.67(11335.56,18389.71) | 3.86 | 56.44(32.02,87.52) | 86.52(69.65,105.35) | 1.02(0.38,1.66) |
| Senegal | both | 348.41(120.37,775.77) | 1322.82(636.87,2309.24) | 2.80 | 12.18(4.30,26.39) | 19.80(9.69,34.03) | 1.52(1.33,1.70) |
| Serbia | both | 7472.02(4230.53,10824.44) | 8013.77(6241.12,10019.63) | 0.07 | 74.41(41.64,107.84) | 52.64(41.19,65.38) | -1.45(-1.84,-1.06) |
| Seychelles | both | 14.94(8.19,23.57) | 20.65(10.86,31.47) | 0.38 | 26.66(14.58,42.11) | 19.64(10.24,30.18) | -1.47(-1.63,-1.30) |
| Sierra Leone | both | 162.52(52.42,387.33) | 491.58(202.52,978.48) | 2.02 | 9.25(3.00,21.98) | 15.12(6.34,29.87) | 2.25(1.95,2.54) |
| Singapore | both | 692.68(241.43,1208.51) | 680.14(438.52,918.36) | -0.02 | 33.94(11.71,59.64) | 9.02(5.84,12.20) | -4.82(-5.16,-4.47) |
| Slovakia | both | 4615.32(2533.78,6754.42) | 2706.71(1997.25,3497.73) | -0.41 | 79.34(43.18,116.75) | 29.76(21.98,38.55) | -3.51(-3.72,-3.30) |
| Slovenia | both | 944.21(494.12,1536.09) | 490.72(353.60,671.45) | -0.48 | 39.54(20.76,64.05) | 10.30(7.43,14.02) | -5.27(-5.48,-5.07) |
| Solomon Islands | both | 12.65(2.26,40.57) | 69.56(18.36,183.12) | 4.50 | 9.16(1.63,28.89) | 21.97(5.77,57.17) | 2.88(2.74,3.02) |
| Somalia | both | 65.16(13.02,201.93) | 201.68(44.07,619.79) | 2.10 | 2.85(0.59,8.50) | 3.45(0.80,10.17) | 1.17(0.88,1.45) |
| South Africa | both | 4594.19(3435.68,5846.66) | 10563.38(8400.17,12871.89) | 1.30 | 22.36(16.61,28.66) | 25.76(20.34,31.44) | 0.60(0.12,1.09) |
| Republic of Korea | both | 14404.37(7954.18,21508.87) | 10686.73(8513.77,13279.72) | -0.26 | 57.03(31.09,86.21) | 12.60(10.00,15.61) | -5.63(-5.98,-5.28) |
| South Sudan | both | 164.66(54.16,373.80) | 349.67(139.25,647.34) | 1.12 | 7.58(2.53,16.90) | 10.60(4.31,19.42) | 1.51(1.36,1.67) |
| Spain | both | 8795.67(3362.59,15650.47) | 4211.64(2820.43,5753.02) | -0.52 | 16.48(6.37,29.30) | 3.86(2.60,5.22) | -5.09(-5.38,-4.79) |
| Sri Lanka | both | 1961.37(831.52,3538.15) | 4417.77(2750.07,6540.08) | 1.25 | 20.64(8.78,37.53) | 19.02(11.92,27.97) | 0.09(-0.40,0.58) |
| Sudan | both | 1937.16(670.44,4165.63) | 11815.33(7062.73,18020.17) | 5.10 | 22.43(7.94,47.94) | 68.78(41.72,102.77) | 4.16(4.04,4.29) |
| Suriname | both | 90.55(31.84,170.59) | 172.57(82.27,286.29) | 0.91 | 35.84(12.65,67.53) | 29.36(14.00,48.78) | -0.87(-1.26,-0.48) |
| Eswatini | both | 35.94(16.62,61.28) | 141.96(78.96,216.45) | 2.95 | 13.77(6.55,23.40) | 27.67(15.29,42.27) | 2.64(2.25,3.03) |
| Sweden | both | 2117.17(371.51,4424.80) | 398.88(107.78,802.31) | -0.81 | 13.19(2.32,27.47) | 1.68(0.45,3.37) | -7.10(-7.50,-6.70) |
| Switzerland | both | 2362.79(1013.03,3933.68) | 777.25(513.49,1082.64) | -0.67 | 21.51(9.21,35.65) | 3.84(2.54,5.30) | -6.00(-6.19,-5.82) |
| Syrian Arab Republic | both | 4693.27(3312.66,6230.80) | 9000.11(6475.27,12207.43) | 0.92 | 93.72(66.16,123.93) | 84.50(62.00,112.80) | -0.75(-0.97,-0.54) |
| Taiwan (Province of China) | both | 3947.84(2141.47,6227.87) | 4736.02(3635.92,6156.01) | 0.20 | 27.92(15.02,44.27) | 12.06(9.27,15.74) | -3.03(-3.22,-2.84) |
| Tajikistan | both | 811.20(278.75,1648.44) | 3478.12(1844.30,5422.41) | 3.29 | 29.94(10.30,60.99) | 93.44(48.85,147.34) | 4.17(3.94,4.40) |
| United Republic of Tanzania | both | 429.35(152.92,954.60) | 2238.21(1053.90,3997.35) | 4.21 | 4.38(1.57,9.71) | 10.26(4.92,17.92) | 3.39(3.21,3.56) |
| Thailand | both | 7889.44(3773.66,12808.16) | 17431.86(12391.20,23938.02) | 1.21 | 24.39(11.70,39.44) | 17.60(12.57,24.10) | -1.70(-2.05,-1.36) |
| Bahamas | both | 38.46(9.91,74.33) | 63.23(18.27,119.89) | 0.64 | 25.81(6.56,50.14) | 16.79(4.80,32.07) | -1.77(-1.99,-1.56) |
| Gambia | both | 28.35(8.73,67.10) | 201.41(100.15,346.53) | 6.10 | 9.36(3.02,21.60) | 23.08(11.46,39.79) | 3.26(3.09,3.43) |
| Timor-Leste | both | 10.05(2.40,27.63) | 121.85(53.12,219.71) | 11.12 | 4.23(1.06,11.30) | 16.64(7.32,30.18) | 6.09(5.44,6.74) |
| Togo | both | 127.43(47.34,259.84) | 666.94(320.85,1139.28) | 4.23 | 11.73(4.44,23.34) | 20.64(10.23,35.20) | 1.97(1.71,2.22) |
| Tonga | both | 4.56(1.21,11.55) | 10.07(3.18,22.08) | 1.21 | 8.71(2.31,22.11) | 12.85(4.06,28.28) | 1.15(0.87,1.42) |
| Trinidad and Tobago | both | 393.85(79.82,790.30) | 490.60(164.89,892.72) | 0.25 | 49.20(9.76,99.72) | 27.11(9.18,49.34) | -2.77(-3.13,-2.41) |
| Tunisia | both | 2240.76(1447.16,3041.96) | 5724.62(3875.46,7973.85) | 1.55 | 50.87(32.81,68.64) | 49.04(33.15,67.89) | -0.34(-0.69,0.01) |
| Turkey | both | 15006.36(11352.26,18480.26) | 24729.00(18737.44,31883.08) | 0.65 | 43.79(33.13,53.94) | 29.36(22.24,37.83) | -1.33(-1.57,-1.10) |
| Turkmenistan | both | 1436.67(599.85,2432.51) | 3061.98(1839.53,4526.90) | 1.13 | 82.53(34.51,139.95) | 85.77(51.08,127.41) | -0.58(-0.96,-0.21) |
| Uganda | both | 252.84(73.56,619.56) | 1465.07(698.75,2602.49) | 4.79 | 4.43(1.33,10.81) | 11.75(5.65,20.71) | 3.86(3.61,4.10) |
| Ukraine | both | 45381.86(21251.65,72072.17) | 39108.29(24189.99,56599.48) | -0.14 | 66.92(31.44,106.85) | 51.12(31.71,73.85) | -1.36(-1.79,-0.93) |
| United Arab Emirates | both | 412.30(310.33,563.22) | 2408.47(1612.65,3522.63) | 4.84 | 113.37(87.59,151.33) | 63.38(44.66,87.23) | -2.23(-2.72,-1.75) |
| United Kingdom | both | 27856.40(11442.29,46497.42) | 7109.62(4737.12,9734.41) | -0.74 | 30.29(12.55,50.44) | 5.38(3.60,7.31) | -6.51(-6.73,-6.29) |
| United States of America | both | 62367.15(24038.55,112067.57) | 25729.53(13692.32,39198.40) | -0.59 | 19.20(7.46,34.39) | 4.53(2.41,6.87) | -5.48(-5.78,-5.17) |
| Uruguay | both | 605.04(194.60,1256.91) | 388.18(193.22,620.28) | -0.36 | 15.76(5.10,32.54) | 6.80(3.42,10.86) | -3.16(-3.36,-2.95) |
| Uzbekistan | both | 6151.97(2460.93,10664.19) | 22012.33(13966.97,30722.68) | 2.58 | 58.02(23.01,101.21) | 154.77(97.84,213.66) | 3.21(2.53,3.89) |
| Vanuatu | both | 7.43(1.61,19.86) | 36.27(10.28,84.96) | 3.88 | 11.64(2.55,31.35) | 21.41(6.01,50.01) | 1.71(1.49,1.93) |
| Venezuela (Bolivarian Republic of) | both | 3096.65(1393.84,5122.63) | 7887.37(5060.60,11620.50) | 1.55 | 32.99(14.67,54.79) | 27.76(17.67,40.79) | -0.80(-0.96,-0.65) |
| Viet nam | both | 5418.17(2073.85,10894.45) | 25673.68(17381.15,34168.38) | 3.74 | 14.37(5.59,28.56) | 30.04(20.46,39.90) | 3.15(2.60,3.70) |
| Virginia | both | 1461.50(623.17,2582.34) | 475.94(172.09,823.54) | -0.67 | 20.93(8.90,36.83) | 3.36(1.21,5.79) | -6.66(-7.10,-6.23) |
| Yemen | both | 914.10(253.47,2306.67) | 7674.67(3743.27,12231.55) | 7.40 | 20.43(5.85,50.83) | 63.02(30.64,100.05) | 4.36(4.11,4.62) |
| Zambia | both | 202.09(75.44,433.63) | 1281.59(657.34,2106.16) | 5.34 | 7.92(3.06,16.74) | 21.31(10.93,34.69) | 3.66(3.46,3.87) |
| Zimbabwe | both | 355.74(163.44,623.99) | 944.01(443.80,1625.05) | 1.65 | 10.26(4.71,17.88) | 15.51(7.44,26.39) | 1.55(1.22,1.88) |
| Monaco | both | 9.00(0.89,19.58) | 7.35(4.24,10.87) | -0.18 | 11.62(1.16,25.17) | 6.89(3.93,10.16) | -1.44(-2.10,-0.78) |
| San Marino | both | 3.10(0.68,6.48) | 3.11(1.24,5.61) | 0.00 | 9.47(2.08,19.67) | 4.23(1.68,7.74) | -3.09(-3.52,-2.67) |
| Saint Kitts and Nevis | both | 9.24(3.13,18.51) | 6.85(3.09,11.26) | -0.26 | 25.21(8.53,50.29) | 11.63(5.20,19.18) | -3.13(-3.58,-2.69) |
| Cook Islands | both | 1.09(0.40,2.59) | 1.29(0.25,3.05) | 0.19 | 8.76(3.25,20.88) | 5.44(1.09,12.73) | -1.79(-2.13,-1.45) |
| Nauru | both | 0.82(0.23,2.37) | 0.82(0.22,1.90) | -0.01 | 19.46(5.32,56.37) | 17.16(4.62,39.20) | -1.08(-1.35,-0.80) |
| Niue | both | 0.29(0.11,0.64) | 0.26(0.05,0.59) | -0.13 | 13.33(5.19,28.92) | 12.01(2.47,27.63) | -0.92(-1.16,-0.68) |
| Palau | both | 1.37(0.01,4.26) | 2.48(0.01,6.37) | 0.81 | 13.78(0.13,42.39) | 11.82(0.05,29.90) | 0.33(-0.51,1.17) |
| Tokelau | both | 0.18(0.00,0.65) | 0.12(0.00,0.33) | -0.33 | 13.68(0.36,49.65) | 9.46(0.05,25.64) | -1.71(-2.02,-1.40) |
| Tuvalu | both | 0.59(0.15,1.70) | 1.35(0.50,2.92) | 1.28 | 8.81(2.19,25.42) | 13.58(5.12,29.50) | 1.13(0.88,1.38) |
| Afghanistan | female | 499.76(122.64,1331.49) | 2158.73(919.86,4265.93) | 3.32 | 15.79(4.09,41.72) | 35.38(15.50,69.08) | 3.10(2.49,3.71) |
| Albania | female | 201.52(91.77,345.33) | 537.40(374.26,729.50) | 1.67 | 19.84(9.01,34.03) | 23.00(16.00,31.19) | 1.19(0.74,1.64) |
| Algeria | female | 3434.04(2119.76,4921.91) | 7984.83(5513.01,10634.72) | 1.33 | 77.82(48.88,109.52) | 63.37(44.45,84.60) | -0.91(-1.09,-0.73) |
| American Samoa | female | 0.75(0.23,1.92) | 1.80(0.77,3.51) | 1.41 | 7.62(2.27,19.77) | 7.76(3.29,15.24) | -0.20(-0.47,0.08) |
| Andorra | female | 1.73(0.53,3.38) | 2.16(1.11,3.51) | 0.25 | 7.83(2.42,15.27) | 2.62(1.34,4.31) | -3.59(-3.77,-3.42) |
| Angola | female | 78.69(22.28,198.73) | 900.87(417.01,1580.32) | 10.45 | 4.81(1.36,11.81) | 18.08(8.35,31.54) | 4.59(4.36,4.83) |
| Antigua and Barbuda | female | 7.02(1.88,14.12) | 8.48(3.23,14.62) | 0.21 | 20.93(5.66,42.05) | 16.80(6.38,28.87) | -1.34(-1.65,-1.02) |
| Argentina | female | 3209.03(1226.54,5795.56) | 2689.10(1672.40,3803.95) | -0.16 | 18.31(7.00,33.05) | 8.27(5.15,11.65) | -2.85(-3.20,-2.51) |
| Armenia | female | 693.66(317.95,1153.81) | 1028.24(707.90,1375.34) | 0.48 | 51.56(23.59,85.92) | 42.43(29.31,56.77) | -0.95(-1.16,-0.73) |
| Australia | female | 735.55(96.20,1722.65) | 408.75(107.41,760.15) | -0.44 | 6.49(0.85,15.14) | 1.52(0.40,2.79) | -5.57(-5.89,-5.24) |
| Austria | female | 2093.55(1021.93,3307.73) | 700.41(503.45,923.99) | -0.67 | 24.96(12.36,39.30) | 5.35(3.89,6.99) | -5.73(-5.93,-5.52) |
| Azerbaijan | female | 1023.05(458.85,1833.50) | 2905.44(1733.82,4340.55) | 1.84 | 36.17(16.25,64.97) | 77.04(45.84,114.73) | 2.71(2.38,3.04) |
| Bahrain | female | 73.64(58.64,88.86) | 121.89(96.76,154.11) | 0.66 | 118.79(95.13,142.97) | 49.69(39.92,62.09) | -3.24(-3.54,-2.95) |
| Bangladesh | female | 1988.26(642.09,4379.71) | 16724.66(10239.03,24197.51) | 7.41 | 10.43(3.37,23.15) | 29.19(17.88,41.83) | 4.10(3.84,4.36) |
| Barbados | female | 50.21(14.49,94.62) | 46.60(20.30,77.33) | -0.07 | 26.90(7.88,50.53) | 16.76(7.31,27.78) | -1.76(-2.14,-1.38) |
| Belarus | female | 4687.00(2432.51,7227.26) | 3837.06(2701.70,5244.24) | -0.18 | 55.05(28.70,84.69) | 34.08(23.75,46.46) | -1.80(-2.30,-1.30) |
| Belgium | female | 2190.92(1033.09,3527.16) | 800.52(585.69,1031.21) | -0.63 | 21.80(10.38,34.88) | 4.93(3.66,6.27) | -5.23(-5.43,-5.03) |
| Belize | female | 6.03(1.32,14.89) | 17.44(6.36,30.87) | 1.89 | 13.05(2.85,32.16) | 13.62(4.86,24.27) | -0.59(-1.11,-0.07) |
| Benin | female | 59.88(20.17,139.39) | 283.20(131.83,513.74) | 3.73 | 6.45(2.18,14.97) | 12.47(5.89,22.61) | 2.30(1.98,2.62) |
| Bermuda | female | 4.33(1.07,10.89) | 1.74(0.35,3.38) | -0.60 | 12.49(3.10,31.35) | 2.07(0.43,4.01) | -7.14(-7.69,-6.59) |
| Bhutan | female | 5.87(1.61,14.53) | 46.79(27.21,72.12) | 6.98 | 5.20(1.45,12.55) | 18.48(10.74,28.20) | 5.33(5.01,5.64) |
| Bolivia (Plurinational State of) | female | 376.66(129.90,741.93) | 839.70(476.26,1300.43) | 1.23 | 23.61(8.27,46.38) | 19.80(11.31,30.44) | -0.89(-1.41,-0.38) |
| Bosnia and Herzegovina | female | 656.22(319.66,1066.28) | 1300.38(957.97,1653.25) | 0.98 | 34.10(16.62,55.06) | 38.47(28.33,48.96) | 0.49(0.08,0.90) |
| Botswana | female | 33.16(15.99,59.89) | 188.85(109.61,296.17) | 4.70 | 12.10(5.87,21.66) | 28.53(16.88,43.83) | 3.26(2.82,3.70) |
| Brazil | female | 7151.02(3171.25,13006.53) | 10173.26(7061.35,13589.98) | 0.42 | 16.18(7.13,29.56) | 7.85(5.45,10.49) | -2.62(-2.85,-2.38) |
| Brunei Darussalam | female | 5.72(2.27,11.19) | 7.16(2.09,12.60) | 0.25 | 13.22(5.24,26.36) | 5.86(1.66,10.37) | -2.77(-3.31,-2.23) |
| Bulgaria | female | 4286.36(2280.12,6419.55) | 3671.25(2827.53,4634.99) | -0.14 | 71.49(37.55,107.08) | 39.81(30.52,50.24) | -2.90(-3.30,-2.50) |
| Burkina Faso | female | 64.80(16.83,165.29) | 320.27(113.05,669.40) | 3.94 | 3.37(0.94,8.60) | 7.50(2.69,15.61) | 3.52(3.22,3.83) |
| Burundi | female | 66.42(17.25,173.73) | 112.57(37.06,260.34) | 0.69 | 5.68(1.51,14.79) | 6.14(2.01,14.06) | 0.00(-0.26,0.26) |
| Cambodia | female | 151.17(43.77,373.87) | 774.53(365.99,1328.42) | 4.12 | 6.67(1.98,16.38) | 12.68(6.07,21.83) | 2.46(2.25,2.68) |
| Cameroon | female | 261.32(113.26,493.69) | 1454.08(806.45,2176.88) | 4.56 | 13.51(6.07,25.23) | 27.23(15.48,40.49) | 2.59(2.41,2.77) |
| Canada | female | 1636.46(415.33,3328.62) | 810.72(382.34,1336.51) | -0.50 | 8.55(2.17,17.45) | 1.88(0.90,3.07) | -5.91(-6.23,-5.58) |
| Cabo Verde | female | 10.26(4.39,19.69) | 79.42(52.50,108.64) | 6.74 | 7.47(3.16,14.39) | 32.26(21.40,43.98) | 5.02(4.51,5.54) |
| Central African Republic | female | 37.62(8.82,95.98) | 107.39(32.93,263.06) | 1.85 | 7.08(1.70,18.42) | 10.90(3.41,26.84) | 1.58(1.36,1.81) |
| Chad | female | 59.91(14.93,163.78) | 204.33(73.04,448.65) | 2.41 | 4.58(1.16,12.36) | 9.15(3.40,20.09) | 2.80(2.59,3.02) |
| Chile | female | 1002.03(478.60,1679.86) | 1412.74(1112.18,1729.26) | 0.41 | 20.11(9.60,33.79) | 10.35(8.16,12.69) | -2.17(-2.33,-2.01) |
| China | female | 88261.21(42119.88,156341.88) | 360939.49(272076.37,457005.43) | 3.09 | 23.66(11.34,42.02) | 37.03(28.04,46.68) | 1.92(1.49,2.34) |
| Colombia | female | 1697.52(761.47,3044.56) | 3480.13(2347.84,4876.78) | 1.05 | 20.32(9.09,36.40) | 11.61(7.83,16.26) | -2.28(-2.43,-2.12) |
| Comoros | female | 3.85(1.29,9.01) | 19.69(9.24,35.68) | 4.12 | 3.67(1.25,8.46) | 7.96(3.74,14.44) | 2.54(2.41,2.67) |
| Congo | female | 82.51(29.17,179.34) | 445.56(238.30,731.85) | 4.40 | 15.90(5.68,34.84) | 38.37(20.35,62.46) | 3.12(2.89,3.35) |
| Costa Rica | female | 92.45(40.59,166.36) | 231.05(159.84,313.46) | 1.50 | 10.70(4.71,19.33) | 8.18(5.67,11.08) | -1.29(-1.59,-0.99) |
| Côte d'Ivoire | female | 135.13(46.81,302.10) | 719.54(337.11,1248.95) | 4.32 | 9.29(3.27,20.38) | 16.93(8.14,28.94) | 2.40(2.11,2.69) |
| Croatia | female | 1854.62(974.82,2857.46) | 1163.66(867.84,1497.58) | -0.37 | 50.44(26.37,77.49) | 19.17(14.18,24.57) | -3.44(-3.67,-3.21) |
| Cuba | female | 1465.08(550.74,2654.28) | 1627.33(807.01,2687.23) | 0.11 | 28.53(10.71,51.86) | 15.11(7.53,24.97) | -2.38(-2.62,-2.14) |
| Cyprus | female | 112.85(46.26,193.85) | 96.29(71.84,127.82) | -0.15 | 34.45(14.01,59.76) | 9.95(7.43,13.17) | -4.84(-5.03,-4.65) |
| Czechia | female | 5130.70(2686.55,7733.38) | 2088.99(1570.59,2677.79) | -0.59 | 59.49(31.34,89.92) | 15.01(11.36,19.25) | -4.99(-5.13,-4.84) |
| Democratic Republic of the Congo | female | 494.89(139.36,1222.89) | 1817.88(725.89,3566.34) | 2.67 | 7.33(2.17,17.80) | 10.83(4.33,21.13) | 0.96(0.34,1.58) |
| Denmark | female | 1168.94(483.10,1979.44) | 254.46(156.76,365.82) | -0.78 | 22.24(9.31,37.52) | 3.46(2.15,4.96) | -6.76(-7.04,-6.48) |
| Djibouti | female | 5.35(1.69,12.11) | 74.20(34.99,121.50) | 12.88 | 9.63(3.11,21.46) | 33.46(15.90,54.24) | 5.00(4.50,5.50) |
| Dominica | female | 7.68(2.62,15.62) | 8.51(3.57,14.91) | 0.11 | 16.88(5.78,34.13) | 17.09(7.18,29.81) | -0.09(-0.23,0.06) |
| Dominican Republic | female | 141.34(40.87,340.78) | 1104.64(493.42,2044.76) | 6.82 | 8.10(2.33,19.39) | 23.37(10.45,43.63) | 4.91(4.52,5.31) |
| Ecuador | female | 359.09(158.15,650.51) | 952.64(618.04,1348.95) | 1.65 | 14.25(6.25,25.55) | 12.85(8.20,18.08) | 0.10(-0.30,0.50) |
| Egypt | female | 14833.39(11569.53,17932.12) | 30610.41(21994.93,39722.87) | 1.06 | 116.11(90.43,140.58) | 135.18(98.74,171.96) | 0.74(0.57,0.91) |
| El Salvador | female | 146.52(56.49,288.49) | 495.55(298.04,763.90) | 2.38 | 9.34(3.58,18.43) | 13.69(8.31,21.07) | 1.48(1.20,1.76) |
| Equatorial Guinea | female | 5.86(1.34,16.47) | 88.23(47.59,139.11) | 14.06 | 6.07(1.39,17.28) | 38.40(21.14,59.67) | 7.71(7.22,8.21) |
| Eritrea | female | 28.75(7.33,76.61) | 209.71(87.50,406.97) | 6.30 | 5.86(1.45,15.72) | 16.54(7.06,32.01) | 3.49(3.24,3.74) |
| Estonia | female | 329.62(135.47,613.78) | 68.59(26.60,124.57) | -0.79 | 23.74(9.69,44.52) | 3.18(1.23,5.71) | -7.67(-8.34,-6.99) |
| Ethiopia | female | 255.62(68.86,683.08) | 1185.24(581.71,2126.58) | 3.64 | 3.03(0.84,7.95) | 6.88(3.44,12.39) | 2.82(2.52,3.12) |
| Micronesia (Federated States of) | female | 2.15(0.45,6.17) | 6.97(2.03,16.42) | 2.25 | 9.91(2.10,28.51) | 21.10(6.25,49.78) | 2.61(2.40,2.81) |
| Fiji | female | 15.11(3.46,43.33) | 62.19(19.26,139.35) | 3.12 | 9.07(2.13,25.96) | 17.52(5.37,39.40) | 2.10(1.51,2.70) |
| Finland | female | 499.09(61.15,1106.00) | 131.15(27.04,272.33) | -0.74 | 10.41(1.28,23.00) | 1.47(0.31,3.05) | -6.93(-7.44,-6.41) |
| France | female | 7097.42(3143.49,11879.67) | 3026.91(2119.57,4081.64) | -0.57 | 12.27(5.45,20.40) | 2.82(2.02,3.71) | -5.02(-5.22,-4.82) |
| Gabon | female | 44.95(18.77,85.80) | 179.42(106.39,266.21) | 2.99 | 15.74(6.53,29.91) | 36.17(21.51,53.20) | 2.79(2.24,3.35) |
| Georgia | female | 1709.82(822.97,3016.65) | 1158.07(788.81,1592.20) | -0.32 | 48.08(23.10,84.31) | 27.92(18.92,38.32) | -2.22(-2.47,-1.97) |
| Germany | female | 25098.35(11843.90,40404.65) | 7483.57(5398.57,9843.17) | -0.70 | 28.08(13.35,45.15) | 5.51(4.00,7.17) | -5.67(-5.91,-5.44) |
| Ghana | female | 548.16(243.19,996.16) | 3269.09(1889.25,4839.73) | 4.96 | 19.50(8.85,34.75) | 41.59(24.22,61.01) | 3.17(2.90,3.44) |
| Greece | female | 2540.43(1209.93,4149.00) | 1839.53(1388.06,2322.84) | -0.28 | 30.13(14.38,49.35) | 10.88(8.36,13.58) | -3.93(-4.37,-3.48) |
| Greenland | female | 1.70(0.41,4.49) | 1.02(0.11,2.90) | -0.40 | 11.43(2.77,30.63) | 3.53(0.40,10.18) | -5.34(-5.93,-4.76) |
| Grenada | female | 10.34(2.69,23.55) | 13.42(5.26,23.19) | 0.30 | 22.57(5.96,50.84) | 23.61(9.25,40.73) | -0.55(-1.00,-0.10) |
| Guam | female | 3.91(0.77,10.48) | 8.54(3.62,14.47) | 1.18 | 12.90(2.50,35.33) | 8.73(3.72,14.84) | -1.49(-2.36,-0.62) |
| Guatemala | female | 147.46(50.49,324.25) | 667.55(358.85,1021.43) | 3.53 | 9.62(3.32,21.06) | 11.81(6.41,17.94) | -0.01(-0.54,0.53) |
| Guinea | female | 90.99(27.25,229.46) | 288.48(120.01,605.83) | 2.17 | 6.14(1.88,15.44) | 11.52(4.88,23.83) | 2.67(2.49,2.86) |
| Guinea-Bissau | female | 14.17(4.07,36.78) | 55.15(23.41,110.09) | 2.89 | 7.69(2.22,19.80) | 15.84(6.95,30.86) | 2.80(2.70,2.89) |
| Guyana | female | 87.15(20.73,184.09) | 119.24(50.03,216.54) | 0.37 | 46.76(11.09,99.30) | 38.64(16.22,70.53) | -0.57(-0.75,-0.39) |
| Haiti | female | 130.22(33.76,336.33) | 478.63(169.14,1001.37) | 2.68 | 8.32(2.17,21.37) | 14.32(5.17,29.47) | 2.17(2.03,2.32) |
| Honduras | female | 60.69(20.54,133.85) | 461.28(237.81,764.07) | 6.60 | 5.90(1.97,12.98) | 15.90(8.38,26.38) | 3.69(3.09,4.30) |
| Hungary | female | 4022.17(2121.28,6164.51) | 2465.11(1860.14,3155.18) | -0.39 | 46.42(24.54,71.16) | 18.43(13.95,23.57) | -3.56(-3.72,-3.40) |
| Iceland | female | 8.90(0.84,22.22) | 3.05(0.63,6.45) | -0.66 | 5.18(0.49,12.95) | 0.81(0.17,1.69) | -6.71(-7.06,-6.35) |
| India | female | 30716.71(14577.92,53564.85) | 189415.21(135123.58,244026.59) | 5.17 | 16.68(7.99,29.41) | 34.42(24.82,44.39) | 2.67(2.44,2.90) |
| Indonesia | female | 8732.33(3824.02,16289.57) | 32489.55(22590.10,43293.08) | 2.72 | 18.54(8.07,34.96) | 32.60(22.79,43.12) | 1.79(1.52,2.06) |
| Iran (Islamic Republic of) | female | 6162.94(4900.83,7428.03) | 13227.93(11335.90,15169.73) | 1.15 | 62.33(49.11,75.54) | 41.77(35.54,48.13) | -1.91(-2.25,-1.57) |
| Iraq | female | 2910.38(1866.11,4001.67) | 8270.14(6304.57,10505.70) | 1.84 | 78.28(50.05,107.29) | 81.24(62.41,101.83) | 0.24(-0.05,0.53) |
| Ireland | female | 418.38(124.21,783.32) | 117.94(59.93,181.52) | -0.72 | 17.84(5.35,33.43) | 2.66(1.35,4.09) | -6.97(-7.22,-6.73) |
| Israel | female | 701.51(370.45,1066.53) | 475.16(372.42,582.31) | -0.32 | 27.20(14.62,41.30) | 6.51(5.14,7.93) | -5.60(-5.83,-5.37) |
| Italy | female | 12829.41(6910.63,19276.24) | 6861.70(5165.89,8684.71) | -0.47 | 23.19(12.51,34.85) | 6.07(4.69,7.56) | -4.84(-4.97,-4.71) |
| Jamaica | female | 103.09(44.19,199.66) | 237.36(147.01,347.89) | 1.30 | 10.33(4.44,19.91) | 14.18(8.82,20.75) | 0.86(0.53,1.19) |
| Japan | female | 9392.16(3088.23,17526.11) | 9062.50(5612.69,12985.23) | -0.04 | 9.96(3.26,18.67) | 3.26(2.19,4.52) | -4.11(-4.54,-3.68) |
| Jordan | female | 364.25(288.57,449.07) | 791.83(608.71,1026.84) | 1.17 | 68.74(54.46,84.75) | 33.56(25.99,43.39) | -3.14(-3.68,-2.60) |
| Kazakhstan | female | 2827.23(1335.57,5026.77) | 3676.36(2438.75,5151.01) | 0.30 | 37.99(17.96,67.56) | 40.59(26.83,56.84) | -0.45(-0.91,0.01) |
| Kenya | female | 145.44(64.16,280.98) | 911.77(468.95,1516.08) | 5.27 | 4.05(1.80,7.74) | 9.15(4.73,15.49) | 3.62(3.34,3.90) |
| Kiribati | female | 1.36(0.34,3.69) | 3.58(1.06,9.06) | 1.63 | 6.80(1.71,18.14) | 9.76(2.80,24.31) | 0.41(0.11,0.71) |
| Kuwait | female | 123.87(107.49,141.69) | 210.82(166.72,262.00) | 0.70 | 60.21(51.41,69.56) | 24.08(18.91,30.04) | -2.52(-3.02,-2.01) |
| Kyrgyzstan | female | 471.41(185.63,886.68) | 890.02(517.17,1336.65) | 0.89 | 25.49(9.98,47.67) | 38.98(22.70,58.76) | 1.26(0.80,1.73) |
| Lao People's Democratic Republic | female | 68.99(19.79,169.80) | 285.50(145.49,477.52) | 3.14 | 7.11(2.12,17.12) | 14.32(7.32,23.84) | 2.31(2.05,2.57) |
| Latvia | female | 1246.31(627.45,1964.51) | 540.58(352.06,765.96) | -0.57 | 51.10(25.59,80.37) | 16.98(11.14,24.04) | -4.01(-4.47,-3.55) |
| Lebanon | female | 444.94(300.38,591.00) | 997.52(626.64,1292.72) | 1.24 | 43.98(29.77,58.49) | 34.99(22.08,45.65) | -0.90(-1.06,-0.73) |
| Lesotho | female | 39.01(17.60,76.05) | 146.26(71.59,247.64) | 2.75 | 7.56(3.43,14.62) | 22.40(11.29,37.38) | 4.99(4.52,5.45) |
| Liberia | female | 35.03(12.69,79.96) | 111.68(51.92,210.47) | 2.19 | 7.73(2.85,17.53) | 12.85(6.05,23.91) | 2.95(2.42,3.47) |
| Libya | female | 332.96(203.52,484.67) | 1175.90(832.22,1600.62) | 2.53 | 40.11(24.46,58.64) | 49.62(35.36,67.51) | 0.55(0.29,0.81) |
| Lithuania | female | 1063.57(497.52,1769.66) | 592.68(389.80,850.97) | -0.44 | 36.73(17.09,61.27) | 12.87(8.45,18.41) | -3.53(-4.02,-3.04) |
| Luxembourg | female | 72.37(28.44,126.81) | 22.11(13.98,30.87) | -0.69 | 20.69(8.19,36.17) | 3.26(2.10,4.58) | -6.37(-6.67,-6.08) |
| North Macedonia | female | 680.21(390.86,971.33) | 1043.74(799.56,1319.05) | 0.53 | 80.11(45.74,114.09) | 70.14(54.55,87.76) | -0.55(-0.90,-0.21) |
| Madagascar | female | 83.10(30.62,186.87) | 405.62(170.45,819.21) | 3.88 | 3.59(1.32,7.99) | 8.26(3.57,16.36) | 3.10(2.93,3.26) |
| Malawi | female | 52.25(16.03,138.52) | 166.62(70.44,332.43) | 2.19 | 3.00(0.94,7.83) | 4.69(2.00,9.41) | 2.09(1.83,2.35) |
| Malaysia | female | 2041.43(939.28,3156.97) | 2616.76(1774.50,3636.56) | 0.28 | 47.58(21.71,73.75) | 21.95(14.82,30.06) | -2.69(-2.85,-2.52) |
| Maldives | female | 3.55(1.03,8.26) | 9.11(5.54,13.49) | 1.57 | 11.39(3.27,26.52) | 7.70(4.67,11.47) | -2.34(-2.72,-1.96) |
| Mali | female | 114.08(29.52,308.16) | 369.84(133.10,799.47) | 2.24 | 6.38(1.65,17.06) | 10.31(3.84,21.77) | 1.95(1.81,2.08) |
| Malta | female | 58.30(24.45,99.11) | 41.84(29.45,54.69) | -0.28 | 24.37(10.20,41.55) | 7.09(5.09,9.19) | -4.36(-4.63,-4.10) |
| Marshall Islands | female | 0.55(0.12,1.46) | 2.67(0.85,5.80) | 3.85 | 7.11(1.61,19.08) | 17.57(5.58,37.74) | 2.83(2.35,3.31) |
| Mauritania | female | 90.31(38.65,180.98) | 289.79(161.71,430.91) | 2.21 | 18.59(7.98,36.81) | 31.50(17.76,46.59) | 1.72(1.53,1.90) |
| Mauritius | female | 100.82(51.53,162.28) | 115.92(56.66,182.16) | 0.15 | 26.77(13.57,43.00) | 12.60(6.17,19.82) | -3.92(-4.59,-3.26) |
| Mexico | female | 3652.97(1869.14,5788.39) | 7314.62(5365.18,9635.17) | 1.00 | 18.92(9.68,30.18) | 12.39(9.06,16.29) | -1.92(-2.23,-1.61) |
| Republic of Moldova | female | 1078.61(485.76,1842.59) | 900.10(516.87,1327.16) | -0.17 | 45.93(20.78,78.34) | 24.66(14.10,36.17) | -2.07(-2.66,-1.47) |
| Mongolia | female | 179.07(76.97,338.90) | 721.71(460.06,1005.79) | 3.03 | 33.06(13.96,62.47) | 65.61(42.97,90.46) | 2.10(1.72,2.49) |
| Montenegro | female | 156.67(84.55,235.14) | 214.55(162.69,267.11) | 0.37 | 46.57(25.21,69.79) | 38.99(29.35,48.45) | -0.38(-0.70,-0.06) |
| Morocco | female | 1909.14(974.61,3137.26) | 9720.71(7188.22,12585.59) | 4.09 | 30.51(15.76,49.91) | 69.02(51.76,88.37) | 2.94(2.62,3.26) |
| Mozambique | female | 50.65(13.05,142.71) | 237.82(95.14,504.79) | 3.69 | 1.91(0.51,5.42) | 4.53(1.88,9.49) | 3.94(3.46,4.42) |
| Myanmar | female | 1591.06(544.46,3526.78) | 5825.94(3493.27,8634.31) | 2.66 | 14.01(4.86,30.56) | 24.73(14.85,36.67) | 2.22(1.95,2.49) |
| Namibia | female | 46.27(22.16,83.69) | 158.24(87.39,246.32) | 2.42 | 13.10(6.28,23.77) | 21.54(12.03,33.26) | 1.61(1.29,1.94) |
| Nepal | female | 317.47(88.87,768.12) | 2107.70(1220.63,3205.11) | 5.64 | 7.52(2.17,18.18) | 19.94(11.74,30.29) | 3.87(3.55,4.19) |
| Netherlands | female | 2401.08(1136.09,3933.45) | 941.76(687.45,1234.35) | -0.61 | 18.89(9.00,30.68) | 4.38(3.21,5.68) | -5.57(-5.83,-5.30) |
| New Zealand | female | 137.10(10.72,355.95) | 75.82(14.18,154.11) | -0.45 | 6.02(0.48,15.45) | 1.58(0.29,3.19) | -5.19(-5.44,-4.95) |
| Nicaragua | female | 27.27(9.67,59.41) | 234.95(125.77,376.72) | 7.62 | 3.45(1.22,7.55) | 11.15(5.98,17.98) | 3.83(3.07,4.60) |
| Niger | female | 62.96(12.97,198.19) | 274.55(74.62,732.99) | 3.36 | 5.77(1.26,17.77) | 8.49(2.46,21.94) | 1.30(1.14,1.46) |
| Nigeria | female | 2312.48(966.44,4642.93) | 9595.37(5512.63,14416.96) | 3.15 | 12.15(5.10,24.43) | 26.32(15.51,39.04) | 2.76(2.44,3.08) |
| Democratic People's Republic of Korea | female | 1436.97(574.91,2796.78) | 5927.33(3574.89,8805.05) | 3.12 | 16.68(6.62,32.32) | 31.14(18.74,46.29) | 2.42(2.27,2.56) |
| Northern Mariana Islands | female | 1.15(0.38,2.82) | 2.06(1.15,3.33) | 0.80 | 17.16(5.69,41.75) | 9.49(5.23,15.47) | -2.10(-2.72,-1.49) |
| Norway | female | 538.88(146.12,1050.68) | 91.76(34.01,162.10) | -0.83 | 11.91(3.23,23.22) | 1.41(0.52,2.50) | -7.69(-8.01,-7.38) |
| Oman | female | 168.35(88.43,273.01) | 462.42(356.84,573.50) | 1.75 | 65.64(34.57,105.86) | 86.75(66.10,107.58) | 0.56(0.02,1.11) |
| Pakistan | female | 2983.07(957.07,6340.27) | 20229.04(12699.00,29211.93) | 5.78 | 12.38(4.04,26.58) | 40.88(26.03,58.45) | 4.49(4.27,4.70) |
| Palestine | female | 150.37(68.40,249.75) | 542.31(403.50,673.99) | 2.61 | 35.25(16.23,58.20) | 53.26(39.66,66.25) | 0.79(0.35,1.24) |
| Panama | female | 79.27(34.14,148.71) | 140.63(82.14,209.26) | 0.77 | 11.06(4.77,20.72) | 6.26(3.67,9.29) | -1.52(-1.77,-1.28) |
| Papua New Guinea | female | 26.78(4.74,87.40) | 127.90(28.90,332.40) | 3.78 | 3.42(0.62,10.90) | 6.41(1.44,16.74) | 2.27(2.10,2.44) |
| Paraguay | female | 90.39(36.64,175.09) | 235.22(139.40,359.21) | 1.60 | 7.98(3.23,15.46) | 8.18(4.84,12.48) | -0.07(-0.30,0.17) |
| Peru | female | 830.83(380.27,1471.53) | 1662.51(1075.52,2400.99) | 1.00 | 14.20(6.50,25.15) | 9.69(6.24,13.97) | -1.68(-2.20,-1.16) |
| Philippines | female | 1267.37(604.71,2166.41) | 7255.67(4694.32,10096.44) | 4.72 | 11.80(5.54,20.22) | 19.06(12.52,26.45) | 1.93(1.13,2.74) |
| Poland | female | 13380.67(7558.55,19664.05) | 9069.94(6970.43,11601.50) | -0.32 | 51.36(29.11,75.88) | 18.81(14.36,24.12) | -4.02(-4.21,-3.83) |
| Portugal | female | 1446.36(480.58,2757.56) | 541.34(317.36,791.80) | -0.63 | 17.97(5.96,34.26) | 3.08(1.82,4.46) | -6.66(-7.06,-6.25) |
| Puerto Rico | female | 113.21(0.99,370.02) | 80.98(16.87,151.25) | -0.28 | 5.88(0.05,19.15) | 1.81(0.39,3.38) | -4.95(-5.57,-4.34) |
| Qatar | female | 35.42(27.68,43.76) | 90.28(69.84,115.42) | 1.55 | 120.77(91.20,147.56) | 102.44(84.25,124.43) | -0.43(-0.80,-0.07) |
| Romania | female | 6537.13(3357.33,10379.00) | 5873.66(4477.02,7456.97) | -0.10 | 46.18(23.62,73.55) | 23.87(18.18,30.24) | -2.89(-3.25,-2.53) |
| Russian Federation | female | 56882.94(24343.45,95283.92) | 32937.80(19001.01,49021.51) | -0.42 | 48.53(20.90,81.78) | 20.57(11.78,30.59) | -3.25(-3.85,-2.66) |
| Rwanda | female | 113.33(33.79,281.26) | 303.92(127.29,593.00) | 1.68 | 7.95(2.45,19.31) | 10.54(4.36,20.35) | 0.69(0.31,1.07) |
| Saint Lucia | female | 10.71(3.46,22.81) | 18.77(7.83,32.02) | 0.75 | 22.63(7.29,48.24) | 16.32(6.83,27.85) | -2.06(-2.53,-1.59) |
| Saint Vincent and the Grenadines | female | 9.16(2.48,20.69) | 16.71(6.50,29.15) | 0.82 | 22.98(6.22,51.57) | 26.19(10.16,45.86) | 0.13(-0.19,0.47) |
| Samoa | female | 4.60(1.08,12.19) | 10.45(2.77,24.30) | 1.27 | 10.91(2.55,28.99) | 14.70(3.91,34.18) | 0.74(0.64,0.84) |
| Sao Tome and Principe | female | 2.28(0.90,4.68) | 11.60(5.58,19.67) | 4.09 | 7.40(2.98,15.17) | 24.06(11.33,40.76) | 4.50(4.26,4.75) |
| Saudi Arabia | female | 1065.20(562.00,1681.49) | 4906.08(3760.16,6293.69) | 3.61 | 47.96(25.92,75.13) | 76.72(60.28,96.56) | 0.94(0.22,1.67) |
| Senegal | female | 131.43(46.66,296.12) | 565.76(267.44,1030.44) | 3.30 | 9.50(3.43,21.22) | 16.54(7.87,29.95) | 1.77(1.60,1.95) |
| Serbia | female | 3496.78(1842.42,5222.75) | 4118.44(3191.00,5153.99) | 0.18 | 65.58(34.62,99.30) | 47.22(36.63,58.41) | -1.44(-1.85,-1.03) |
| Seychelles | female | 5.45(2.88,8.95) | 7.70(3.93,11.95) | 0.41 | 16.38(8.76,26.68) | 13.94(7.10,21.57) | -0.73(-0.87,-0.58) |
| Sierra Leone | female | 58.13(18.83,146.27) | 213.60(90.70,424.33) | 2.67 | 6.94(2.24,17.52) | 13.20(5.71,25.96) | 2.85(2.57,3.14) |
| Singapore | female | 299.65(104.38,529.02) | 261.98(163.91,363.16) | -0.13 | 27.56(9.57,48.87) | 6.48(4.05,8.96) | -5.38(-5.75,-5.01) |
| Slovakia | female | 2114.14(1139.85,3134.89) | 1356.97(1005.74,1750.01) | -0.36 | 60.49(32.58,90.09) | 23.41(17.35,30.21) | -3.48(-3.69,-3.28) |
| Slovenia | female | 468.42(246.78,772.81) | 229.12(159.87,323.11) | -0.51 | 30.85(16.36,51.01) | 7.06(4.98,9.78) | -5.71(-5.94,-5.49) |
| Solomon Islands | female | 3.39(0.60,10.40) | 21.56(5.83,58.30) | 5.37 | 5.81(1.05,17.34) | 15.03(4.07,40.65) | 3.12(2.94,3.29) |
| Somalia | female | 21.20(4.11,65.70) | 77.16(16.95,225.69) | 2.64 | 1.86(0.37,5.71) | 2.50(0.58,7.06) | 1.61(1.30,1.93) |
| South Africa | female | 2043.35(1476.61,2668.77) | 5168.37(4039.55,6393.54) | 1.53 | 17.59(12.64,23.01) | 21.55(16.65,26.68) | 0.94(0.46,1.42) |
| Republic of Korea | female | 6944.68(3817.13,10466.42) | 5127.48(3995.64,6470.39) | -0.26 | 47.45(25.31,72.10) | 9.89(7.72,12.48) | -6.00(-6.38,-5.62) |
| South Sudan | female | 51.51(17.00,118.26) | 118.66(46.15,228.72) | 1.30 | 5.34(1.78,12.26) | 7.95(3.15,15.15) | 1.77(1.59,1.95) |
| Spain | female | 4247.65(1643.43,7587.87) | 2020.32(1339.73,2812.50) | -0.52 | 12.84(4.98,22.94) | 2.75(1.84,3.80) | -5.47(-5.75,-5.19) |
| Sri Lanka | female | 570.77(236.53,1078.72) | 1812.78(1095.69,2746.35) | 2.18 | 13.01(5.45,24.05) | 13.99(8.40,21.14) | 0.57(0.13,1.02) |
| Sudan | female | 673.02(234.46,1513.97) | 4505.04(2531.58,6995.31) | 5.69 | 16.58(5.83,36.89) | 58.37(33.03,87.34) | 4.70(4.52,4.89) |
| Suriname | female | 37.78(11.89,74.36) | 73.03(34.82,122.33) | 0.93 | 28.54(8.96,56.24) | 22.99(11.00,38.49) | -0.92(-1.28,-0.56) |
| Eswatini | female | 14.33(6.59,25.34) | 60.66(31.11,99.52) | 3.23 | 9.87(4.57,17.40) | 20.69(10.92,33.69) | 2.86(2.45,3.28) |
| Sweden | female | 926.92(165.53,1931.28) | 179.41(48.54,363.87) | -0.81 | 9.17(1.64,19.21) | 1.24(0.33,2.50) | -6.87(-7.30,-6.44) |
| Switzerland | female | 1115.45(470.74,1866.45) | 371.49(240.24,527.36) | -0.67 | 15.46(6.67,25.67) | 2.84(1.85,3.99) | -5.98(-6.17,-5.79) |
| Syrian Arab Republic | female | 1889.15(1309.85,2530.93) | 3679.29(2694.78,4923.44) | 0.95 | 83.24(58.32,110.58) | 81.76(61.47,107.58) | -0.52(-0.80,-0.24) |
| Taiwan (Province of China) | female | 1539.37(795.87,2448.47) | 1749.11(1329.92,2261.10) | 0.14 | 23.64(12.20,38.18) | 8.05(6.13,10.43) | -3.97(-4.20,-3.73) |
| Tajikistan | female | 342.91(118.35,737.40) | 1438.83(720.58,2309.66) | 3.20 | 22.17(7.64,47.82) | 75.66(37.73,120.17) | 4.28(4.00,4.57) |
| United Republic of Tanzania | female | 140.68(50.69,317.64) | 835.81(420.69,1465.15) | 4.94 | 2.89(1.06,6.32) | 7.37(3.70,12.77) | 4.02(3.73,4.32) |
| Thailand | female | 2902.15(1361.76,4890.49) | 6983.82(4814.89,9506.47) | 1.41 | 17.30(7.99,28.98) | 12.62(8.70,17.17) | -1.58(-2.08,-1.07) |
| Bahamas | female | 16.88(4.45,33.13) | 26.25(7.67,50.15) | 0.56 | 20.11(5.25,39.62) | 12.98(3.76,24.90) | -1.73(-1.98,-1.49) |
| Gambia | female | 9.61(3.13,22.63) | 83.37(41.39,144.78) | 7.68 | 6.78(2.24,15.50) | 18.48(9.23,32.02) | 3.69(3.49,3.89) |
| Timor-Leste | female | 4.01(0.97,10.64) | 45.58(18.97,84.76) | 10.36 | 3.57(0.90,9.41) | 12.67(5.28,23.70) | 5.65(5.05,6.26) |
| Togo | female | 50.98(19.28,102.17) | 269.97(128.75,463.17) | 4.30 | 9.11(3.54,17.79) | 15.24(7.41,25.97) | 1.82(1.49,2.15) |
| Tonga | female | 1.37(0.35,3.66) | 3.65(1.10,8.10) | 1.66 | 5.26(1.33,14.13) | 8.41(2.55,18.70) | 1.45(1.27,1.63) |
| Trinidad and Tobago | female | 177.22(35.90,358.89) | 211.85(70.97,387.79) | 0.20 | 41.84(8.37,84.96) | 21.91(7.38,40.15) | -3.05(-3.47,-2.64) |
| Tunisia | female | 856.04(542.94,1185.19) | 2422.39(1637.29,3331.50) | 1.83 | 41.34(26.19,57.11) | 40.08(27.12,55.08) | -0.26(-0.64,0.11) |
| Turkey | female | 5471.80(3929.36,7001.86) | 11096.62(8385.60,14197.49) | 1.03 | 31.74(22.73,40.87) | 24.50(18.56,31.35) | -0.76(-1.12,-0.41) |
| Turkmenistan | female | 701.65(291.17,1191.31) | 1321.69(785.26,1971.60) | 0.88 | 68.74(28.35,117.02) | 67.45(39.89,100.59) | -0.81(-1.17,-0.44) |
| Uganda | female | 81.70(23.34,207.91) | 558.78(263.84,1004.02) | 5.84 | 2.94(0.89,7.38) | 8.35(3.93,14.78) | 4.22(3.96,4.48) |
| Ukraine | female | 26303.77(12299.74,41891.92) | 19525.27(11911.54,29289.24) | -0.26 | 56.21(26.20,89.65) | 37.29(22.73,55.79) | -1.84(-2.27,-1.42) |
| United Arab Emirates | female | 108.19(81.22,141.59) | 366.56(259.86,499.48) | 2.39 | 105.97(81.39,139.58) | 52.60(38.56,70.65) | -2.10(-3.19,-0.99) |
| United Kingdom | female | 13094.04(5389.07,22097.35) | 3093.72(2041.07,4296.93) | -0.76 | 22.31(9.20,37.47) | 3.88(2.58,5.34) | -6.61(-6.84,-6.38) |
| United States of America | female | 28856.50(10949.25,52419.14) | 11294.54(5990.39,17332.25) | -0.61 | 14.22(5.46,25.65) | 3.38(1.80,5.15) | -5.44(-5.79,-5.10) |
| Uruguay | female | 267.09(88.94,562.50) | 188.92(92.91,301.18) | -0.29 | 11.78(3.94,24.69) | 5.14(2.58,8.16) | -3.13(-3.33,-2.92) |
| Uzbekistan | female | 2790.90(1073.04,5020.65) | 9471.04(6006.31,13217.38) | 2.39 | 44.49(17.08,80.15) | 131.70(83.25,182.55) | 3.57(2.88,4.26) |
| Vanuatu | female | 1.80(0.39,5.08) | 10.49(2.87,24.82) | 4.83 | 7.00(1.52,19.64) | 13.98(3.89,33.08) | 1.95(1.69,2.21) |
| Venezuela (Bolivarian Republic of) | female | 1323.53(566.78,2205.14) | 3116.98(2001.44,4520.93) | 1.36 | 27.02(11.50,45.33) | 20.41(13.08,29.54) | -1.21(-1.46,-0.96) |
| Viet nam | female | 2005.35(742.62,4126.74) | 8848.05(5626.71,12336.77) | 3.41 | 9.04(3.34,18.52) | 18.09(11.57,25.09) | 3.06(2.51,3.60) |
| Virginia | female | 665.51(281.74,1175.94) | 208.51(76.15,373.08) | -0.69 | 15.46(6.58,27.23) | 2.53(0.93,4.52) | -6.59(-7.08,-6.10) |
| Yemen | female | 340.25(94.58,891.09) | 3071.66(1468.76,5080.95) | 8.03 | 14.99(4.21,38.73) | 51.33(24.66,84.10) | 4.79(4.54,5.04) |
| Zambia | female | 68.42(26.03,145.98) | 445.47(225.73,756.83) | 5.51 | 5.79(2.25,12.07) | 14.82(7.52,25.02) | 3.55(3.18,3.91) |
| Zimbabwe | female | 143.59(66.72,255.86) | 438.83(201.51,778.60) | 2.06 | 8.10(3.77,14.42) | 12.81(5.87,22.69) | 2.50(2.08,2.92) |
| Monaco | female | 4.42(0.44,9.72) | 3.57(2.02,5.35) | -0.19 | 8.64(0.88,18.78) | 5.39(3.01,8.01) | -1.21(-1.88,-0.53) |
| San Marino | female | 1.44(0.31,3.04) | 1.48(0.57,2.70) | 0.03 | 7.46(1.63,15.67) | 3.30(1.27,6.12) | -2.95(-3.32,-2.58) |
| Saint Kitts and Nevis | female | 4.76(1.69,9.64) | 2.83(1.30,4.66) | -0.41 | 22.61(8.00,45.50) | 9.43(4.32,15.46) | -3.04(-3.46,-2.62) |
| Cook Islands | female | 0.34(0.13,0.82) | 0.46(0.11,1.08) | 0.35 | 5.99(2.29,14.47) | 3.74(0.85,8.70) | -1.77(-2.14,-1.39) |
| Nauru | female | 0.22(0.06,0.65) | 0.30(0.08,0.71) | 0.35 | 12.81(3.35,37.58) | 13.86(4.00,32.25) | -0.32(-0.54,-0.09) |
| Niue | female | 0.12(0.04,0.27) | 0.11(0.03,0.25) | -0.12 | 8.77(3.18,19.75) | 8.88(2.14,20.72) | -0.49(-0.69,-0.29) |
| Palau | female | 0.42(0.00,1.31) | 0.73(0.00,1.87) | 0.73 | 8.94(0.10,28.02) | 7.67(0.04,20.06) | 0.38(-0.46,1.22) |
| Tokelau | female | 0.10(0.00,0.36) | 0.06(0.00,0.18) | -0.36 | 13.90(0.38,50.69) | 10.07(0.06,27.54) | -1.59(-1.91,-1.27) |
| Tuvalu | female | 0.21(0.05,0.60) | 0.56(0.22,1.23) | 1.67 | 5.78(1.43,16.68) | 11.07(4.24,24.18) | 1.87(1.56,2.19) |
| Afghanistan | male | 791.46(190.88,2040.77) | 2595.80(1130.08,4799.46) | 2.28 | 22.82(5.69,58.05) | 43.72(19.69,79.11) | 2.55(2.03,3.07) |
| Albania | male | 338.88(161.84,546.81) | 708.04(493.52,979.75) | 1.09 | 43.12(20.55,69.64) | 37.43(26.49,51.22) | -0.06(-0.32,0.20) |
| Algeria | male | 4538.58(2906.39,6312.85) | 8878.85(5978.22,12211.70) | 0.96 | 89.86(58.03,123.45) | 58.78(39.95,79.86) | -1.91(-2.14,-1.68) |
| American Samoa | male | 1.84(0.59,4.73) | 2.61(0.91,5.22) | 0.41 | 15.51(4.98,39.39) | 11.40(3.97,22.57) | -1.33(-1.57,-1.09) |
| Andorra | male | 3.12(0.99,5.90) | 2.74(1.46,4.23) | -0.12 | 12.65(3.87,23.75) | 3.97(2.13,6.16) | -3.84(-4.04,-3.64) |
| Angola | male | 170.62(50.10,419.03) | 1371.61(697.77,2287.79) | 7.04 | 9.48(2.87,23.35) | 29.90(14.72,49.23) | 4.10(3.82,4.38) |
| Antigua and Barbuda | male | 8.31(2.22,16.18) | 8.59(3.33,14.99) | 0.03 | 37.17(9.91,72.31) | 19.64(7.56,34.20) | -2.36(-2.75,-1.97) |
| Argentina | male | 4690.92(1856.00,8253.09) | 3476.98(2224.27,4971.56) | -0.26 | 34.25(13.51,60.56) | 15.10(9.59,21.50) | -2.97(-3.28,-2.67) |
| Armenia | male | 801.41(401.37,1246.92) | 1181.80(811.61,1555.25) | 0.47 | 80.10(40.15,126.02) | 70.49(48.42,92.86) | -0.78(-1.02,-0.54) |
| Australia | male | 932.75(96.73,2207.40) | 519.68(131.42,948.73) | -0.44 | 11.34(1.18,26.77) | 2.69(0.68,4.91) | -5.51(-5.84,-5.18) |
| Austria | male | 1930.69(960.69,2996.52) | 769.09(569.34,982.47) | -0.60 | 44.59(22.19,68.88) | 9.68(7.16,12.32) | -5.62(-5.80,-5.44) |
| Azerbaijan | male | 1297.22(612.47,2229.66) | 3508.80(2061.03,5198.37) | 1.70 | 69.02(32.49,120.30) | 104.36(60.50,152.92) | 1.36(1.08,1.64) |
| Bahrain | male | 131.57(107.18,157.24) | 232.16(177.89,300.34) | 0.76 | 149.39(121.58,178.06) | 51.72(40.59,66.71) | -3.95(-4.27,-3.63) |
| Bangladesh | male | 4040.89(1232.83,8977.20) | 29481.98(18182.43,42849.30) | 6.30 | 16.60(5.20,36.36) | 45.59(28.03,66.15) | 3.89(3.59,4.19) |
| Barbados | male | 45.01(13.16,83.24) | 44.40(19.54,73.48) | -0.01 | 37.31(10.87,68.97) | 20.52(9.08,34.01) | -2.88(-3.31,-2.45) |
| Belarus | male | 3877.06(2086.46,5848.23) | 3744.97(2548.86,5189.63) | -0.03 | 88.46(46.53,134.49) | 66.67(45.89,91.65) | -0.95(-1.59,-0.30) |
| Belgium | male | 2244.20(1072.69,3542.52) | 874.98(656.68,1102.87) | -0.61 | 37.23(17.81,58.81) | 8.49(6.42,10.71) | -5.20(-5.40,-5.00) |
| Belize | male | 8.13(1.91,17.70) | 29.40(11.47,51.92) | 2.62 | 18.13(4.28,39.61) | 21.74(8.43,38.31) | -0.53(-1.24,0.19) |
| Benin | male | 98.74(31.94,226.47) | 409.39(180.20,751.77) | 3.15 | 10.99(3.61,25.08) | 20.23(9.18,36.59) | 2.07(1.79,2.35) |
| Bermuda | male | 6.76(1.42,17.50) | 2.99(0.50,5.98) | -0.56 | 26.42(5.55,68.27) | 5.33(0.89,10.65) | -5.79(-6.28,-5.30) |
| Bhutan | male | 8.52(2.15,21.10) | 81.92(46.06,123.42) | 8.62 | 8.11(2.19,20.19) | 30.26(17.27,45.14) | 5.69(5.30,6.09) |
| Bolivia (Plurinational State of) | male | 472.44(176.14,874.50) | 977.41(544.94,1465.11) | 1.07 | 34.81(13.06,64.33) | 25.44(14.53,37.62) | -1.20(-1.62,-0.77) |
| Bosnia and Herzegovina | male | 815.85(422.26,1273.54) | 1273.93(961.24,1646.75) | 0.56 | 53.04(27.55,83.02) | 52.29(39.69,66.83) | -0.06(-0.41,0.29) |
| Botswana | male | 53.89(24.79,95.56) | 240.20(150.40,352.87) | 3.46 | 23.15(10.67,40.30) | 44.67(28.52,64.17) | 1.77(1.00,2.56) |
| Brazil | male | 11034.37(5143.98,19094.71) | 13692.83(9631.03,18093.71) | 0.24 | 27.03(12.55,47.17) | 13.06(9.12,17.30) | -2.53(-2.77,-2.29) |
| Brunei Darussalam | male | 9.54(3.37,19.10) | 12.34(3.58,22.16) | 0.29 | 22.92(8.08,46.71) | 10.89(3.16,19.47) | -1.92(-2.93,-0.90) |
| Bulgaria | male | 5657.28(3175.26,8440.44) | 3887.73(2982.58,4926.67) | -0.31 | 112.64(62.34,167.69) | 66.00(50.85,83.12) | -2.58(-2.98,-2.18) |
| Burkina Faso | male | 124.71(31.29,322.35) | 556.14(191.29,1174.32) | 3.46 | 6.90(1.77,17.76) | 15.02(5.17,31.44) | 3.32(3.09,3.55) |
| Burundi | male | 121.35(32.74,319.13) | 228.94(73.91,525.13) | 0.89 | 11.89(3.31,31.08) | 10.73(3.58,24.65) | -0.55(-0.88,-0.22) |
| Cambodia | male | 198.85(54.53,501.51) | 1024.47(481.44,1758.90) | 4.15 | 11.24(3.19,27.93) | 22.97(10.86,38.97) | 2.88(2.63,3.12) |
| Cameroon | male | 395.62(169.05,710.21) | 2250.84(1285.92,3304.09) | 4.69 | 20.93(9.36,37.21) | 43.60(25.31,63.46) | 2.60(2.39,2.81) |
| Canada | male | 2169.04(546.30,4333.15) | 1062.48(499.51,1706.27) | -0.51 | 15.95(4.02,31.91) | 3.42(1.61,5.48) | -5.95(-6.26,-5.63) |
| Cabo Verde | male | 16.17(6.77,30.34) | 103.91(71.16,134.88) | 5.43 | 16.60(6.88,31.14) | 60.53(41.98,78.54) | 3.35(2.69,4.02) |
| Central African Republic | male | 76.06(19.19,188.23) | 188.64(57.95,430.86) | 1.48 | 14.65(3.79,36.43) | 19.86(6.37,45.05) | 1.05(0.82,1.28) |
| Chad | male | 91.09(22.55,253.07) | 376.60(129.60,825.49) | 3.13 | 7.34(1.89,20.54) | 13.90(5.03,30.06) | 2.43(2.28,2.59) |
| Chile | male | 1287.81(632.48,2071.34) | 1836.61(1468.18,2202.79) | 0.43 | 30.82(15.00,49.92) | 17.41(13.89,20.92) | -1.84(-1.97,-1.72) |
| China | male | 134953.39(61939.49,227442.90) | 554728.88(423802.09,687505.38) | 3.11 | 41.36(19.26,69.11) | 68.90(53.92,84.39) | 2.28(1.90,2.66) |
| Colombia | male | 2335.64(1107.44,3903.65) | 4046.21(2734.43,5693.09) | 0.73 | 29.24(14.00,49.63) | 16.85(11.40,23.74) | -2.14(-2.34,-1.95) |
| Comoros | male | 6.11(1.96,14.06) | 23.13(10.63,41.01) | 2.79 | 6.13(2.01,13.86) | 11.23(5.24,19.89) | 1.88(1.70,2.07) |
| Congo | male | 124.30(43.14,250.40) | 487.39(270.30,766.61) | 2.92 | 27.68(9.61,55.62) | 43.15(24.19,67.80) | 1.26(0.92,1.59) |
| Costa Rica | male | 142.91(66.78,250.02) | 347.58(236.37,481.91) | 1.43 | 17.52(8.20,30.57) | 14.87(10.09,20.71) | -0.81(-1.03,-0.59) |
| Côte d'Ivoire | male | 337.16(112.17,754.82) | 1411.55(660.55,2380.07) | 3.19 | 18.18(6.38,40.02) | 28.98(14.16,48.75) | 1.42(1.06,1.78) |
| Croatia | male | 1863.75(1004.77,2796.15) | 1083.63(806.98,1407.20) | -0.42 | 80.26(42.95,120.68) | 30.35(22.75,39.07) | -3.49(-3.72,-3.26) |
| Cuba | male | 1856.21(705.59,3317.55) | 1987.23(987.66,3267.44) | 0.07 | 37.32(14.25,66.79) | 22.31(11.12,36.58) | -1.98(-2.26,-1.71) |
| Cyprus | male | 133.23(55.11,221.32) | 137.00(102.72,177.74) | 0.03 | 38.94(16.29,65.03) | 16.16(12.13,21.03) | -3.59(-4.04,-3.15) |
| Czechia | male | 5641.28(3085.43,8290.11) | 2230.16(1681.06,2870.38) | -0.60 | 106.89(57.61,157.61) | 25.55(19.32,32.92) | -4.98(-5.08,-4.87) |
| Democratic Republic of the Congo | male | 793.62(224.45,1951.94) | 2364.65(935.80,4577.91) | 1.98 | 11.98(3.44,29.13) | 16.51(6.70,31.45) | 0.68(0.04,1.33) |
| Denmark | male | 1406.07(584.71,2349.45) | 316.14(193.85,447.34) | -0.78 | 41.49(17.07,69.28) | 6.06(3.74,8.53) | -6.94(-7.19,-6.69) |
| Djibouti | male | 10.77(3.46,23.79) | 134.59(65.25,212.01) | 11.49 | 16.90(5.57,36.50) | 46.98(23.39,72.98) | 4.06(3.59,4.53) |
| Dominica | male | 7.20(2.66,13.77) | 8.83(3.74,15.41) | 0.23 | 24.86(9.20,47.53) | 20.67(8.72,36.13) | -0.95(-1.19,-0.70) |
| Dominican Republic | male | 234.46(70.50,534.84) | 1667.08(752.28,3012.97) | 6.11 | 13.15(3.95,30.13) | 37.74(17.07,68.66) | 4.73(4.38,5.09) |
| Ecuador | male | 513.22(240.01,879.08) | 1280.56(816.01,1819.39) | 1.50 | 19.91(9.23,34.22) | 18.70(12.11,26.44) | 0.20(-0.15,0.54) |
| Egypt | male | 18626.73(14717.53,22928.28) | 41146.46(29498.68,54994.39) | 1.21 | 135.20(105.86,165.26) | 124.18(89.53,163.08) | -0.31(-0.45,-0.17) |
| El Salvador | male | 221.08(81.49,418.05) | 552.13(337.34,849.90) | 1.50 | 16.21(6.01,30.84) | 21.57(13.15,33.21) | 0.97(0.72,1.22) |
| Equatorial Guinea | male | 10.69(2.62,28.80) | 74.97(42.36,113.93) | 6.01 | 12.97(3.31,35.17) | 42.23(24.32,62.89) | 4.61(4.38,4.83) |
| Eritrea | male | 56.18(15.48,141.18) | 311.52(124.40,584.63) | 4.55 | 13.76(3.94,34.33) | 28.80(11.69,53.48) | 2.20(1.90,2.51) |
| Estonia | male | 288.92(109.81,534.24) | 55.84(18.21,101.29) | -0.81 | 42.93(16.23,80.31) | 5.89(1.93,10.69) | -7.23(-8.03,-6.41) |
| Ethiopia | male | 565.30(154.86,1496.43) | 1953.63(902.80,3651.41) | 2.46 | 5.77(1.65,14.88) | 10.12(4.69,18.80) | 2.24(1.89,2.60) |
| Micronesia (Federated States of) | male | 5.53(1.19,15.29) | 13.52(4.02,31.02) | 1.44 | 23.02(5.04,63.27) | 37.99(11.48,87.47) | 1.66(1.47,1.85) |
| Fiji | male | 39.75(9.29,109.92) | 115.37(34.96,248.69) | 1.90 | 21.45(4.99,59.37) | 33.98(10.19,73.82) | 1.27(0.68,1.86) |
| Finland | male | 562.50(63.77,1226.22) | 156.65(31.33,321.24) | -0.72 | 20.95(2.39,45.65) | 2.90(0.58,5.95) | -6.89(-7.42,-6.36) |
| France | male | 7469.85(3342.57,12219.62) | 3390.13(2478.22,4364.22) | -0.55 | 22.97(10.24,37.72) | 5.64(4.16,7.23) | -4.81(-5.03,-4.58) |
| Gabon | male | 72.21(29.12,134.39) | 238.55(145.07,345.92) | 2.30 | 31.01(12.65,56.94) | 53.90(33.12,77.39) | 1.77(1.46,2.09) |
| Georgia | male | 1857.16(970.92,3124.68) | 1394.47(968.54,1861.37) | -0.25 | 86.98(44.63,147.37) | 59.68(41.44,79.52) | -1.52(-1.72,-1.31) |
| Germany | male | 23018.18(11005.24,36292.72) | 8516.29(6283.64,10892.13) | -0.63 | 49.95(23.68,78.76) | 9.88(7.25,12.56) | -5.60(-5.87,-5.34) |
| Ghana | male | 517.36(225.68,924.68) | 2858.71(1815.25,4054.09) | 4.53 | 19.90(8.79,35.11) | 45.06(28.40,63.08) | 3.33(3.09,3.56) |
| Greece | male | 2691.74(1288.54,4306.83) | 1848.43(1414.37,2284.31) | -0.31 | 40.49(19.28,64.73) | 16.92(12.96,20.80) | -3.22(-3.55,-2.88) |
| Greenland | male | 2.36(0.45,6.28) | 2.12(0.17,6.09) | -0.10 | 14.42(2.73,39.14) | 6.23(0.50,17.95) | -3.21(-3.53,-2.89) |
| Grenada | male | 12.66(3.59,27.46) | 15.98(6.35,26.82) | 0.26 | 41.71(11.83,90.28) | 32.82(12.81,55.66) | -1.24(-1.57,-0.91) |
| Guam | male | 7.02(1.10,18.78) | 12.98(5.11,22.43) | 0.85 | 19.21(2.99,51.29) | 14.28(5.65,24.63) | -0.73(-1.49,0.05) |
| Guatemala | male | 214.93(70.41,452.33) | 845.09(490.95,1250.54) | 2.93 | 13.41(4.44,28.09) | 19.02(11.20,27.99) | 0.73(0.30,1.15) |
| Guinea | male | 113.68(34.68,277.73) | 421.41(171.59,822.75) | 2.71 | 7.63(2.41,18.36) | 16.49(6.86,31.64) | 3.22(3.07,3.38) |
| Guinea-Bissau | male | 28.91(8.10,70.87) | 82.44(34.71,155.38) | 1.85 | 16.01(4.63,38.39) | 27.26(11.91,50.44) | 2.10(2.01,2.18) |
| Guyana | male | 138.98(37.95,275.53) | 157.75(67.21,287.69) | 0.14 | 76.52(20.44,152.06) | 55.19(23.11,100.75) | -0.96(-1.16,-0.77) |
| Haiti | male | 180.84(49.13,460.28) | 552.02(196.37,1140.27) | 2.05 | 12.62(3.49,32.15) | 18.15(6.54,37.44) | 1.53(1.36,1.71) |
| Honduras | male | 102.27(31.48,230.47) | 647.28(338.28,1011.45) | 5.33 | 10.80(3.37,24.12) | 25.01(13.10,38.94) | 3.40(3.09,3.71) |
| Hungary | male | 4883.66(2686.47,7422.84) | 2373.02(1824.85,3037.20) | -0.51 | 86.03(46.82,130.24) | 31.99(24.61,40.70) | -3.70(-3.86,-3.53) |
| Iceland | male | 12.78(1.08,31.78) | 5.73(1.16,12.02) | -0.55 | 9.91(0.84,24.59) | 2.18(0.44,4.57) | -5.28(-5.57,-4.99) |
| India | male | 66733.67(31191.73,113053.84) | 303369.55(222976.98,386022.06) | 3.55 | 31.31(14.79,52.59) | 56.86(42.10,72.20) | 2.41(2.25,2.57) |
| Indonesia | male | 11539.94(4872.69,20802.50) | 42531.01(29407.06,58305.45) | 2.69 | 25.71(10.74,46.74) | 44.67(31.44,59.71) | 1.90(1.68,2.13) |
| Iran (Islamic Republic of) | male | 10036.55(8138.41,11863.67) | 17628.67(15196.31,20088.87) | 0.76 | 84.82(69.08,100.39) | 50.74(43.48,57.83) | -2.08(-2.34,-1.83) |
| Iraq | male | 4108.18(2784.22,5587.11) | 11689.11(8686.58,14804.81) | 1.85 | 113.17(77.02,153.21) | 115.57(87.62,143.68) | -0.03(-0.24,0.18) |
| Ireland | male | 577.26(173.41,1063.60) | 164.39(84.34,252.66) | -0.72 | 32.72(9.78,60.44) | 4.83(2.48,7.42) | -6.98(-7.22,-6.73) |
| Israel | male | 876.81(468.19,1329.69) | 589.27(480.26,698.39) | -0.33 | 40.30(21.58,61.22) | 11.18(9.16,13.24) | -5.02(-5.23,-4.80) |
| Italy | male | 13939.87(7580.63,20717.21) | 6831.22(5436.87,8337.04) | -0.51 | 39.13(21.27,58.01) | 10.49(8.40,12.74) | -4.69(-4.81,-4.58) |
| Jamaica | male | 119.89(52.20,218.93) | 244.12(150.51,358.38) | 1.04 | 14.63(6.36,26.67) | 17.32(10.70,25.42) | 0.71(0.28,1.14) |
| Japan | male | 10401.17(3384.76,19123.44) | 10779.19(7420.71,14662.65) | 0.04 | 15.65(5.18,28.95) | 6.99(4.85,9.45) | -2.89(-3.18,-2.60) |
| Jordan | male | 409.52(314.00,510.27) | 1233.00(930.10,1618.09) | 2.01 | 65.46(50.85,81.65) | 39.43(30.16,51.09) | -2.06(-2.25,-1.88) |
| Kazakhstan | male | 2987.93(1499.18,4993.22) | 3984.03(2624.19,5640.19) | 0.33 | 68.83(34.60,115.23) | 66.08(43.91,91.76) | -0.76(-1.28,-0.24) |
| Kenya | male | 246.51(102.35,483.66) | 1494.85(813.98,2367.85) | 5.06 | 6.69(2.81,13.19) | 16.29(8.83,25.65) | 3.75(3.29,4.22) |
| Kiribati | male | 3.88(0.93,10.48) | 8.52(2.47,21.02) | 1.19 | 20.87(5.01,56.60) | 25.32(7.40,62.28) | -0.22(-0.52,0.08) |
| Kuwait | male | 273.53(238.03,308.84) | 901.24(714.63,1139.16) | 2.29 | 69.46(59.78,78.46) | 56.03(44.78,70.31) | -0.34(-0.82,0.13) |
| Kyrgyzstan | male | 551.45(239.64,987.04) | 1204.30(755.22,1716.45) | 1.18 | 49.50(21.71,88.52) | 69.06(43.17,99.98) | 1.04(0.62,1.46) |
| Lao People's Democratic Republic | male | 124.84(33.27,301.70) | 508.24(248.11,838.81) | 3.07 | 13.80(3.79,32.30) | 26.24(12.87,42.60) | 2.23(1.96,2.50) |
| Latvia | male | 1077.94(560.10,1654.69) | 415.85(282.34,587.57) | -0.61 | 89.32(45.74,137.70) | 29.73(20.26,42.01) | -4.01(-4.65,-3.37) |
| Lebanon | male | 773.10(544.78,1021.97) | 1519.99(1089.78,1935.46) | 0.97 | 73.87(52.19,96.65) | 65.14(46.60,83.01) | -0.01(-0.32,0.30) |
| Lesotho | male | 58.74(24.22,115.17) | 189.40(99.43,297.23) | 2.22 | 15.47(6.54,29.85) | 38.56(20.33,60.26) | 3.58(3.27,3.90) |
| Liberia | male | 58.48(19.50,131.96) | 155.90(67.28,290.41) | 1.67 | 10.96(3.73,24.52) | 16.67(7.44,30.56) | 2.50(2.05,2.95) |
| Libya | male | 459.62(286.44,668.33) | 1410.21(970.81,2056.40) | 2.07 | 48.75(30.30,69.50) | 55.91(38.41,79.87) | 0.20(-0.08,0.48) |
| Lithuania | male | 1045.29(487.51,1709.73) | 492.05(318.10,704.45) | -0.53 | 62.44(29.24,102.49) | 23.98(15.55,34.20) | -3.04(-3.64,-2.44) |
| Luxembourg | male | 78.60(31.05,134.37) | 26.81(17.56,37.32) | -0.66 | 37.46(14.94,63.83) | 5.98(3.91,8.31) | -6.32(-6.59,-6.04) |
| North Macedonia | male | 864.28(521.19,1196.06) | 1138.43(877.82,1442.04) | 0.32 | 107.31(64.15,148.83) | 87.69(69.17,109.36) | -1.03(-1.42,-0.64) |
| Madagascar | male | 175.92(59.59,394.54) | 675.82(275.04,1343.29) | 2.84 | 7.03(2.39,15.61) | 13.67(5.74,26.49) | 2.66(2.48,2.83) |
| Malawi | male | 82.96(23.92,219.20) | 308.96(124.65,622.85) | 2.72 | 5.11(1.50,13.30) | 10.51(4.21,21.15) | 3.07(2.80,3.34) |
| Malaysia | male | 2735.82(1312.51,4159.99) | 4273.20(2932.45,5992.39) | 0.56 | 62.80(29.99,95.84) | 32.62(22.51,45.07) | -2.33(-2.45,-2.20) |
| Maldives | male | 9.44(2.90,21.45) | 23.20(14.76,33.51) | 1.46 | 19.76(6.19,44.30) | 14.28(8.95,20.83) | -1.95(-2.31,-1.60) |
| Mali | male | 119.13(29.15,312.85) | 436.23(147.68,906.75) | 2.66 | 7.40(1.91,19.52) | 11.95(4.05,24.51) | 2.12(1.90,2.35) |
| Malta | male | 69.72(28.90,117.34) | 50.57(36.93,65.12) | -0.27 | 39.39(16.22,66.14) | 12.34(9.09,15.84) | -3.76(-4.13,-3.40) |
| Marshall Islands | male | 1.52(0.33,4.07) | 5.45(1.86,11.22) | 2.60 | 18.43(4.00,49.52) | 28.85(9.83,60.28) | 1.56(1.32,1.80) |
| Mauritania | male | 105.12(40.98,196.54) | 288.39(162.77,426.04) | 1.74 | 24.49(9.70,44.90) | 30.43(17.28,44.57) | 0.61(0.32,0.90) |
| Mauritius | male | 176.26(93.40,275.06) | 171.63(84.13,267.94) | -0.03 | 52.91(27.73,82.51) | 22.70(11.08,35.24) | -3.83(-4.34,-3.33) |
| Mexico | male | 4857.63(2556.75,7466.92) | 10241.88(7326.66,13462.33) | 1.11 | 25.36(13.31,39.48) | 19.41(13.88,25.43) | -1.24(-1.51,-0.96) |
| Republic of Moldova | male | 1047.28(499.59,1720.70) | 921.04(525.88,1355.09) | -0.12 | 69.11(32.75,113.56) | 40.88(23.37,60.21) | -1.79(-2.35,-1.23) |
| Mongolia | male | 249.46(107.83,441.76) | 1178.78(803.21,1629.87) | 3.73 | 59.35(25.84,104.58) | 127.81(88.93,172.49) | 2.39(2.00,2.79) |
| Montenegro | male | 191.52(111.34,277.45) | 235.00(177.14,295.99) | 0.23 | 76.18(44.25,110.64) | 57.21(43.33,71.90) | -0.92(-1.11,-0.74) |
| Morocco | male | 2892.73(1496.18,4485.88) | 11906.96(8597.10,15145.79) | 3.12 | 46.55(24.56,72.24) | 85.92(63.32,108.82) | 1.80(1.59,2.01) |
| Mozambique | male | 94.24(23.36,265.92) | 533.90(219.52,1118.23) | 4.67 | 3.68(0.94,10.19) | 11.68(4.72,24.87) | 5.24(4.84,5.63) |
| Myanmar | male | 2503.98(837.50,5500.03) | 8084.87(4784.38,11598.87) | 2.23 | 24.33(8.36,52.80) | 43.16(25.53,61.23) | 2.42(2.12,2.73) |
| Namibia | male | 72.06(32.49,123.10) | 217.18(130.20,320.24) | 2.01 | 23.87(10.88,40.67) | 40.28(24.13,58.84) | 1.89(1.60,2.19) |
| Nepal | male | 586.08(162.82,1377.21) | 4505.30(2667.28,6581.68) | 6.69 | 13.53(3.83,31.43) | 45.95(27.39,66.78) | 4.90(4.57,5.22) |
| Netherlands | male | 3010.44(1428.31,4791.30) | 1061.80(776.53,1362.90) | -0.65 | 37.13(17.69,59.28) | 6.95(5.10,8.84) | -6.24(-6.49,-6.00) |
| New Zealand | male | 175.44(10.05,446.72) | 96.30(17.25,196.33) | -0.45 | 10.40(0.58,26.58) | 2.69(0.48,5.45) | -5.40(-5.68,-5.12) |
| Nicaragua | male | 55.55(18.34,119.84) | 349.17(187.38,553.27) | 5.29 | 9.15(3.04,19.57) | 20.76(11.31,32.95) | 2.78(2.46,3.10) |
| Niger | male | 105.76(22.81,301.06) | 366.69(97.58,909.60) | 2.47 | 8.76(1.99,24.54) | 11.57(3.23,28.06) | 1.18(1.01,1.35) |
| Nigeria | male | 3197.93(1299.67,7064.93) | 10938.64(6253.11,16853.80) | 2.42 | 16.90(7.14,35.74) | 30.47(17.97,46.79) | 2.10(1.87,2.33) |
| Democratic People's Republic of Korea | male | 1728.32(637.95,3176.95) | 7342.61(4451.94,10323.83) | 3.25 | 32.52(12.37,59.28) | 61.14(37.25,84.46) | 2.48(2.35,2.61) |
| Northern Mariana Islands | male | 2.51(0.74,6.05) | 4.40(2.25,7.11) | 0.75 | 20.06(5.99,48.30) | 16.52(8.51,26.64) | -0.41(-1.02,0.20) |
| Norway | male | 704.68(183.18,1350.17) | 108.53(39.83,191.21) | -0.85 | 24.40(6.36,46.97) | 2.45(0.91,4.32) | -8.19(-8.50,-7.87) |
| Oman | male | 319.16(175.55,501.18) | 722.36(545.42,934.52) | 1.26 | 101.36(56.48,160.66) | 103.02(79.40,129.67) | -0.15(-0.59,0.28) |
| Pakistan | male | 5479.70(1843.75,11450.43) | 33947.88(21850.33,49724.21) | 5.20 | 18.25(6.20,38.08) | 60.96(39.42,87.39) | 4.58(4.23,4.92) |
| Palestine | male | 222.34(110.86,351.68) | 673.89(498.29,846.33) | 2.03 | 61.35(31.04,96.92) | 70.88(52.78,88.65) | -0.04(-0.50,0.42) |
| Panama | male | 124.71(57.53,226.20) | 209.92(121.25,315.67) | 0.68 | 17.27(7.92,31.45) | 10.58(6.13,15.96) | -1.26(-1.52,-1.01) |
| Papua New Guinea | male | 80.92(14.60,260.95) | 357.15(80.73,905.19) | 3.41 | 8.75(1.59,28.01) | 14.15(3.32,36.32) | 1.70(1.51,1.88) |
| Paraguay | male | 139.05(58.79,255.76) | 379.14(233.92,586.42) | 1.73 | 13.84(5.88,25.55) | 14.82(9.11,22.99) | 0.13(-0.09,0.36) |
| Peru | male | 1414.55(655.04,2322.94) | 2246.67(1477.29,3259.36) | 0.59 | 25.31(11.68,41.69) | 14.51(9.53,21.05) | -1.64(-2.12,-1.17) |
| Philippines | male | 2194.74(1058.89,3748.48) | 12688.05(8561.12,17924.05) | 4.78 | 17.48(8.49,29.78) | 34.46(23.41,47.92) | 2.72(1.86,3.58) |
| Poland | male | 16359.32(9484.26,23292.01) | 9661.98(7408.22,12603.41) | -0.41 | 97.51(56.35,138.93) | 34.85(26.71,45.17) | -3.96(-4.13,-3.79) |
| Portugal | male | 1493.56(459.04,2806.39) | 538.33(314.84,775.86) | -0.64 | 27.32(8.43,51.63) | 5.17(3.01,7.47) | -6.24(-6.59,-5.88) |
| Puerto Rico | male | 149.86(0.87,498.92) | 99.06(20.66,185.94) | -0.34 | 9.19(0.05,30.44) | 3.22(0.66,6.02) | -4.39(-4.99,-3.79) |
| Qatar | male | 83.51(65.41,105.94) | 275.80(199.74,371.87) | 2.30 | 149.12(118.88,183.46) | 68.45(52.36,87.13) | -2.89(-3.27,-2.50) |
| Romania | male | 7410.41(3815.52,11384.65) | 5884.64(4540.14,7489.99) | -0.21 | 66.67(34.47,102.90) | 38.15(29.49,48.48) | -2.67(-3.06,-2.27) |
| Russian Federation | male | 44641.83(19323.70,72961.83) | 30451.45(16999.93,44895.81) | -0.32 | 82.57(34.82,136.65) | 36.33(20.12,53.48) | -3.05(-3.74,-2.36) |
| Rwanda | male | 179.48(51.66,420.93) | 384.77(155.94,753.08) | 1.14 | 14.72(4.35,34.15) | 17.54(7.15,33.95) | 0.01(-0.36,0.39) |
| Saint Lucia | male | 12.50(4.39,25.19) | 23.87(10.59,40.36) | 0.91 | 35.90(12.37,72.48) | 24.92(10.96,42.22) | -1.91(-2.28,-1.53) |
| Saint Vincent and the Grenadines | male | 9.15(2.69,19.39) | 20.01(8.01,34.65) | 1.19 | 30.74(9.08,65.49) | 30.72(12.37,53.36) | -0.47(-0.77,-0.18) |
| Samoa | male | 9.67(2.37,24.62) | 16.16(4.73,35.64) | 0.67 | 23.55(5.91,58.83) | 22.80(6.68,49.89) | -0.33(-0.44,-0.22) |
| Sao Tome and Principe | male | 2.57(0.97,5.37) | 13.89(7.26,22.56) | 4.40 | 10.11(3.90,20.94) | 30.88(16.39,50.57) | 4.55(4.27,4.84) |
| Saudi Arabia | male | 1933.25(1052.35,3031.85) | 9659.58(7530.90,12124.82) | 4.00 | 62.88(35.18,96.96) | 92.71(75.18,111.64) | 1.02(0.43,1.62) |
| Senegal | male | 216.98(72.44,480.30) | 757.06(355.62,1320.62) | 2.49 | 14.89(5.17,32.64) | 23.42(11.19,40.48) | 1.40(1.21,1.59) |
| Serbia | male | 3975.25(2270.23,5729.66) | 3895.32(3013.91,4931.54) | -0.02 | 84.04(47.90,121.10) | 58.32(45.56,72.86) | -1.45(-1.83,-1.06) |
| Seychelles | male | 9.49(5.15,14.96) | 12.95(6.94,19.61) | 0.36 | 39.49(21.49,62.91) | 25.23(13.26,38.70) | -2.11(-2.33,-1.88) |
| Sierra Leone | male | 104.39(32.05,257.25) | 277.98(108.39,565.91) | 1.66 | 11.46(3.62,27.76) | 17.09(6.81,33.81) | 1.87(1.58,2.15) |
| Singapore | male | 393.03(136.90,679.66) | 418.16(272.44,559.60) | 0.06 | 41.59(14.23,72.83) | 11.64(7.53,15.59) | -4.53(-4.86,-4.20) |
| Slovakia | male | 2501.19(1380.94,3671.80) | 1349.74(993.10,1770.27) | -0.46 | 103.49(57.01,151.72) | 37.70(28.02,48.78) | -3.53(-3.76,-3.30) |
| Slovenia | male | 475.79(248.30,757.01) | 261.61(190.01,352.38) | -0.45 | 52.03(27.04,83.03) | 14.38(10.50,19.31) | -5.06(-5.29,-4.84) |
| Solomon Islands | male | 9.26(1.62,29.75) | 48.00(12.40,125.71) | 4.18 | 11.87(2.11,37.39) | 28.63(7.43,74.08) | 2.92(2.79,3.04) |
| Somalia | male | 43.95(8.93,136.58) | 124.53(26.95,380.06) | 1.83 | 4.00(0.84,12.17) | 4.81(1.10,14.15) | 1.10(0.83,1.37) |
| South Africa | male | 2550.83(1932.94,3233.29) | 5395.01(4334.91,6633.27) | 1.11 | 28.49(21.35,36.33) | 31.15(24.85,38.15) | 0.31(-0.20,0.82) |
| Republic of Korea | male | 7459.69(4120.37,11069.97) | 5559.25(4499.23,7072.09) | -0.25 | 71.38(38.76,107.27) | 15.72(12.74,19.74) | -5.48(-5.84,-5.13) |
| South Sudan | male | 113.15(37.21,250.11) | 231.01(90.79,440.92) | 1.04 | 9.44(3.17,21.00) | 12.98(5.12,24.43) | 1.45(1.28,1.61) |
| Spain | male | 4548.02(1725.87,7965.49) | 2191.32(1478.98,2972.65) | -0.52 | 20.89(7.98,36.73) | 5.12(3.47,6.90) | -4.88(-5.19,-4.57) |
| Sri Lanka | male | 1390.60(598.67,2500.78) | 2604.99(1652.48,3865.31) | 0.87 | 28.26(12.04,50.62) | 25.36(16.23,37.21) | 0.05(-0.49,0.59) |
| Sudan | male | 1264.14(434.65,2764.95) | 7310.28(4315.11,11465.66) | 4.78 | 27.93(9.78,60.37) | 77.79(46.40,119.19) | 3.79(3.69,3.90) |
| Suriname | male | 52.77(19.16,94.65) | 99.54(47.40,166.87) | 0.89 | 43.97(15.91,78.88) | 36.55(17.37,61.28) | -0.84(-1.26,-0.41) |
| Eswatini | male | 21.61(9.70,38.01) | 81.30(45.76,124.06) | 2.76 | 19.04(8.72,33.14) | 37.65(21.62,56.02) | 2.55(2.19,2.91) |
| Sweden | male | 1190.25(205.52,2475.31) | 219.47(58.16,437.69) | -0.82 | 18.19(3.17,37.64) | 2.17(0.57,4.32) | -7.37(-7.75,-6.98) |
| Switzerland | male | 1247.34(542.23,2058.32) | 405.76(268.12,551.72) | -0.67 | 29.54(12.68,48.71) | 5.00(3.32,6.76) | -6.14(-6.32,-5.95) |
| Syrian Arab Republic | male | 2804.11(2001.52,3735.86) | 5320.82(3775.27,7272.08) | 0.90 | 103.48(73.95,137.65) | 91.22(66.08,122.82) | -0.76(-0.95,-0.57) |
| Taiwan (Province of China) | male | 2408.47(1312.71,3787.43) | 2986.91(2278.85,3939.61) | 0.24 | 31.69(17.33,49.70) | 16.51(12.62,21.71) | -2.30(-2.47,-2.12) |
| Tajikistan | male | 468.30(159.37,932.27) | 2039.29(1073.72,3134.53) | 3.35 | 40.40(13.77,80.81) | 115.39(60.83,178.05) | 4.02(3.78,4.26) |
| United Republic of Tanzania | male | 288.67(100.11,653.27) | 1402.41(644.21,2608.08) | 3.86 | 6.03(2.14,13.55) | 13.62(6.28,24.89) | 3.09(2.95,3.23) |
| Thailand | male | 4987.29(2385.92,7921.83) | 10448.04(7517.94,14573.64) | 1.09 | 32.97(15.78,52.24) | 23.22(16.86,32.04) | -1.85(-2.12,-1.58) |
| Bahamas | male | 21.58(5.61,41.61) | 36.98(10.48,70.06) | 0.71 | 33.35(8.53,64.59) | 21.26(5.97,40.28) | -1.90(-2.12,-1.69) |
| Gambia | male | 18.74(5.49,45.45) | 118.04(56.85,200.39) | 5.30 | 11.98(3.75,28.20) | 28.15(14.16,47.92) | 3.07(2.92,3.21) |
| Timor-Leste | male | 6.04(1.41,16.85) | 76.28(31.78,138.53) | 11.63 | 4.90(1.16,13.57) | 20.65(8.76,37.30) | 6.40(5.71,7.10) |
| Togo | male | 76.46(27.82,162.55) | 396.97(184.42,697.15) | 4.19 | 14.77(5.50,31.01) | 28.44(13.52,48.14) | 2.31(2.07,2.56) |
| Tonga | male | 3.18(0.84,8.15) | 6.42(2.09,13.92) | 1.02 | 12.51(3.32,31.86) | 17.89(5.76,38.75) | 1.03(0.68,1.39) |
| Trinidad and Tobago | male | 216.64(45.02,431.50) | 278.75(94.05,509.36) | 0.29 | 57.41(11.54,115.99) | 32.55(11.06,59.30) | -2.60(-2.93,-2.26) |
| Tunisia | male | 1384.72(907.20,1894.80) | 3302.23(2187.76,4616.89) | 1.38 | 60.37(39.89,82.45) | 58.73(39.53,81.24) | -0.34(-0.67,-0.01) |
| Turkey | male | 9534.56(7047.78,11756.79) | 13632.38(10182.77,17726.15) | 0.43 | 56.63(42.22,70.05) | 34.49(25.89,44.65) | -1.71(-1.90,-1.52) |
| Turkmenistan | male | 735.02(307.91,1226.12) | 1740.29(1048.46,2567.27) | 1.37 | 101.84(42.78,170.65) | 109.59(65.72,162.11) | -0.44(-0.83,-0.04) |
| Uganda | male | 171.14(49.64,416.80) | 906.29(417.04,1633.72) | 4.30 | 6.04(1.78,14.73) | 16.30(7.59,29.23) | 3.87(3.61,4.13) |
| Ukraine | male | 19078.09(9194.41,29960.36) | 19583.02(11488.32,28619.99) | 0.03 | 85.16(40.35,135.31) | 71.77(42.69,104.79) | -0.99(-1.43,-0.54) |
| United Arab Emirates | male | 304.11(218.87,430.77) | 2041.91(1341.11,3036.61) | 5.71 | 116.35(86.39,158.86) | 67.23(45.62,93.48) | -2.39(-2.75,-2.03) |
| United Kingdom | male | 14762.35(6122.83,24531.57) | 4015.90(2664.73,5482.32) | -0.73 | 40.35(16.90,66.96) | 7.05(4.69,9.59) | -6.55(-6.76,-6.33) |
| United States of America | male | 33510.65(13033.07,60365.66) | 14434.99(7708.22,21824.52) | -0.57 | 25.56(9.93,45.84) | 5.85(3.13,8.83) | -5.60(-5.87,-5.32) |
| Uruguay | male | 337.95(103.60,693.32) | 199.26(97.26,315.80) | -0.41 | 20.68(6.35,42.23) | 8.88(4.33,14.13) | -3.15(-3.36,-2.94) |
| Uzbekistan | male | 3361.07(1404.49,5577.60) | 12541.30(8020.67,17511.10) | 2.73 | 77.59(31.92,130.07) | 184.22(117.51,253.82) | 2.73(2.04,3.42) |
| Vanuatu | male | 5.62(1.20,15.19) | 25.78(7.30,59.74) | 3.58 | 15.48(3.33,41.13) | 28.29(7.98,65.68) | 1.72(1.53,1.92) |
| Venezuela (Bolivarian Republic of) | male | 1773.12(795.91,2913.58) | 4770.38(3073.40,7033.71) | 1.69 | 39.49(17.80,65.25) | 36.06(23.05,53.13) | -0.54(-0.82,-0.25) |
| Viet nam | male | 3412.82(1244.08,6999.97) | 16825.63(11644.95,22319.36) | 3.93 | 22.20(8.11,45.21) | 46.95(33.11,61.77) | 3.14(2.59,3.70) |
| Virginia | male | 796.00(340.33,1390.05) | 267.43(94.46,471.51) | -0.66 | 28.10(11.88,49.02) | 4.32(1.54,7.59) | -6.83(-7.23,-6.44) |
| Yemen | male | 573.85(155.75,1442.85) | 4603.01(2310.71,7380.25) | 7.02 | 27.04(7.53,67.29) | 75.20(37.03,119.25) | 3.96(3.72,4.21) |
| Zambia | male | 133.67(48.43,282.12) | 836.12(419.93,1369.50) | 5.26 | 9.86(3.60,20.65) | 28.65(14.55,46.61) | 3.96(3.81,4.11) |
| Zimbabwe | male | 212.15(92.42,386.16) | 505.18(244.75,854.55) | 1.38 | 12.69(5.66,22.76) | 19.30(9.28,32.12) | 1.06(0.55,1.57) |
| Monaco | male | 4.58(0.45,9.86) | 3.77(2.17,5.61) | -0.18 | 15.42(1.52,33.11) | 8.51(4.91,12.66) | -1.74(-2.37,-1.10) |
| San Marino | male | 1.66(0.37,3.43) | 1.63(0.64,2.97) | -0.02 | 11.87(2.61,24.63) | 5.29(2.08,9.68) | -3.26(-3.74,-2.78) |
| Saint Kitts and Nevis | male | 4.47(1.50,8.95) | 4.02(1.77,6.67) | -0.10 | 28.49(9.46,57.53) | 13.72(6.07,22.47) | -3.29(-3.78,-2.80) |
| Cook Islands | male | 0.75(0.27,1.80) | 0.83(0.15,1.95) | 0.11 | 11.25(4.07,27.49) | 7.21(1.28,17.35) | -1.66(-1.99,-1.33) |
| Nauru | male | 0.60(0.17,1.70) | 0.52(0.12,1.21) | -0.14 | 25.47(7.17,73.20) | 21.10(4.89,49.78) | -1.24(-1.51,-0.98) |
| Niue | male | 0.17(0.07,0.39) | 0.15(0.03,0.35) | -0.13 | 18.69(7.23,42.14) | 15.44(2.83,35.78) | -1.22(-1.49,-0.96) |
| Palau | male | 0.95(0.01,2.94) | 1.76(0.01,4.47) | 0.85 | 18.65(0.16,57.59) | 15.61(0.05,39.22) | 0.26(-0.58,1.11) |
| Tokelau | male | 0.08(0.00,0.30) | 0.06(0.00,0.16) | -0.29 | 13.45(0.33,50.12) | 8.79(0.03,24.34) | -1.88(-2.19,-1.56) |
| Tuvalu | male | 0.38(0.09,1.10) | 0.78(0.28,1.71) | 1.06 | 12.96(3.23,37.39) | 16.08(5.84,35.23) | 0.38(0.16,0.59) |

**Supplementary Table 4.** The DALY of global CVD burden due to ambient particulate matter pollution, and its temporal trends from 1990 to 2019.

| Nation | Sex | DALY Cases No. (95% UI) | | Change in absolute number (%) | Age-standardized DALY rate per 100,000 No.(95% UI) | | 1990-2019 EAPC No. (95%CI) |
| --- | --- | --- | --- | --- | --- | --- | --- |
|  |  | 1990 | 2019 |  | 1990 | 2019 |  |
| Afghanistan | both | 35441.84(8669.11,95228.10) | 150222.25(64449.87,288982.33) | 3.24 | 480.95(118.89,1285.53) | 964.59(419.41,1821.70) | 2.69(2.14,3.24) |
| Albania | both | 12367.69(5876.68,20046.91) | 23833.01(16837.97,32872.89) | 0.93 | 597.39(286.44,973.55) | 578.29(408.46,798.32) | 0.45(0.13,0.77) |
| Algeria | both | 214812.48(139324.01,304351.34) | 408593.14(278758.41,553197.29) | 0.90 | 1734.68(1123.84,2417.25) | 1196.07(822.77,1614.79) | -1.71(-1.93,-1.49) |
| American Samoa | both | 84.89(27.27,216.21) | 131.35(50.63,259.86) | 0.55 | 314.04(101.48,806.51) | 260.46(101.52,510.65) | -0.93(-1.20,-0.66) |
| Andorra | both | 119.74(38.60,222.10) | 98.45(52.84,153.34) | -0.18 | 216.63(70.04,401.05) | 71.30(38.20,110.88) | -3.74(-3.91,-3.57) |
| Angola | both | 7500.89(2157.92,18361.23) | 67137.18(33983.86,114540.93) | 7.95 | 172.57(51.13,420.43) | 542.63(274.39,916.31) | 4.03(3.75,4.30) |
| Antigua and Barbuda | both | 333.16(91.75,651.11) | 394.51(151.28,677.91) | 0.18 | 642.08(177.28,1250.94) | 385.39(147.96,660.68) | -2.11(-2.41,-1.81) |
| Argentina | both | 192185.89(76835.69,339629.52) | 138046.99(87659.65,196246.84) | -0.28 | 598.88(238.66,1058.98) | 263.64(167.56,375.13) | -3.04(-3.34,-2.74) |
| Armenia | both | 35337.73(17328.59,55913.41) | 46009.86(31747.98,60457.84) | 0.30 | 1324.58(646.99,2107.14) | 1132.66(780.44,1485.87) | -0.71(-0.95,-0.47) |
| Australia | both | 34967.28(4061.18,82391.16) | 17165.37(4342.87,30928.69) | -0.51 | 180.53(21.01,426.80) | 44.03(11.11,79.41) | -5.29(-5.62,-4.96) |
| Austria | both | 76545.47(38533.70,119220.32) | 24883.91(18588.15,31670.24) | -0.67 | 663.69(336.29,1027.63) | 144.92(108.64,183.60) | -5.63(-5.82,-5.44) |
| Azerbaijan | both | 59707.01(27888.81,103347.63) | 155368.77(94612.14,225483.12) | 1.60 | 1156.75(540.94,2003.73) | 1716.85(1050.25,2482.94) | 1.17(0.87,1.47) |
| Bahrain | both | 6266.16(5090.62,7513.57) | 11161.70(8850.54,13968.47) | 0.78 | 2935.64(2381.12,3492.33) | 1018.31(819.57,1264.38) | -4.13(-4.40,-3.85) |
| Bangladesh | both | 166873.37(50539.24,372046.95) | 1166849.24(718340.60,1681705.75) | 5.99 | 326.10(101.61,725.11) | 863.18(536.13,1238.76) | 3.80(3.64,3.97) |
| Barbados | both | 1904.66(563.07,3474.61) | 1918.35(851.31,3167.12) | 0.01 | 682.06(199.52,1234.41) | 405.99(180.37,668.80) | -2.22(-2.57,-1.87) |
| Belarus | both | 189105.35(99907.81,285058.74) | 156181.56(107838.89,214894.92) | -0.17 | 1484.53(788.35,2236.34) | 1009.97(697.45,1387.84) | -1.49(-2.15,-0.82) |
| Belgium | both | 87168.96(41905.58,137670.18) | 29562.19(22449.88,37021.61) | -0.66 | 589.51(285.99,923.67) | 138.82(105.18,174.00) | -5.07(-5.26,-4.87) |
| Belize | both | 330.16(76.82,740.31) | 1261.86(497.39,2175.38) | 2.82 | 347.38(80.61,774.96) | 422.62(166.52,734.04) | -0.40(-1.03,0.24) |
| Benin | both | 3886.10(1253.44,8993.47) | 18380.56(8300.67,33226.34) | 3.73 | 190.06(62.31,441.35) | 354.81(162.79,638.94) | 2.11(1.81,2.41) |
| Bermuda | both | 254.29(58.87,645.46) | 93.48(16.59,183.50) | -0.63 | 402.06(93.08,1020.43) | 76.54(13.73,149.43) | -6.24(-6.76,-5.73) |
| Bhutan | both | 422.93(110.45,1030.59) | 3231.66(1895.60,4867.86) | 6.64 | 154.97(42.30,372.16) | 548.99(323.75,819.46) | 5.41(5.05,5.76) |
| Bolivia (Plurinational State of) | both | 22814.11(8233.86,43010.29) | 44305.84(24994.17,67520.25) | 0.94 | 659.70(239.22,1234.90) | 487.34(276.84,741.07) | -1.26(-1.70,-0.82) |
| Bosnia and Herzegovina | both | 36532.45(18229.36,56943.01) | 50735.39(38048.43,64604.36) | 0.39 | 899.27(446.16,1407.60) | 881.38(659.04,1122.20) | -0.14(-0.46,0.18) |
| Botswana | both | 2388.42(1114.10,4224.22) | 12088.20(7421.15,18138.81) | 4.06 | 395.72(188.38,696.13) | 812.41(511.71,1199.23) | 2.15(1.46,2.85) |
| Brazil | both | 523303.73(239129.34,911566.79) | 620330.84(437588.03,815071.39) | 0.19 | 531.67(242.12,929.18) | 256.34(181.16,336.39) | -2.61(-2.85,-2.38) |
| Brunei Darussalam | both | 481.04(179.33,946.00) | 608.97(182.93,1069.89) | 0.27 | 404.82(152.31,803.37) | 177.49(52.05,314.15) | -2.61(-3.39,-1.82) |
| Bulgaria | both | 222182.15(124610.53,329305.11) | 146804.15(112318.45,186467.61) | -0.34 | 1862.77(1047.27,2753.32) | 1092.61(834.33,1405.66) | -2.67(-3.07,-2.27) |
| Burkina Faso | both | 5097.09(1290.71,13071.76) | 23729.79(8171.59,50687.54) | 3.66 | 113.19(28.91,288.19) | 242.34(84.57,517.12) | 3.23(3.01,3.44) |
| Burundi | both | 5224.92(1399.36,13854.41) | 9850.55(3172.50,23161.79) | 0.89 | 207.75(56.84,544.28) | 196.13(65.46,455.00) | -0.49(-0.82,-0.15) |
| Cambodia | both | 10078.76(2885.15,25136.52) | 47688.00(22659.56,82358.11) | 3.73 | 208.31(60.45,516.57) | 384.91(184.29,658.58) | 2.43(2.21,2.65) |
| Cameroon | both | 17793.89(7699.91,32549.06) | 105128.46(59605.22,157575.73) | 4.91 | 379.85(167.25,685.52) | 798.94(456.72,1167.52) | 2.64(2.44,2.85) |
| Canada | both | 83034.18(21324.73,164876.44) | 37657.13(17804.37,59922.54) | -0.55 | 259.36(66.55,515.71) | 59.24(27.99,94.75) | -5.60(-5.87,-5.33) |
| Cabo Verde | both | 577.80(242.45,1087.66) | 4087.62(2779.30,5330.20) | 6.07 | 257.07(106.79,483.87) | 937.65(636.73,1225.02) | 4.03(3.57,4.50) |
| Central African Republic | both | 3395.09(846.93,8319.76) | 9164.86(2753.85,21303.85) | 1.70 | 261.89(67.31,639.81) | 368.10(112.40,826.38) | 1.19(0.96,1.42) |
| Chad | both | 3721.51(941.93,10351.37) | 15784.61(5669.06,35091.69) | 3.24 | 129.65(33.43,358.36) | 263.07(95.90,576.84) | 2.74(2.59,2.89) |
| Chile | both | 53311.59(26981.41,86663.62) | 72256.75(58449.20,86704.06) | 0.36 | 527.67(266.29,860.26) | 306.01(247.96,367.35) | -1.71(-1.84,-1.58) |
| China | both | 5840517.08(2739167.71,9841164.36) | 20947112.11(16996001.90,25029622.28) | 2.59 | 681.10(323.05,1146.44) | 1066.22(866.63,1268.99) | 1.90(1.56,2.25) |
| Colombia | both | 106586.57(50766.97,183654.83) | 167774.23(115624.51,231698.23) | 0.57 | 564.38(269.20,974.24) | 318.21(218.98,439.02) | -2.27(-2.43,-2.12) |
| Comoros | both | 260.43(82.49,613.44) | 1092.59(514.73,1942.05) | 3.20 | 113.44(37.66,264.90) | 216.25(103.32,382.46) | 1.99(1.80,2.18) |
| Congo | both | 5983.47(2170.84,12162.69) | 26589.59(14237.00,42751.64) | 3.44 | 517.43(189.39,1053.01) | 930.49(506.14,1496.40) | 1.88(1.59,2.17) |
| Costa Rica | both | 5600.22(2667.54,9746.93) | 13417.71(9170.44,18499.17) | 1.40 | 306.28(144.73,535.38) | 258.94(177.78,355.92) | -0.82(-1.04,-0.59) |
| Côte d'Ivoire | both | 14291.72(4876.71,32027.29) | 62658.22(30009.69,106401.97) | 3.38 | 316.50(110.50,704.41) | 524.31(255.96,886.29) | 1.62(1.27,1.97) |
| Croatia | both | 80867.88(44055.88,119470.99) | 40036.56(30087.26,51317.75) | -0.50 | 1291.06(700.15,1910.52) | 474.48(355.96,608.70) | -3.68(-3.90,-3.45) |
| Cuba | both | 75837.99(29235.39,134647.53) | 74694.14(37544.81,122099.35) | -0.02 | 734.92(282.39,1303.29) | 405.87(204.31,662.56) | -2.22(-2.47,-1.96) |
| Cyprus | both | 5252.23(2158.27,8756.30) | 4779.46(3625.84,6077.84) | -0.09 | 682.11(282.16,1141.32) | 258.87(196.91,330.05) | -3.91(-4.22,-3.60) |
| Czechia | both | 226978.32(123532.63,335952.95) | 78594.58(59661.87,99972.28) | -0.65 | 1671.19(916.93,2473.98) | 388.44(294.31,498.43) | -5.14(-5.25,-5.02) |
| Democratic Republic of the Congo | both | 36249.57(10201.01,88279.21) | 117075.47(47855.05,231290.60) | 2.23 | 216.44(61.09,521.05) | 304.21(124.17,594.82) | 0.76(0.10,1.43) |
| Denmark | both | 49568.81(20891.69,83062.26) | 10203.51(6370.96,14537.18) | -0.79 | 638.98(267.95,1066.61) | 94.62(59.04,134.20) | -6.84(-7.07,-6.60) |
| Djibouti | both | 512.04(162.91,1139.16) | 6250.77(3020.91,9866.57) | 11.21 | 312.75(104.89,699.29) | 935.11(463.33,1462.07) | 4.35(3.84,4.85) |
| Dominica | both | 303.14(110.30,596.63) | 363.00(156.90,627.36) | 0.20 | 438.87(158.65,858.46) | 410.39(178.14,709.88) | -0.51(-0.71,-0.31) |
| Dominican Republic | both | 10549.88(3205.26,23666.81) | 72386.27(33095.34,130415.43) | 5.86 | 255.94(77.86,580.50) | 744.83(339.35,1346.71) | 4.67(4.37,4.97) |
| Ecuador | both | 24138.03(11206.93,41443.72) | 56901.31(36356.31,80051.50) | 1.36 | 407.32(187.30,707.64) | 365.75(233.98,513.52) | -0.06(-0.39,0.26) |
| Egypt | both | 941528.05(744110.37,1121922.66) | 1998832.14(1456851.74,2606658.47) | 1.12 | 2951.16(2330.20,3504.22) | 2912.41(2141.67,3758.78) | 0.04(-0.09,0.16) |
| El Salvador | both | 9783.98(3668.21,18700.05) | 23292.57(14375.04,35822.53) | 1.38 | 306.41(114.80,588.67) | 392.50(241.70,603.95) | 0.90(0.64,1.15) |
| Equatorial Guinea | both | 472.01(117.34,1289.70) | 4378.55(2467.31,6906.56) | 8.28 | 222.09(57.23,603.01) | 860.97(486.67,1327.36) | 5.58(5.29,5.87) |
| Eritrea | both | 2785.67(772.62,6707.87) | 16144.11(6751.63,30542.03) | 4.80 | 233.54(65.55,559.17) | 520.31(219.58,965.08) | 2.49(2.22,2.76) |
| Estonia | both | 13236.77(5326.20,24300.55) | 2222.11(789.32,3911.29) | -0.83 | 656.70(263.24,1210.41) | 88.84(31.15,156.71) | -7.57(-8.31,-6.82) |
| Ethiopia | both | 24613.74(6814.54,65400.07) | 82289.34(39675.92,146062.70) | 2.34 | 110.25(31.32,289.85) | 187.08(90.01,333.09) | 1.90(1.58,2.21) |
| Micronesia (Federated States of) | both | 253.28(54.75,695.07) | 677.33(206.42,1555.67) | 1.67 | 464.56(101.07,1274.44) | 816.15(247.41,1883.71) | 1.93(1.74,2.11) |
| Fiji | both | 1818.96(429.93,4917.01) | 5431.71(1712.13,11720.62) | 1.99 | 406.73(97.34,1093.91) | 658.48(206.15,1421.02) | 1.47(0.93,2.02) |
| Finland | both | 22565.66(2691.17,48969.12) | 4826.04(973.91,9850.29) | -0.79 | 326.04(39.31,704.26) | 42.43(8.52,87.00) | -7.09(-7.60,-6.58) |
| France | both | 279167.44(127086.01,455051.90) | 112208.53(81765.12,143821.75) | -0.60 | 350.10(160.97,568.59) | 91.92(67.31,118.14) | -4.53(-4.74,-4.31) |
| Gabon | both | 3112.94(1317.14,5757.65) | 11082.18(6868.19,16257.40) | 2.56 | 537.32(228.34,989.42) | 1009.62(624.80,1468.37) | 2.07(1.64,2.50) |
| Georgia | both | 84133.80(43491.52,143173.91) | 52397.54(36058.54,70008.91) | -0.38 | 1385.16(714.51,2357.74) | 927.25(638.31,1238.54) | -1.82(-2.02,-1.62) |
| Germany | both | 923811.34(444155.71,1463865.00) | 273087.42(202296.59,346476.79) | -0.70 | 748.18(360.48,1183.98) | 153.41(114.46,194.66) | -5.46(-5.71,-5.21) |
| Ghana | both | 30227.05(13415.15,54041.05) | 168772.13(103246.78,241936.38) | 4.58 | 447.35(202.30,802.06) | 974.48(600.07,1389.97) | 3.22(3.02,3.42) |
| Greece | both | 101470.76(49009.85,163632.35) | 62327.39(47828.81,76726.10) | -0.39 | 687.19(331.04,1099.86) | 296.19(229.53,364.30) | -3.08(-3.41,-2.76) |
| Greenland | both | 121.80(26.75,318.01) | 83.07(7.55,237.85) | -0.32 | 312.22(69.05,828.73) | 117.44(10.76,334.67) | -4.04(-4.39,-3.68) |
| Grenada | both | 484.67(135.94,1055.54) | 714.08(285.76,1203.12) | 0.47 | 707.12(199.40,1534.25) | 629.70(249.14,1061.24) | -0.92(-1.16,-0.68) |
| Guam | both | 337.04(60.24,882.08) | 616.76(257.71,1055.36) | 0.83 | 390.87(70.20,1035.08) | 327.48(136.20,560.38) | -0.44(-1.15,0.27) |
| Guatemala | both | 10310.89(3439.07,21954.94) | 38170.76(21405.70,57567.33) | 2.70 | 257.71(88.27,544.80) | 319.53(179.85,478.89) | 0.11(-0.38,0.61) |
| Guinea | both | 5082.24(1488.49,12763.64) | 18497.20(7743.65,36937.45) | 2.64 | 150.86(44.87,372.74) | 317.75(134.74,629.09) | 3.13(2.98,3.28) |
| Guinea-Bissau | both | 1231.40(345.13,3093.72) | 4139.01(1762.65,7885.56) | 2.36 | 281.20(81.22,695.49) | 501.31(218.72,929.92) | 2.28(2.20,2.37) |
| Guyana | both | 6329.02(1700.44,12737.75) | 7501.70(3264.86,13540.74) | 0.19 | 1517.98(405.83,3064.89) | 1121.54(486.63,2026.01) | -0.90(-1.08,-0.72) |
| Haiti | both | 8717.61(2328.82,21446.03) | 28460.53(10154.39,59816.55) | 2.26 | 247.32(66.32,607.38) | 372.47(134.26,776.61) | 1.73(1.59,1.88) |
| Honduras | both | 4470.65(1427.90,9702.99) | 26513.33(14345.38,42568.27) | 4.93 | 195.23(63.14,428.18) | 426.04(232.15,678.28) | 3.06(2.71,3.41) |
| Hungary | both | 206684.47(114463.89,312397.68) | 94196.96(72934.05,119436.54) | -0.54 | 1462.04(806.24,2213.04) | 520.45(399.94,659.90) | -3.96(-4.13,-3.80) |
| Iceland | both | 436.89(38.97,1071.45) | 162.68(33.20,338.33) | -0.63 | 156.40(13.92,385.34) | 30.88(6.29,64.26) | -5.74(-5.94,-5.53) |
| India | both | 2887734.14(1388083.98,4882947.29) | 13456270.79(10296296.81,16523381.61) | 3.66 | 575.14(280.26,967.64) | 1113.77(857.72,1368.57) | 2.62(2.43,2.80) |
| Indonesia | both | 636598.72(273084.61,1123895.75) | 2151694.53(1556811.16,2816238.23) | 2.38 | 567.42(243.19,1016.30) | 923.16(669.04,1203.80) | 1.61(1.38,1.83) |
| Iran (Islamic Republic of) | both | 452698.88(368327.99,534712.14) | 744495.72(651758.40,844083.67) | 0.64 | 1647.77(1337.76,1946.09) | 982.23(857.62,1114.40) | -2.18(-2.45,-1.91) |
| Iraq | both | 187328.20(124594.99,249735.80) | 538854.54(397896.36,699619.57) | 1.88 | 2270.47(1516.13,3010.43) | 2198.11(1657.03,2787.07) | -0.18(-0.46,0.10) |
| Ireland | both | 20734.80(6281.92,38122.47) | 5370.35(2756.36,8149.03) | -0.74 | 518.66(158.10,950.39) | 74.33(38.10,112.03) | -7.06(-7.30,-6.83) |
| Israel | both | 32838.63(17973.34,49763.69) | 20046.05(16392.69,23725.39) | -0.39 | 684.96(374.73,1034.62) | 178.17(146.22,209.68) | -5.22(-5.45,-4.99) |
| Italy | both | 516755.61(287438.35,766594.28) | 216552.98(172943.44,261619.16) | -0.58 | 599.83(334.31,888.70) | 160.26(129.01,192.86) | -4.72(-4.82,-4.62) |
| Jamaica | both | 4782.42(2151.05,8867.69) | 10738.23(6691.82,15874.75) | 1.25 | 271.73(122.71,504.64) | 360.59(224.44,532.70) | 0.93(0.60,1.26) |
| Japan | both | 440141.23(147064.46,808113.03) | 388202.42(268670.70,532935.49) | -0.12 | 264.90(88.39,488.62) | 137.86(95.62,188.19) | -2.35(-2.64,-2.06) |
| Jordan | both | 21879.21(17491.81,26739.86) | 57017.25(45488.12,71551.39) | 1.61 | 1526.98(1225.22,1860.74) | 816.06(652.76,1015.76) | -2.67(-2.96,-2.38) |
| Kazakhstan | both | 147309.69(74002.32,251143.85) | 183226.32(120720.75,256167.75) | 0.24 | 1139.27(566.00,1944.94) | 1061.00(698.59,1474.70) | -1.03(-1.63,-0.42) |
| Kenya | both | 10882.13(4867.34,21190.48) | 69364.67(39977.79,110822.88) | 5.37 | 122.99(54.23,240.76) | 286.98(163.98,457.07) | 3.66(3.24,4.08) |
| Kiribati | both | 185.01(44.30,494.19) | 431.47(127.06,1056.96) | 1.33 | 402.40(97.11,1087.08) | 493.63(146.68,1226.55) | -0.20(-0.50,0.10) |
| Kuwait | both | 13103.63(11555.58,14714.01) | 35988.21(29405.12,43894.78) | 1.75 | 1599.26(1393.00,1795.77) | 1031.84(848.83,1254.03) | -1.09(-1.55,-0.62) |
| Kyrgyzstan | both | 25568.80(11100.49,46290.79) | 51034.59(31568.90,73679.37) | 1.00 | 832.10(360.94,1508.96) | 1092.19(673.55,1578.81) | 0.59(0.12,1.06) |
| Lao People's Democratic Republic | both | 5593.42(1581.60,13225.95) | 22530.41(11191.61,37174.88) | 3.03 | 251.62(71.54,596.05) | 472.37(235.22,771.30) | 2.17(1.89,2.45) |
| Latvia | both | 49650.02(25896.96,76273.47) | 17391.30(12058.69,24043.18) | -0.65 | 1409.42(734.17,2167.09) | 460.52(318.18,636.95) | -4.25(-4.86,-3.64) |
| Lebanon | both | 32085.07(22648.16,41989.50) | 57910.81(41100.85,73361.97) | 0.80 | 1362.89(968.59,1787.45) | 1101.02(784.12,1397.77) | -0.52(-0.77,-0.27) |
| Lesotho | both | 2479.31(1059.74,4757.22) | 9316.22(4895.36,15129.30) | 2.76 | 246.92(107.31,472.20) | 691.22(367.15,1104.29) | 4.37(4.01,4.72) |
| Liberia | both | 2341.69(793.29,5298.47) | 7442.65(3339.24,14119.36) | 2.18 | 208.05(70.76,469.84) | 328.23(150.24,615.92) | 2.66(2.16,3.15) |
| Libya | both | 21869.47(13977.87,31084.68) | 73457.55(51912.94,101426.31) | 2.36 | 1065.19(667.46,1507.41) | 1278.11(904.06,1765.04) | 0.35(0.11,0.59) |
| Lithuania | both | 45287.55(21260.37,73640.00) | 19568.93(12729.38,27743.96) | -0.57 | 1019.19(477.76,1652.08) | 362.81(236.54,517.15) | -3.40(-4.00,-2.80) |
| Luxembourg | both | 3027.89(1203.36,5144.22) | 909.48(601.53,1257.15) | -0.70 | 565.49(226.58,960.56) | 93.05(61.53,129.09) | -6.27(-6.52,-6.02) |
| North Macedonia | both | 35836.23(21778.05,49673.55) | 44991.44(34307.79,57347.82) | 0.26 | 1937.41(1168.47,2698.82) | 1467.56(1126.11,1865.62) | -1.30(-1.61,-0.98) |
| Madagascar | both | 7690.34(2657.14,17501.12) | 33853.28(14426.76,67154.57) | 3.40 | 133.47(46.93,302.95) | 263.75(113.43,516.70) | 2.68(2.52,2.85) |
| Malawi | both | 3787.97(1086.92,9976.11) | 13283.81(5541.54,26478.63) | 2.51 | 92.16(26.77,240.50) | 168.05(69.34,334.27) | 2.58(2.35,2.82) |
| Malaysia | both | 134053.76(63623.66,204117.25) | 190717.54(132609.20,261099.07) | 0.42 | 1352.37(631.05,2063.98) | 674.95(471.43,920.07) | -2.27(-2.38,-2.16) |
| Maldives | both | 392.50(122.57,881.67) | 925.53(601.48,1332.38) | 1.36 | 394.41(123.72,891.48) | 256.92(164.52,370.61) | -2.37(-2.71,-2.04) |
| Mali | both | 6075.35(1560.30,15997.84) | 20407.90(6843.52,43731.73) | 2.36 | 145.26(37.54,378.85) | 231.40(79.65,486.47) | 1.95(1.77,2.14) |
| Malta | both | 2678.44(1140.91,4502.61) | 1666.47(1222.75,2129.08) | -0.38 | 627.01(267.62,1052.89) | 193.79(143.37,246.30) | -3.98(-4.26,-3.70) |
| Marshall Islands | both | 66.61(15.03,177.11) | 283.66(96.74,585.87) | 3.26 | 342.28(77.88,908.20) | 654.11(223.70,1366.16) | 2.09(1.74,2.44) |
| Mauritania | both | 5122.30(2056.77,9779.08) | 14370.09(8012.47,20992.46) | 1.81 | 492.64(200.38,927.74) | 671.88(380.11,977.74) | 0.97(0.74,1.20) |
| Mauritius | both | 7966.97(4166.44,12278.98) | 7569.15(3728.00,11762.44) | -0.05 | 993.96(518.05,1537.17) | 440.11(217.79,680.89) | -3.80(-4.33,-3.28) |
| Mexico | both | 217278.12(113627.81,336623.92) | 428449.64(318021.73,544333.01) | 0.97 | 479.25(249.65,746.35) | 356.87(264.87,453.31) | -1.40(-1.68,-1.13) |
| Republic of Moldova | both | 47924.34(22542.92,79238.00) | 38122.18(22020.15,55779.96) | -0.20 | 1109.53(520.84,1842.15) | 668.75(384.21,980.77) | -1.63(-2.26,-1.00) |
| Mongolia | both | 11581.48(5102.66,20858.10) | 56738.73(37815.01,78524.75) | 3.90 | 1054.33(465.68,1893.55) | 2125.91(1444.19,2907.79) | 2.15(1.77,2.53) |
| Montenegro | both | 7878.61(4572.41,11548.94) | 8984.03(6856.60,11293.26) | 0.14 | 1265.66(732.47,1851.40) | 931.20(713.10,1166.65) | -1.07(-1.36,-0.79) |
| Morocco | both | 129640.60(68633.35,208947.50) | 541631.56(398697.72,701603.94) | 3.18 | 893.00(475.44,1437.88) | 1692.94(1258.40,2176.99) | 2.10(1.90,2.29) |
| Mozambique | both | 3917.92(947.27,11110.41) | 22065.94(9107.92,46233.48) | 4.63 | 62.64(15.51,176.08) | 184.30(76.97,381.37) | 4.97(4.53,5.40) |
| Myanmar | both | 119683.43(40659.16,264105.18) | 364221.87(219082.01,538260.83) | 2.04 | 471.07(158.67,1031.39) | 755.27(457.75,1106.68) | 1.97(1.68,2.27) |
| Namibia | both | 2990.14(1405.58,5286.28) | 9212.83(5412.07,13747.14) | 2.08 | 406.07(191.85,715.11) | 640.74(379.20,944.89) | 1.53(1.17,1.88) |
| Nepal | both | 25971.83(7006.55,61049.31) | 167097.70(96320.57,241433.47) | 5.43 | 252.66(70.74,580.72) | 726.50(421.18,1052.25) | 4.26(3.93,4.58) |
| Netherlands | both | 111938.63(53412.45,178709.58) | 37137.26(27511.42,47307.24) | -0.67 | 575.53(276.67,918.34) | 117.09(86.87,147.96) | -5.97(-6.23,-5.72) |
| New Zealand | both | 6796.62(471.24,17173.28) | 3184.85(582.55,6393.02) | -0.53 | 176.85(12.32,450.27) | 43.92(8.05,87.96) | -5.44(-5.72,-5.16) |
| Nicaragua | both | 2094.53(704.29,4494.03) | 13106.34(7177.06,21003.86) | 5.26 | 129.93(44.27,275.48) | 297.48(164.80,472.63) | 2.75(2.40,3.11) |
| Niger | both | 4731.91(1012.59,13882.76) | 17325.66(4491.29,43891.53) | 2.66 | 159.77(35.47,471.87) | 213.70(56.45,530.09) | 1.06(0.88,1.24) |
| Nigeria | both | 135701.62(56953.19,285370.37) | 527348.87(319980.76,778172.89) | 2.89 | 306.29(132.48,631.38) | 589.93(362.47,857.85) | 2.31(2.06,2.56) |
| Democratic People's Republic of Korea | both | 90451.20(34954.52,167955.88) | 340896.99(210819.44,492073.18) | 2.77 | 535.77(211.42,999.49) | 1047.98(652.66,1519.17) | 2.59(2.47,2.72) |
| Northern Mariana Islands | both | 141.23(43.96,342.77) | 199.43(103.34,322.06) | 0.41 | 517.58(163.65,1240.44) | 349.27(181.44,563.86) | -1.24(-1.81,-0.67) |
| Norway | both | 24110.88(6344.78,46228.10) | 3757.38(1406.66,6596.57) | -0.84 | 376.71(99.54,714.46) | 41.25(15.59,71.91) | -7.81(-8.08,-7.54) |
| Oman | both | 14087.51(7618.22,22214.85) | 34130.98(26111.34,42981.15) | 1.42 | 1881.23(1034.05,2971.14) | 1830.01(1419.18,2292.82) | -0.47(-1.08,0.14) |
| Pakistan | both | 225915.92(76030.53,474086.97) | 1644671.01(1103957.38,2269923.86) | 6.28 | 374.82(127.55,783.82) | 1286.46(858.35,1766.62) | 4.62(4.30,4.95) |
| Palestine | both | 9040.27(4468.13,14845.85) | 31258.39(23225.68,38996.19) | 2.46 | 1023.96(504.08,1665.54) | 1283.89(957.24,1602.13) | 0.16(-0.30,0.62) |
| Panama | both | 4787.70(2209.80,8722.84) | 7915.28(4643.21,11736.48) | 0.65 | 309.61(142.55,565.63) | 190.22(111.45,282.15) | -1.16(-1.40,-0.91) |
| Papua New Guinea | both | 3512.22(642.54,11192.66) | 16182.58(3664.80,41689.04) | 3.61 | 161.96(29.40,505.75) | 277.31(63.57,703.36) | 1.93(1.75,2.10) |
| Paraguay | both | 5849.37(2484.00,10891.13) | 15630.99(9607.95,23811.89) | 1.67 | 250.49(107.18,463.79) | 270.96(166.45,415.01) | 0.09(-0.16,0.33) |
| Peru | both | 58707.58(27880.05,97651.93) | 94244.35(63443.65,133550.20) | 0.61 | 454.48(214.83,757.27) | 286.14(192.71,405.57) | -1.57(-2.05,-1.08) |
| Philippines | both | 99919.52(50405.93,169501.35) | 612137.67(425087.04,832521.83) | 5.13 | 320.19(160.44,542.29) | 699.34(490.74,946.56) | 3.09(2.16,4.02) |
| Poland | both | 682876.24(391643.20,979719.09) | 363971.34(291029.41,447936.34) | -0.47 | 1583.61(910.63,2267.38) | 545.93(436.69,677.51) | -4.11(-4.29,-3.93) |
| Portugal | both | 58759.47(18950.99,110580.11) | 18554.87(10802.94,26759.44) | -0.68 | 433.88(141.66,813.38) | 83.77(48.77,121.10) | -6.21(-6.58,-5.83) |
| Puerto Rico | both | 6002.06(42.02,19850.98) | 3750.28(795.81,6952.95) | -0.38 | 166.62(1.17,552.22) | 60.46(12.63,112.85) | -4.28(-4.87,-3.69) |
| Qatar | both | 4001.06(3241.08,4938.18) | 13375.71(10144.92,17398.36) | 2.34 | 2808.22(2272.47,3384.87) | 1307.69(1023.70,1650.29) | -2.88(-3.16,-2.60) |
| Romania | both | 311873.63(163245.50,487324.66) | 224434.74(173429.64,284102.94) | -0.28 | 1146.24(600.60,1797.44) | 636.23(492.51,809.66) | -2.80(-3.21,-2.38) |
| Russian Federation | both | 2315290.38(1012874.49,3796999.53) | 1362022.37(774149.05,1954907.29) | -0.41 | 1305.26(567.68,2143.35) | 602.09(344.96,868.65) | -3.03(-3.76,-2.29) |
| Rwanda | both | 8372.76(2448.92,19164.80) | 18523.95(7640.70,34708.32) | 1.21 | 267.20(80.27,605.99) | 293.28(122.00,550.86) | -0.33(-0.77,0.12) |
| Saint Lucia | both | 535.40(191.75,1091.88) | 984.09(436.54,1652.19) | 0.84 | 611.67(217.13,1245.91) | 455.69(201.51,764.55) | -1.66(-2.01,-1.30) |
| Saint Vincent and the Grenadines | both | 416.53(125.07,893.62) | 834.19(333.29,1434.69) | 1.00 | 576.69(172.09,1237.79) | 622.30(247.83,1072.78) | -0.23(-0.50,0.05) |
| Samoa | both | 410.78(98.69,1064.23) | 774.34(217.74,1759.01) | 0.89 | 441.30(105.96,1134.35) | 493.10(139.27,1111.99) | 0.13(0.02,0.24) |
| Sao Tome and Principe | both | 121.73(46.71,254.82) | 689.36(351.90,1142.71) | 4.66 | 189.01(72.81,392.86) | 609.49(315.10,1007.22) | 4.53(4.26,4.80) |
| Saudi Arabia | both | 85241.14(46245.69,135089.09) | 495666.04(381973.49,630972.10) | 4.81 | 1289.16(716.26,2013.36) | 2068.77(1644.22,2546.02) | 1.29(0.66,1.93) |
| Senegal | both | 9178.79(3115.02,20889.41) | 34187.16(16091.59,60517.19) | 2.72 | 273.27(92.68,612.09) | 437.05(209.33,765.37) | 1.46(1.26,1.66) |
| Serbia | both | 167887.10(93033.22,242805.25) | 148654.25(115399.02,187789.06) | -0.11 | 1534.34(864.11,2224.41) | 969.72(750.65,1219.36) | -1.98(-2.39,-1.57) |
| Seychelles | both | 394.42(216.68,617.64) | 582.24(313.19,875.24) | 0.48 | 699.08(385.01,1095.56) | 501.77(270.09,762.26) | -1.64(-1.83,-1.45) |
| Sierra Leone | both | 4022.58(1267.25,9890.53) | 13457.39(5380.64,27226.28) | 2.35 | 204.98(64.64,501.85) | 344.90(140.43,691.56) | 2.35(2.05,2.65) |
| Singapore | both | 18587.00(6513.75,32231.92) | 17355.03(11336.05,23382.71) | -0.07 | 795.77(276.32,1391.60) | 217.76(142.31,292.56) | -4.69(-5.05,-4.33) |
| Slovakia | both | 103176.37(56684.58,151231.82) | 53460.96(39410.08,69057.83) | -0.48 | 1744.68(955.69,2557.55) | 595.76(439.38,768.18) | -3.86(-4.07,-3.66) |
| Slovenia | both | 20630.11(11074.46,32714.56) | 9113.45(6746.35,11999.33) | -0.56 | 852.36(455.93,1351.37) | 221.03(163.09,289.93) | -5.30(-5.51,-5.09) |
| Solomon Islands | both | 421.88(74.15,1353.60) | 2367.06(637.58,6174.67) | 4.61 | 255.11(45.59,817.14) | 603.21(158.96,1586.28) | 2.85(2.71,2.99) |
| Somalia | both | 2005.06(389.60,6268.96) | 6119.60(1318.81,18799.76) | 2.05 | 69.16(13.86,212.43) | 81.37(18.10,251.00) | 1.01(0.74,1.29) |
| South Africa | both | 138823.11(105631.55,175021.22) | 276273.06(221855.55,334887.50) | 0.99 | 591.48(447.64,747.31) | 592.54(473.74,720.57) | 0.09(-0.35,0.54) |
| Republic of Korea | both | 393879.26(219570.51,579321.66) | 238620.70(194468.95,294485.24) | -0.39 | 1255.51(695.75,1869.61) | 276.38(225.66,341.01) | -5.58(-5.95,-5.22) |
| South Sudan | both | 4390.46(1438.86,9984.96) | 9626.43(3802.69,17901.52) | 1.19 | 175.87(57.80,398.65) | 238.62(96.27,441.33) | 1.37(1.21,1.54) |
| Spain | both | 175467.92(68212.89,305422.84) | 74614.35(50528.76,100362.07) | -0.57 | 337.18(132.22,584.64) | 84.34(57.24,113.58) | -4.85(-5.14,-4.56) |
| Sri Lanka | both | 52278.10(22217.79,94011.48) | 108010.19(67664.70,159670.04) | 1.07 | 459.75(195.25,823.22) | 428.48(271.44,628.67) | -0.08(-0.55,0.39) |
| Sudan | both | 52594.95(17746.51,114489.74) | 323470.74(189490.67,499947.93) | 5.15 | 527.69(181.99,1142.09) | 1572.29(941.97,2398.56) | 4.08(3.96,4.19) |
| Suriname | both | 2344.91(830.51,4338.46) | 4476.89(2171.19,7392.58) | 0.91 | 862.05(304.61,1608.41) | 723.40(351.79,1192.69) | -0.85(-1.23,-0.48) |
| Eswatini | both | 982.08(449.07,1677.22) | 3991.33(2219.29,6138.23) | 3.06 | 317.71(145.58,539.33) | 643.22(356.94,975.84) | 2.70(2.23,3.17) |
| Sweden | both | 38698.93(6735.15,80767.25) | 6812.34(1791.54,13574.27) | -0.82 | 267.33(46.99,556.77) | 34.58(8.97,68.83) | -7.03(-7.39,-6.67) |
| Switzerland | both | 43820.94(19261.22,72389.47) | 12639.52(8425.64,17093.48) | -0.71 | 429.99(190.21,705.50) | 74.84(50.12,100.12) | -6.07(-6.25,-5.89) |
| Syrian Arab Republic | both | 135480.07(95905.46,179202.84) | 235628.86(167739.77,321179.63) | 0.74 | 2280.97(1605.01,3027.93) | 1861.30(1351.83,2527.08) | -1.08(-1.28,-0.88) |
| Taiwan (Province of China) | both | 104839.52(57308.27,164666.27) | 121329.75(94985.41,155804.71) | 0.16 | 642.52(351.84,1013.08) | 322.42(251.81,413.44) | -2.43(-2.59,-2.27) |
| Tajikistan | both | 19946.25(6846.54,39952.20) | 90070.46(48285.17,139739.20) | 3.52 | 699.98(239.89,1408.99) | 1853.67(981.05,2899.28) | 3.45(3.22,3.67) |
| United Republic of Tanzania | both | 11597.89(4043.94,25897.29) | 58344.14(26927.01,106223.02) | 4.03 | 100.93(35.72,223.23) | 227.04(105.85,408.23) | 3.14(2.99,3.29) |
| Thailand | both | 228853.90(111140.64,371188.96) | 476156.53(344681.90,647394.68) | 1.08 | 591.81(286.22,956.50) | 480.85(348.25,652.89) | -1.31(-1.66,-0.96) |
| Bahamas | both | 1059.78(284.40,2033.54) | 1727.50(507.57,3264.10) | 0.63 | 630.23(165.13,1211.65) | 417.76(121.63,794.98) | -1.66(-1.86,-1.47) |
| Gambia | both | 778.38(231.12,1883.74) | 5225.75(2513.63,9044.39) | 5.71 | 207.14(63.35,490.51) | 515.29(254.08,884.92) | 3.25(3.07,3.44) |
| Timor-Leste | both | 310.73(73.91,864.63) | 3174.30(1332.43,5763.73) | 9.22 | 98.42(24.06,270.13) | 382.14(163.17,693.64) | 6.06(5.40,6.73) |
| Togo | both | 3589.13(1296.12,7508.28) | 19497.33(9279.89,33697.50) | 4.43 | 265.20(97.74,542.25) | 478.87(231.55,814.01) | 2.05(1.79,2.30) |
| Tonga | both | 131.16(35.09,333.24) | 271.43(85.51,582.89) | 1.07 | 224.61(60.50,570.80) | 334.84(105.62,718.48) | 1.17(0.92,1.43) |
| Trinidad and Tobago | both | 9931.29(2117.05,19404.74) | 11679.52(3955.62,21074.82) | 0.18 | 1145.74(241.55,2258.59) | 633.42(215.71,1145.62) | -2.77(-3.13,-2.41) |
| Tunisia | both | 55965.81(36334.54,75643.44) | 130418.14(88769.85,183356.14) | 1.33 | 1088.37(708.03,1467.34) | 1033.15(704.73,1446.09) | -0.43(-0.77,-0.09) |
| Turkey | both | 411000.24(308843.97,501619.64) | 566663.32(437266.59,727157.04) | 0.38 | 1069.00(805.50,1303.88) | 636.76(491.68,816.36) | -1.98(-2.18,-1.78) |
| Turkmenistan | both | 37622.93(15777.22,62234.98) | 82356.39(50008.14,121190.74) | 1.19 | 1895.84(798.99,3172.84) | 1994.50(1208.25,2932.28) | -0.51(-0.91,-0.11) |
| Uganda | both | 6670.88(1844.89,17032.62) | 40611.89(19291.35,72570.61) | 5.09 | 99.37(28.25,246.36) | 266.12(127.89,472.52) | 3.81(3.54,4.08) |
| Ukraine | both | 958392.97(461684.92,1511739.79) | 821428.91(509406.60,1197583.94) | -0.14 | 1364.38(659.66,2147.79) | 1129.26(700.83,1643.67) | -1.14(-1.60,-0.68) |
| United Arab Emirates | both | 15006.11(11194.60,20486.86) | 97950.51(67401.54,142143.27) | 5.53 | 2472.36(1902.36,3294.58) | 1477.12(1049.98,2042.20) | -2.01(-2.34,-1.68) |
| United Kingdom | both | 554311.16(232504.18,928442.97) | 132693.74(88498.23,179803.72) | -0.76 | 643.63(274.12,1063.53) | 116.53(77.85,156.44) | -6.38(-6.59,-6.17) |
| United States of America | both | 1338815.17(518756.57,2371200.97) | 561964.67(301782.83,846751.30) | -0.58 | 436.32(169.94,769.73) | 110.28(59.58,165.97) | -5.13(-5.40,-4.87) |
| Uruguay | both | 13474.72(4382.52,27750.18) | 7787.12(3901.44,12191.33) | -0.42 | 360.70(117.34,737.05) | 155.50(77.84,242.74) | -3.18(-3.40,-2.96) |
| Uzbekistan | both | 150277.36(61738.53,256219.09) | 599753.77(382297.56,837648.47) | 2.99 | 1301.08(533.62,2225.00) | 2996.10(1908.16,4152.13) | 2.59(1.98,3.21) |
| Vanuatu | both | 238.11(51.03,632.97) | 1141.63(325.48,2639.45) | 3.79 | 309.34(67.25,822.78) | 580.98(164.88,1343.47) | 1.75(1.52,1.97) |
| Venezuela (Bolivarian Republic of) | both | 84748.81(38412.68,138050.98) | 195687.46(128670.00,286362.13) | 1.31 | 793.13(359.83,1302.36) | 656.54(430.09,960.53) | -0.86(-1.02,-0.71) |
| Viet nam | both | 135269.89(51059.04,276241.55) | 657450.72(443777.78,888113.15) | 3.86 | 330.75(125.98,677.45) | 683.92(465.26,913.08) | 3.11(2.58,3.64) |
| Virginia | both | 32911.95(14151.21,56515.06) | 10722.61(3927.28,18392.41) | -0.67 | 470.61(202.95,811.34) | 81.25(29.73,139.31) | -6.31(-6.71,-5.91) |
| Yemen | both | 26385.26(7168.57,66955.70) | 218332.80(106542.19,350960.81) | 7.27 | 487.23(135.83,1234.78) | 1462.87(715.24,2337.94) | 4.24(3.99,4.49) |
| Zambia | both | 5686.82(2110.20,11832.09) | 36598.26(18381.36,60222.31) | 5.44 | 184.64(68.96,392.07) | 491.94(249.83,805.95) | 3.55(3.37,3.74) |
| Zimbabwe | both | 9142.01(4146.47,16343.50) | 25737.73(12173.38,44820.28) | 1.82 | 221.77(101.22,391.91) | 351.56(166.13,608.12) | 1.72(1.33,2.11) |
| Monaco | both | 154.74(15.55,337.61) | 122.99(71.23,178.82) | -0.21 | 234.97(23.53,505.86) | 143.55(82.68,209.50) | -1.33(-1.97,-0.68) |
| San Marino | both | 59.83(13.63,122.05) | 53.02(21.04,95.59) | -0.11 | 185.13(42.68,375.31) | 87.25(34.69,157.50) | -2.92(-3.36,-2.48) |
| Saint Kitts and Nevis | both | 206.40(71.07,406.57) | 173.04(76.33,291.65) | -0.16 | 581.55(198.72,1146.88) | 254.15(113.31,423.51) | -3.57(-4.06,-3.08) |
| Cook Islands | both | 33.47(12.35,78.18) | 35.69(7.05,83.97) | 0.07 | 242.83(90.23,568.34) | 154.12(30.97,361.75) | -1.65(-2.00,-1.29) |
| Nauru | both | 31.06(8.85,88.60) | 31.99(8.63,74.80) | 0.03 | 563.30(155.99,1642.14) | 495.83(133.59,1140.89) | -1.07(-1.36,-0.78) |
| Niue | both | 7.59(2.94,16.55) | 6.71(1.38,15.37) | -0.12 | 364.48(141.84,793.91) | 319.51(65.55,737.51) | -1.05(-1.31,-0.80) |
| Palau | both | 44.25(0.43,135.69) | 80.55(0.30,202.23) | 0.82 | 390.64(3.78,1209.27) | 339.73(1.29,853.19) | 0.39(-0.44,1.23) |
| Tokelau | both | 4.74(0.13,17.39) | 3.36(0.02,9.18) | -0.29 | 365.49(9.84,1347.01) | 253.02(1.26,691.64) | -1.71(-2.02,-1.41) |
| Tuvalu | both | 18.10(4.50,52.18) | 40.82(15.28,87.98) | 1.26 | 243.19(60.45,699.67) | 380.24(142.17,816.58) | 1.23(0.99,1.47) |
| Afghanistan | female | 14229.12(3354.63,38347.12) | 67274.53(28594.39,133512.19) | 3.73 | 392.38(94.82,1051.83) | 860.33(368.38,1697.03) | 3.00(2.42,3.59) |
| Albania | female | 4165.87(1928.50,7057.05) | 9463.27(6663.11,12960.60) | 1.27 | 386.12(178.90,657.62) | 424.57(298.68,582.18) | 0.99(0.58,1.39) |
| Algeria | female | 91926.68(56641.05,131113.40) | 186205.39(129305.78,249447.56) | 1.03 | 1530.85(949.85,2167.31) | 1158.01(804.56,1546.68) | -1.33(-1.55,-1.11) |
| American Samoa | female | 22.44(6.81,57.73) | 50.28(22.09,96.53) | 1.24 | 184.78(55.84,476.97) | 199.04(87.12,384.64) | -0.02(-0.27,0.24) |
| Andorra | female | 38.78(12.55,73.70) | 37.52(19.60,59.69) | -0.03 | 149.51(47.95,284.47) | 53.16(27.68,84.77) | -3.44(-3.61,-3.28) |
| Angola | female | 2248.72(633.21,5783.70) | 24761.73(11837.29,43553.22) | 10.01 | 109.52(31.32,275.65) | 392.88(183.03,686.37) | 4.42(4.18,4.66) |
| Antigua and Barbuda | female | 137.46(38.36,274.09) | 182.77(68.74,312.27) | 0.33 | 461.24(127.47,918.15) | 341.07(128.58,583.59) | -1.57(-1.91,-1.22) |
| Argentina | female | 69738.98(27478.40,126127.33) | 53263.04(33708.57,75084.17) | -0.24 | 397.84(157.18,718.34) | 180.81(114.29,254.21) | -2.87(-3.19,-2.55) |
| Armenia | female | 14153.72(6590.03,23417.12) | 17879.97(12360.44,23624.11) | 0.26 | 959.98(444.05,1598.54) | 747.40(518.53,984.50) | -1.05(-1.26,-0.84) |
| Australia | female | 13375.63(1767.65,31230.73) | 6556.27(1705.35,12082.98) | -0.51 | 122.66(16.11,286.57) | 29.98(7.73,54.99) | -5.30(-5.65,-4.95) |
| Austria | female | 34258.66(17134.45,54114.55) | 10065.69(7387.52,12973.79) | -0.71 | 454.97(229.22,711.62) | 97.47(72.31,123.96) | -5.74(-5.93,-5.54) |
| Azerbaijan | female | 22996.11(10431.30,41124.19) | 62626.62(37336.21,92908.87) | 1.72 | 785.20(354.55,1407.82) | 1344.95(803.29,1997.90) | 1.75(1.44,2.06) |
| Bahrain | female | 1978.11(1576.91,2395.58) | 3334.79(2689.50,4162.13) | 0.69 | 2439.40(1955.56,2927.18) | 935.23(757.95,1156.39) | -3.73(-4.01,-3.44) |
| Bangladesh | female | 54345.80(17027.13,121144.61) | 404752.51(246819.82,590325.35) | 6.45 | 237.78(75.59,523.27) | 629.67(386.39,914.43) | 3.93(3.75,4.10) |
| Barbados | female | 899.26(267.98,1670.37) | 892.26(394.30,1475.78) | -0.01 | 538.36(159.52,983.28) | 340.73(151.13,562.80) | -1.69(-2.04,-1.33) |
| Belarus | female | 88094.58(46098.04,135173.59) | 64950.24(45753.55,88433.56) | -0.26 | 1063.87(555.49,1627.58) | 641.91(450.57,875.45) | -1.95(-2.52,-1.37) |
| Belgium | female | 37113.68(17821.03,59532.27) | 12285.07(9182.34,15575.28) | -0.67 | 414.64(201.20,660.16) | 99.30(75.02,125.44) | -4.98(-5.18,-4.79) |
| Belize | female | 128.67(28.39,314.82) | 447.34(167.61,777.26) | 2.48 | 274.58(60.82,673.41) | 308.27(113.42,540.73) | -0.46(-1.01,0.08) |
| Benin | female | 1389.63(463.31,3312.74) | 6906.68(3135.66,12766.72) | 3.97 | 133.88(44.61,317.72) | 261.25(118.32,478.25) | 2.31(1.97,2.64) |
| Bermuda | female | 85.74(21.80,216.38) | 29.27(6.13,56.03) | -0.66 | 242.75(61.65,613.45) | 41.75(8.83,80.73) | -7.04(-7.60,-6.48) |
| Bhutan | female | 163.78(44.33,406.61) | 1138.89(651.52,1771.39) | 5.95 | 122.39(33.07,304.40) | 404.35(232.05,620.15) | 5.02(4.72,5.32) |
| Bolivia (Plurinational State of) | female | 10059.39(3538.39,19863.38) | 20023.29(11339.78,31420.10) | 0.99 | 547.19(191.49,1083.14) | 424.92(242.59,664.02) | -1.20(-1.69,-0.71) |
| Bosnia and Herzegovina | female | 14560.93(7198.00,23366.53) | 22912.31(16985.28,29072.43) | 0.57 | 666.37(330.22,1072.31) | 693.58(514.45,878.82) | 0.12(-0.27,0.51) |
| Botswana | female | 829.56(391.92,1497.65) | 4822.39(2777.36,7576.90) | 4.81 | 259.12(124.16,469.05) | 608.91(357.19,947.31) | 3.32(2.88,3.75) |
| Brazil | female | 194519.85(87588.65,351042.23) | 247806.30(171560.16,331929.22) | 0.27 | 382.43(170.98,698.22) | 190.21(131.76,254.78) | -2.57(-2.82,-2.32) |
| Brunei Darussalam | female | 170.02(68.93,331.55) | 203.91(60.97,362.21) | 0.20 | 307.47(123.32,601.86) | 126.67(36.99,222.53) | -2.99(-3.64,-2.34) |
| Bulgaria | female | 86234.36(46773.33,128760.28) | 61801.36(47506.80,77877.31) | -0.28 | 1326.63(724.25,1972.80) | 752.34(571.26,955.12) | -2.85(-3.26,-2.44) |
| Burkina Faso | female | 1736.64(446.25,4531.79) | 8173.16(2817.46,17603.41) | 3.71 | 73.60(19.29,187.96) | 158.05(55.78,332.43) | 3.29(3.05,3.54) |
| Burundi | female | 1790.30(454.17,4809.05) | 3060.16(977.21,7136.80) | 0.71 | 133.12(33.86,354.01) | 133.62(43.21,310.01) | -0.35(-0.65,-0.05) |
| Cambodia | female | 4249.33(1192.91,10398.78) | 19081.81(8954.33,33318.56) | 3.49 | 157.12(44.58,385.53) | 275.67(129.72,478.54) | 2.13(1.92,2.34) |
| Cameroon | female | 6875.91(2945.36,13082.42) | 38719.65(21133.69,59407.08) | 4.63 | 292.22(126.69,552.84) | 594.11(329.40,890.79) | 2.60(2.41,2.78) |
| Canada | female | 30953.57(8031.11,61838.76) | 14180.44(6682.55,22843.10) | -0.54 | 170.28(44.21,339.88) | 40.01(18.94,64.34) | -5.55(-5.83,-5.27) |
| Cabo Verde | female | 209.14(88.05,405.08) | 1533.47(1011.27,2081.54) | 6.33 | 159.86(66.05,308.43) | 633.66(418.65,857.63) | 4.70(4.24,5.16) |
| Central African Republic | female | 1048.42(243.49,2663.00) | 3004.25(903.80,7348.07) | 1.87 | 161.00(37.66,411.36) | 244.07(75.33,588.27) | 1.51(1.28,1.74) |
| Chad | female | 1437.01(351.32,3972.08) | 5412.55(1895.41,11874.53) | 2.77 | 97.64(24.60,266.38) | 198.49(70.26,435.99) | 2.85(2.66,3.05) |
| Chile | female | 21819.86(10644.09,36274.29) | 28386.27(22778.86,34232.96) | 0.30 | 400.58(195.36,667.83) | 219.38(175.61,264.35) | -1.98(-2.12,-1.83) |
| China | female | 2177701.08(1029735.76,3873673.43) | 7723750.10(5982203.07,9749080.43) | 2.55 | 505.82(240.02,900.76) | 754.55(583.84,948.90) | 1.64(1.27,2.00) |
| Colombia | female | 43666.22(19850.79,77690.18) | 73828.46(50429.10,101362.13) | 0.69 | 454.83(207.24,813.74) | 257.40(175.45,353.15) | -2.32(-2.51,-2.14) |
| Comoros | female | 98.51(32.36,237.21) | 473.98(224.69,854.67) | 3.81 | 83.95(27.85,199.46) | 176.87(83.27,319.93) | 2.38(2.21,2.54) |
| Congo | female | 2238.49(785.64,4828.12) | 12066.33(6428.62,19767.11) | 4.39 | 365.08(130.03,793.11) | 848.24(449.35,1382.04) | 2.96(2.73,3.19) |
| Costa Rica | female | 2113.55(940.67,3790.57) | 4904.70(3442.16,6573.73) | 1.32 | 226.64(100.53,407.16) | 176.77(124.21,236.72) | -1.16(-1.42,-0.89) |
| Côte d'Ivoire | female | 3894.02(1324.11,8925.69) | 19825.24(9075.29,34986.45) | 4.09 | 197.55(68.92,439.96) | 366.71(171.14,639.41) | 2.44(2.13,2.75) |
| Croatia | female | 35202.30(18971.71,53441.58) | 17628.78(13298.41,22440.14) | -0.50 | 942.21(508.31,1427.67) | 333.27(252.68,423.98) | -3.85(-4.08,-3.61) |
| Cuba | female | 31939.74(12334.96,57410.95) | 30440.22(15279.18,49884.44) | -0.05 | 610.53(235.05,1094.13) | 308.07(155.67,503.29) | -2.54(-2.78,-2.29) |
| Cyprus | female | 2081.73(862.05,3503.03) | 1635.60(1238.74,2120.95) | -0.21 | 538.52(220.47,917.46) | 164.76(125.28,212.46) | -4.69(-4.93,-4.45) |
| Czechia | female | 92960.63(49325.90,138342.16) | 32400.27(24853.49,41082.30) | -0.65 | 1120.26(604.24,1666.72) | 266.41(204.20,336.54) | -5.18(-5.30,-5.05) |
| Democratic Republic of the Congo | female | 13654.85(3744.49,34544.20) | 45862.56(17807.65,92741.64) | 2.36 | 158.81(45.10,395.82) | 232.11(91.61,460.46) | 0.91(0.28,1.54) |
| Denmark | female | 19583.70(8252.29,33155.84) | 3988.40(2470.95,5700.32) | -0.80 | 425.00(179.49,719.48) | 66.09(41.36,93.51) | -6.71(-6.97,-6.45) |
| Djibouti | female | 159.65(50.39,370.89) | 2103.01(977.10,3509.28) | 12.17 | 217.08(69.78,491.68) | 727.40(343.19,1178.90) | 4.85(4.33,5.37) |
| Dominica | female | 143.18(49.46,290.47) | 156.04(66.80,270.71) | 0.09 | 350.47(121.43,709.15) | 342.17(147.95,597.02) | -0.33(-0.49,-0.16) |
| Dominican Republic | female | 3837.37(1127.23,9152.70) | 26923.32(12247.90,49194.80) | 6.02 | 185.28(53.72,442.58) | 543.91(247.71,996.81) | 4.73(4.39,5.06) |
| Ecuador | female | 9636.40(4234.35,17357.69) | 22772.53(14552.97,32254.20) | 1.36 | 325.09(143.06,589.33) | 284.80(181.83,402.88) | -0.15(-0.50,0.21) |
| Egypt | female | 396338.07(310475.53,476834.94) | 819309.30(587394.94,1069590.51) | 1.07 | 2572.45(2011.82,3104.82) | 2794.24(2036.05,3588.44) | 0.46(0.31,0.61) |
| El Salvador | female | 3668.85(1386.35,7266.80) | 10271.74(6282.47,15780.20) | 1.80 | 219.58(83.35,436.45) | 299.84(183.34,461.56) | 1.23(0.96,1.50) |
| Equatorial Guinea | female | 156.82(36.60,443.87) | 2203.62(1192.33,3566.01) | 13.05 | 138.05(32.15,384.99) | 790.65(437.58,1241.06) | 7.35(6.87,7.83) |
| Eritrea | female | 876.93(225.15,2317.43) | 5769.17(2388.42,11242.12) | 5.58 | 140.64(36.98,374.01) | 365.18(153.45,703.15) | 3.23(3.00,3.45) |
| Estonia | female | 5933.07(2475.67,11024.60) | 1020.44(398.06,1764.15) | -0.83 | 444.12(185.37,827.00) | 59.21(23.28,101.18) | -7.78(-8.42,-7.12) |
| Ethiopia | female | 7723.43(2096.82,20854.10) | 29690.22(14511.49,52715.29) | 2.84 | 72.67(19.61,192.18) | 143.40(71.02,254.76) | 2.17(1.86,2.48) |
| Micronesia (Federated States of) | female | 63.84(13.43,181.99) | 208.67(59.14,488.69) | 2.27 | 251.82(53.36,718.54) | 526.60(151.92,1238.11) | 2.55(2.35,2.75) |
| Fiji | female | 473.39(107.62,1351.89) | 1770.24(556.84,3898.64) | 2.74 | 227.42(51.65,651.50) | 434.06(136.04,956.50) | 2.06(1.48,2.65) |
| Finland | female | 8738.80(1089.99,19309.05) | 1819.89(377.92,3720.41) | -0.79 | 197.97(24.91,430.17) | 26.27(5.36,53.50) | -7.10(-7.59,-6.61) |
| France | female | 113105.51(50721.83,185412.83) | 44345.77(32183.38,57913.94) | -0.61 | 227.18(102.57,368.89) | 59.30(43.27,76.78) | -4.50(-4.69,-4.31) |
| Gabon | female | 1061.67(440.87,2024.76) | 4188.39(2502.20,6303.70) | 2.95 | 344.24(142.65,654.38) | 751.30(448.46,1114.59) | 2.59(1.99,3.19) |
| Georgia | female | 34772.01(16623.60,60366.15) | 19621.37(13317.76,26662.51) | -0.44 | 948.85(454.76,1645.37) | 542.11(369.01,735.69) | -2.42(-2.64,-2.20) |
| Germany | female | 411048.43(196486.45,656775.79) | 109377.78(80391.29,139916.81) | -0.73 | 513.39(245.29,818.41) | 103.02(76.41,131.24) | -5.56(-5.82,-5.30) |
| Ghana | female | 15258.59(6659.75,28407.09) | 84935.01(49017.66,126556.51) | 4.57 | 439.04(194.29,806.25) | 905.74(525.48,1338.68) | 3.04(2.79,3.30) |
| Greece | female | 43116.06(20837.37,69879.75) | 25630.37(19576.35,31880.20) | -0.41 | 519.16(250.90,842.87) | 193.64(149.48,238.62) | -3.73(-4.06,-3.39) |
| Greenland | female | 45.67(11.36,119.56) | 25.14(2.86,68.62) | -0.45 | 255.57(63.36,671.72) | 79.76(9.12,218.76) | -5.11(-5.60,-4.61) |
| Grenada | female | 199.28(52.92,448.04) | 292.28(114.79,497.65) | 0.47 | 499.73(133.76,1109.40) | 503.71(197.33,858.49) | -0.63(-1.05,-0.22) |
| Guam | female | 113.24(23.10,293.86) | 225.08(97.46,379.98) | 0.99 | 289.74(59.08,762.94) | 237.93(102.73,401.22) | -0.70(-1.42,0.03) |
| Guatemala | female | 4222.17(1420.21,9418.45) | 16199.73(8674.19,25023.75) | 2.84 | 210.62(71.65,464.65) | 251.19(134.27,388.11) | -0.06(-0.62,0.51) |
| Guinea | female | 2238.99(645.23,5720.36) | 7251.30(2924.85,15311.06) | 2.24 | 132.75(38.93,334.26) | 252.44(103.81,529.93) | 2.72(2.54,2.91) |
| Guinea-Bissau | female | 406.90(114.33,1074.88) | 1583.05(660.20,3204.74) | 2.89 | 179.35(51.62,463.61) | 363.55(155.09,728.88) | 2.75(2.67,2.84) |
| Guyana | female | 2277.32(547.15,4776.18) | 3033.65(1298.39,5491.63) | 0.33 | 1092.61(261.96,2301.69) | 882.51(376.58,1601.43) | -0.63(-0.82,-0.45) |
| Haiti | female | 3629.20(960.15,9330.76) | 13192.96(4669.34,28047.31) | 2.64 | 198.91(52.37,516.11) | 326.04(115.40,684.98) | 2.04(1.90,2.18) |
| Honduras | female | 1736.46(565.38,3836.67) | 11244.97(5657.40,19086.68) | 5.48 | 144.80(48.36,316.31) | 337.28(172.28,565.35) | 3.19(2.69,3.69) |
| Hungary | female | 81379.88(43468.36,124413.40) | 40921.56(31506.46,51608.42) | -0.50 | 967.81(522.21,1477.36) | 359.35(275.40,454.51) | -3.85(-4.01,-3.69) |
| Iceland | female | 155.35(14.71,386.91) | 46.70(9.61,97.84) | -0.70 | 99.54(9.54,246.91) | 15.87(3.20,33.26) | -6.61(-6.89,-6.33) |
| India | female | 873287.57(412967.66,1548566.55) | 4866993.35(3481067.50,6275999.88) | 4.57 | 376.06(178.52,661.61) | 807.44(578.28,1035.25) | 2.88(2.60,3.15) |
| Indonesia | female | 267800.33(119394.53,496201.63) | 882709.48(613761.73,1170407.80) | 2.30 | 470.77(207.86,879.68) | 753.91(525.82,994.51) | 1.45(1.19,1.71) |
| Iran (Islamic Republic of) | female | 164044.90(131333.84,197319.83) | 292658.24(252841.88,334687.00) | 0.78 | 1303.06(1037.16,1566.76) | 808.42(696.93,923.98) | -2.18(-2.51,-1.84) |
| Iraq | female | 73751.98(47028.05,102169.98) | 213543.30(160587.11,277698.41) | 1.90 | 1792.04(1140.54,2485.79) | 1763.25(1337.04,2246.25) | -0.03(-0.36,0.30) |
| Ireland | female | 7648.81(2339.98,14040.00) | 1934.39(1005.04,2960.53) | -0.75 | 339.45(104.14,620.63) | 49.04(25.38,74.59) | -7.07(-7.30,-6.84) |
| Israel | female | 13408.61(7321.79,20325.45) | 7667.40(6190.98,9209.21) | -0.43 | 509.50(279.61,769.11) | 119.91(97.48,143.38) | -5.57(-5.84,-5.31) |
| Italy | female | 213079.11(115669.35,319481.00) | 92140.92(71853.49,113552.02) | -0.57 | 411.77(225.11,616.72) | 109.38(86.90,133.19) | -4.76(-4.88,-4.65) |
| Jamaica | female | 2116.91(918.65,4010.96) | 5003.04(3066.98,7428.50) | 1.36 | 224.83(97.66,425.81) | 322.45(197.65,478.26) | 1.07(0.75,1.40) |
| Japan | female | 187088.93(62806.97,347117.05) | 157554.18(106298.70,215023.73) | -0.16 | 199.45(67.00,370.07) | 92.35(63.12,126.89) | -2.84(-3.21,-2.46) |
| Jordan | female | 9516.93(7538.60,11707.98) | 20461.36(15909.83,26158.81) | 1.15 | 1450.64(1149.82,1779.53) | 681.18(531.97,863.96) | -3.27(-3.77,-2.76) |
| Kazakhstan | female | 60368.97(29370.04,106501.68) | 75317.68(50271.84,104363.61) | 0.25 | 781.99(380.21,1377.24) | 761.38(506.01,1052.02) | -0.89(-1.43,-0.35) |
| Kenya | female | 3814.95(1710.80,7454.86) | 24441.59(12902.39,40300.10) | 5.41 | 88.26(38.97,168.81) | 200.71(105.45,330.76) | 3.64(3.34,3.94) |
| Kiribati | female | 44.26(11.10,117.92) | 115.84(33.76,286.34) | 1.62 | 189.48(48.03,510.49) | 261.65(76.40,658.53) | 0.27(-0.02,0.57) |
| Kuwait | female | 3502.68(3072.52,3966.98) | 6693.26(5480.22,8196.47) | 0.91 | 1321.05(1150.81,1512.64) | 528.24(428.90,646.07) | -2.66(-3.15,-2.17) |
| Kyrgyzstan | female | 10257.28(4033.18,19243.16) | 18795.44(11157.69,28030.74) | 0.83 | 565.91(221.07,1062.43) | 748.40(445.07,1117.04) | 0.53(0.08,0.97) |
| Lao People's Democratic Republic | female | 1938.02(540.08,4840.27) | 7645.03(3826.46,13017.95) | 2.94 | 170.05(48.31,416.86) | 322.53(164.60,539.77) | 2.12(1.84,2.39) |
| Latvia | female | 22626.58(11541.73,35038.02) | 8302.10(5436.42,11601.60) | -0.63 | 970.09(500.11,1491.44) | 315.05(206.30,442.16) | -4.25(-4.74,-3.75) |
| Lebanon | female | 10801.21(7360.20,14440.48) | 21419.92(14157.12,27625.13) | 0.98 | 938.98(637.50,1248.12) | 746.47(492.72,965.46) | -0.91(-1.06,-0.76) |
| Lesotho | female | 857.55(379.30,1701.44) | 3505.14(1679.04,5981.02) | 3.09 | 155.43(68.82,306.70) | 471.72(230.29,800.07) | 5.20(4.69,5.72) |
| Liberia | female | 870.55(310.32,2028.10) | 2984.34(1370.19,5687.35) | 2.43 | 167.12(60.55,383.02) | 278.36(129.30,527.83) | 2.94(2.40,3.47) |
| Libya | female | 8865.75(5480.54,12899.62) | 32922.22(23197.77,44290.51) | 2.71 | 954.33(590.23,1388.57) | 1196.05(853.41,1607.55) | 0.52(0.26,0.79) |
| Lithuania | female | 19459.71(9192.36,31972.63) | 8842.61(5821.92,12446.12) | -0.55 | 696.52(328.91,1147.98) | 234.63(154.89,331.92) | -3.72(-4.24,-3.20) |
| Luxembourg | female | 1241.35(494.30,2156.45) | 345.41(224.33,477.42) | -0.72 | 380.08(150.96,655.24) | 62.36(40.93,86.24) | -6.23(-6.52,-5.95) |
| North Macedonia | female | 14736.34(8570.92,20888.63) | 19746.16(14991.77,25079.42) | 0.34 | 1567.16(907.76,2231.59) | 1222.91(935.38,1546.66) | -1.09(-1.40,-0.78) |
| Madagascar | female | 2461.18(866.90,5660.14) | 12191.73(5302.14,24552.84) | 3.95 | 87.94(31.78,198.10) | 193.55(83.36,385.67) | 2.96(2.80,3.12) |
| Malawi | female | 1406.97(420.55,3803.48) | 4079.83(1665.87,8144.86) | 1.90 | 66.51(20.15,178.01) | 99.59(41.55,198.16) | 1.87(1.61,2.12) |
| Malaysia | female | 53700.92(25123.56,82356.72) | 65929.19(45813.90,89810.13) | 0.23 | 1104.30(509.92,1700.47) | 490.18(340.53,668.56) | -2.73(-2.85,-2.62) |
| Maldives | female | 110.06(31.56,258.65) | 222.49(137.81,325.16) | 1.02 | 264.30(76.73,620.98) | 159.38(98.45,232.99) | -2.67(-3.01,-2.33) |
| Mali | female | 3067.67(756.95,8281.75) | 9707.30(3367.54,21214.37) | 2.16 | 141.18(36.15,383.86) | 221.22(78.56,483.74) | 1.79(1.65,1.94) |
| Malta | female | 1114.75(469.80,1895.03) | 652.13(471.43,839.94) | -0.41 | 465.63(196.82,786.66) | 131.61(96.00,168.38) | -4.43(-4.67,-4.20) |
| Marshall Islands | female | 16.41(3.68,43.26) | 89.63(29.03,194.39) | 4.46 | 178.61(40.50,479.02) | 453.66(146.89,978.70) | 2.88(2.36,3.40) |
| Mauritania | female | 2269.53(937.71,4576.68) | 7173.90(3897.02,10820.29) | 2.16 | 422.05(176.13,849.91) | 675.76(373.65,1011.57) | 1.54(1.36,1.72) |
| Mauritius | female | 2575.33(1315.26,4108.52) | 2707.73(1330.43,4244.23) | 0.05 | 623.19(316.54,995.70) | 294.68(144.95,460.12) | -3.94(-4.60,-3.27) |
| Mexico | female | 89882.02(46996.92,140536.33) | 161673.55(119145.10,211652.87) | 0.80 | 389.94(201.27,614.23) | 257.07(189.69,336.84) | -1.91(-2.20,-1.62) |
| Republic of Moldova | female | 22360.58(10187.92,38287.69) | 16479.44(9511.77,24321.82) | -0.26 | 875.86(399.37,1490.08) | 476.08(273.36,700.66) | -2.04(-2.68,-1.39) |
| Mongolia | female | 4692.40(1973.27,8758.96) | 20021.82(12951.61,28060.24) | 3.27 | 797.71(337.86,1484.16) | 1433.78(927.79,1984.92) | 1.63(1.24,2.01) |
| Montenegro | female | 3132.08(1701.00,4709.33) | 3877.08(2931.42,4826.95) | 0.24 | 913.05(496.64,1371.53) | 717.60(544.65,893.45) | -0.76(-1.12,-0.41) |
| Morocco | female | 50106.02(25423.39,81895.21) | 241539.96(176362.29,319166.55) | 3.82 | 693.42(353.63,1135.97) | 1503.29(1106.37,1954.52) | 2.72(2.39,3.05) |
| Mozambique | female | 1300.15(330.28,3791.92) | 6009.46(2341.19,12725.76) | 3.62 | 40.99(10.73,117.03) | 97.54(39.26,204.52) | 3.98(3.47,4.49) |
| Myanmar | female | 44398.08(14829.60,99834.48) | 137298.86(81308.02,205707.42) | 2.09 | 335.41(113.82,748.22) | 524.47(311.08,784.22) | 1.76(1.47,2.06) |
| Namibia | female | 1100.32(521.59,1987.63) | 3391.25(1867.03,5266.45) | 2.08 | 280.22(133.87,504.01) | 424.60(234.59,655.52) | 1.29(0.95,1.63) |
| Nepal | female | 9166.17(2437.87,22301.56) | 52845.30(29979.77,79928.09) | 4.77 | 182.29(50.75,444.51) | 440.09(254.82,667.60) | 3.53(3.19,3.87) |
| Netherlands | female | 42727.82(20347.70,68439.36) | 15431.29(11328.66,19708.71) | -0.64 | 371.51(178.00,593.32) | 87.89(65.04,111.96) | -5.53(-5.82,-5.25) |
| New Zealand | female | 2640.88(215.10,6763.83) | 1230.15(227.50,2457.34) | -0.53 | 123.18(10.29,312.71) | 30.43(5.69,60.53) | -5.36(-5.63,-5.10) |
| Nicaragua | female | 709.67(241.92,1539.50) | 5080.02(2685.36,8279.74) | 6.16 | 80.07(27.68,173.79) | 210.84(113.41,343.09) | 3.20(2.68,3.73) |
| Niger | female | 1717.28(349.43,5459.27) | 7211.53(1912.91,19576.57) | 3.20 | 123.42(25.82,388.50) | 177.23(48.51,475.63) | 1.14(0.95,1.32) |
| Nigeria | female | 49911.46(20547.74,101079.64) | 237906.62(137823.41,366421.45) | 3.77 | 238.91(99.64,480.98) | 527.45(310.30,794.54) | 2.85(2.54,3.17) |
| Democratic People's Republic of Korea | female | 37091.14(14641.37,71935.73) | 134236.53(80680.57,201071.70) | 2.62 | 381.39(152.76,739.07) | 723.42(431.08,1087.65) | 2.46(2.33,2.60) |
| Northern Mariana Islands | female | 42.26(14.30,101.59) | 61.33(34.11,97.98) | 0.45 | 428.36(142.22,1034.22) | 235.77(130.27,376.41) | -2.12(-2.71,-1.53) |
| Norway | female | 8921.02(2379.21,17324.58) | 1449.49(553.42,2526.26) | -0.84 | 228.21(60.65,439.75) | 28.25(10.78,49.05) | -7.46(-7.73,-7.19) |
| Oman | female | 4281.69(2241.25,6986.31) | 11949.13(9185.72,14808.35) | 1.79 | 1411.91(742.74,2284.79) | 1678.70(1295.55,2068.58) | 0.17(-0.46,0.81) |
| Pakistan | female | 79873.45(25468.13,170334.63) | 593476.39(366131.80,864216.83) | 6.43 | 292.66(93.74,627.71) | 986.52(617.15,1419.79) | 4.52(4.29,4.75) |
| Palestine | female | 3452.66(1578.88,5836.08) | 12580.94(9435.80,15561.98) | 2.64 | 732.94(334.19,1239.73) | 1057.80(791.00,1313.51) | 0.60(0.14,1.06) |
| Panama | female | 1778.84(772.87,3356.85) | 2950.71(1764.26,4319.25) | 0.66 | 232.91(101.01,440.33) | 137.70(82.93,201.66) | -1.31(-1.55,-1.07) |
| Papua New Guinea | female | 844.73(152.75,2780.08) | 4128.43(921.33,10862.83) | 3.89 | 85.80(15.82,279.79) | 159.51(36.26,417.38) | 2.25(2.08,2.42) |
| Paraguay | female | 2191.77(882.19,4264.43) | 5531.19(3300.23,8580.71) | 1.52 | 181.62(73.36,353.68) | 187.20(111.65,289.63) | -0.11(-0.38,0.15) |
| Peru | female | 21339.85(9788.56,37471.32) | 38114.47(25024.21,54421.15) | 0.79 | 322.88(146.82,569.83) | 224.01(147.12,319.65) | -1.60(-2.12,-1.09) |
| Philippines | female | 33236.16(16080.63,57268.36) | 200220.43(129933.27,277437.58) | 5.02 | 226.74(108.98,384.62) | 458.80(298.87,630.99) | 2.73(1.84,3.63) |
| Poland | female | 257124.55(148502.82,374139.59) | 145992.62(115080.04,185823.78) | -0.43 | 1001.60(577.55,1455.50) | 353.72(278.72,448.04) | -4.11(-4.29,-3.92) |
| Portugal | female | 25640.19(8684.32,48716.77) | 7947.47(4676.85,11489.21) | -0.69 | 323.77(111.12,611.20) | 57.42(33.44,82.68) | -6.50(-6.89,-6.12) |
| Puerto Rico | female | 2371.72(21.01,7674.30) | 1516.58(328.80,2806.98) | -0.36 | 122.02(1.08,392.90) | 42.28(8.91,78.14) | -4.50(-5.09,-3.91) |
| Qatar | female | 1065.68(844.71,1314.32) | 2948.11(2288.54,3702.14) | 1.77 | 2467.52(1919.23,2997.11) | 1614.02(1318.23,1976.78) | -1.54(-1.92,-1.16) |
| Romania | female | 132278.46(69222.66,209828.36) | 97009.77(74406.52,121671.45) | -0.27 | 876.42(456.16,1389.92) | 451.19(345.42,569.22) | -2.97(-3.36,-2.57) |
| Russian Federation | female | 1095893.26(474595.15,1806489.59) | 586883.84(332762.79,870573.23) | -0.46 | 930.52(404.74,1545.37) | 402.33(227.85,595.07) | -3.24(-3.88,-2.60) |
| Rwanda | female | 3165.87(924.62,7942.79) | 7437.60(3017.48,14806.71) | 1.35 | 187.29(56.01,464.77) | 216.33(89.97,423.64) | 0.04(-0.39,0.47) |
| Saint Lucia | female | 228.53(74.51,483.13) | 390.97(167.45,662.20) | 0.71 | 471.26(153.59,1000.06) | 344.97(148.24,583.39) | -1.87(-2.30,-1.45) |
| Saint Vincent and the Grenadines | female | 190.62(52.61,426.67) | 348.10(136.42,601.74) | 0.83 | 480.56(132.64,1074.40) | 535.29(210.43,927.07) | -0.15(-0.46,0.17) |
| Samoa | female | 121.19(27.68,327.62) | 280.01(73.93,674.58) | 1.31 | 264.27(60.94,708.23) | 365.84(97.85,877.80) | 0.84(0.74,0.94) |
| Sao Tome and Principe | female | 55.01(21.18,114.42) | 301.77(142.87,510.00) | 4.49 | 163.15(62.91,338.52) | 528.21(253.21,893.46) | 4.40(4.13,4.67) |
| Saudi Arabia | female | 28840.35(14806.34,46175.07) | 167391.20(129192.41,215035.16) | 4.80 | 1083.70(569.45,1717.66) | 1804.65(1436.93,2258.05) | 1.19(0.46,1.93) |
| Senegal | female | 3343.52(1153.72,7594.90) | 13887.70(6281.74,25289.52) | 3.15 | 204.47(71.99,458.60) | 351.02(162.07,638.19) | 1.72(1.52,1.91) |
| Serbia | female | 70798.17(37947.88,105467.88) | 68784.91(53313.73,86444.94) | -0.03 | 1246.97(666.96,1861.21) | 789.84(613.45,993.32) | -2.04(-2.46,-1.62) |
| Seychelles | female | 124.68(66.22,197.12) | 181.17(94.89,275.16) | 0.45 | 396.61(210.33,625.66) | 318.35(166.88,487.49) | -0.91(-1.05,-0.77) |
| Sierra Leone | female | 1399.32(438.76,3601.34) | 5605.96(2344.83,11428.83) | 3.01 | 147.47(47.56,372.61) | 293.64(124.50,589.01) | 3.02(2.73,3.31) |
| Singapore | female | 7398.36(2581.74,13065.56) | 6063.15(3925.22,8281.70) | -0.18 | 616.29(212.94,1091.88) | 151.22(97.90,206.56) | -5.22(-5.63,-4.81) |
| Slovakia | female | 41313.75(22475.88,61028.41) | 22726.75(16908.92,29014.27) | -0.45 | 1199.74(653.96,1762.48) | 421.55(314.08,537.48) | -3.89(-4.08,-3.70) |
| Slovenia | female | 8935.39(4791.25,14458.90) | 3576.19(2632.70,4758.98) | -0.60 | 606.25(328.29,979.37) | 142.98(107.52,187.31) | -5.61(-5.84,-5.38) |
| Solomon Islands | female | 113.67(19.75,347.56) | 707.78(188.46,1911.79) | 5.23 | 155.16(27.70,477.27) | 385.94(104.95,1044.48) | 3.00(2.83,3.17) |
| Somalia | female | 633.33(117.50,2010.40) | 2135.98(465.25,6319.68) | 2.37 | 43.19(8.39,137.78) | 54.76(12.20,158.21) | 1.36(1.05,1.66) |
| South Africa | female | 56727.30(41516.24,74198.22) | 119677.16(93514.32,147295.08) | 1.11 | 440.32(322.52,573.17) | 461.05(360.96,568.27) | 0.43(0.00,0.86) |
| Republic of Korea | female | 171359.02(94422.01,255809.10) | 98822.75(80080.12,122549.59) | -0.42 | 996.34(547.99,1493.70) | 204.72(166.17,254.28) | -6.02(-6.43,-5.61) |
| South Sudan | female | 1281.92(422.59,2996.98) | 3217.59(1264.08,6293.75) | 1.51 | 117.78(39.06,271.78) | 171.58(67.67,328.56) | 1.67(1.48,1.86) |
| Spain | female | 71712.30(27906.07,126220.65) | 29385.97(19910.86,40053.26) | -0.59 | 230.10(90.78,405.86) | 53.99(36.65,73.19) | -5.09(-5.35,-4.84) |
| Sri Lanka | female | 14083.49(5770.13,26630.27) | 39930.00(24401.32,59315.48) | 1.84 | 263.38(108.25,496.07) | 289.16(176.61,430.14) | 0.41(0.01,0.81) |
| Sudan | female | 17983.20(6097.18,40724.16) | 122335.41(67976.78,192139.31) | 5.80 | 378.06(131.64,857.03) | 1293.32(732.47,1994.67) | 4.61(4.44,4.79) |
| Suriname | female | 891.73(286.51,1742.17) | 1727.84(845.64,2875.24) | 0.94 | 647.02(207.23,1265.26) | 532.67(260.31,887.03) | -0.97(-1.33,-0.60) |
| Eswatini | female | 352.63(160.30,634.32) | 1463.59(746.55,2418.67) | 3.15 | 210.29(96.95,374.26) | 425.93(218.83,698.96) | 2.79(2.32,3.27) |
| Sweden | female | 14727.03(2608.80,30950.58) | 2660.28(711.24,5312.63) | -0.82 | 169.67(30.27,357.23) | 23.84(6.34,47.46) | -6.74(-7.13,-6.34) |
| Switzerland | female | 17421.14(7545.09,28856.45) | 5083.54(3368.09,7078.26) | -0.71 | 275.26(119.75,452.67) | 50.67(33.78,68.90) | -5.94(-6.12,-5.76) |
| Syrian Arab Republic | female | 52100.92(36003.04,70229.62) | 92438.20(67129.29,125558.08) | 0.77 | 1879.36(1308.09,2511.57) | 1589.06(1168.68,2120.16) | -1.02(-1.25,-0.79) |
| Taiwan (Province of China) | female | 38392.34(20500.05,61270.30) | 42304.21(33218.77,52954.53) | 0.10 | 516.21(272.35,825.52) | 209.20(163.56,262.09) | -3.25(-3.45,-3.04) |
| Tajikistan | female | 7848.68(2689.32,16800.98) | 35544.78(18072.31,56830.67) | 3.53 | 502.55(171.95,1075.35) | 1459.21(738.12,2330.59) | 3.61(3.37,3.84) |
| United Republic of Tanzania | female | 3669.76(1312.39,8368.41) | 20566.27(10391.62,36244.46) | 4.60 | 63.70(23.14,142.82) | 156.92(80.06,274.20) | 3.83(3.57,4.09) |
| Thailand | female | 76827.47(36011.56,129090.15) | 167501.98(119823.90,223773.22) | 1.18 | 392.94(186.42,661.13) | 309.69(220.90,415.51) | -1.31(-1.79,-0.83) |
| Bahamas | female | 431.45(119.05,835.07) | 649.20(195.53,1233.70) | 0.50 | 471.88(127.24,918.87) | 298.71(89.55,568.17) | -1.76(-2.00,-1.53) |
| Gambia | female | 239.27(74.28,593.99) | 2002.54(963.39,3517.57) | 7.37 | 140.28(44.21,336.45) | 393.95(188.83,687.87) | 3.75(3.54,3.97) |
| Timor-Leste | female | 117.75(28.49,317.82) | 1142.68(464.51,2143.00) | 8.70 | 80.19(19.73,210.86) | 278.61(115.22,519.99) | 5.56(4.96,6.17) |
| Togo | female | 1392.30(507.05,2861.43) | 7202.04(3376.78,12523.74) | 4.17 | 198.46(74.93,399.03) | 330.97(155.53,570.48) | 1.78(1.44,2.12) |
| Tonga | female | 38.90(9.70,103.54) | 89.02(27.27,195.28) | 1.29 | 131.87(32.96,352.62) | 207.63(63.81,454.89) | 1.30(1.11,1.48) |
| Trinidad and Tobago | female | 4166.12(859.93,8258.76) | 4583.64(1553.30,8268.90) | 0.10 | 935.50(192.53,1858.08) | 480.31(162.87,867.82) | -3.19(-3.63,-2.76) |
| Tunisia | female | 20931.28(13483.44,29008.59) | 51954.27(36033.56,71400.18) | 1.48 | 853.46(545.26,1180.50) | 809.62(559.17,1109.96) | -0.35(-0.72,0.01) |
| Turkey | female | 134220.45(95718.10,170180.60) | 225358.83(174126.58,286869.26) | 0.68 | 706.08(507.52,897.37) | 486.16(375.82,618.29) | -1.36(-1.60,-1.13) |
| Turkmenistan | female | 16631.01(6975.97,27918.81) | 31836.68(19227.33,47021.70) | 0.91 | 1492.47(626.85,2521.15) | 1472.16(886.83,2160.84) | -0.74(-1.12,-0.36) |
| Uganda | female | 1984.19(539.98,5139.61) | 13734.46(6426.59,25137.78) | 5.92 | 60.32(17.05,154.41) | 172.99(82.22,310.27) | 4.18(3.89,4.46) |
| Ukraine | female | 488978.89(230505.07,772049.74) | 342594.06(209929.42,510911.44) | -0.30 | 1050.63(491.72,1653.12) | 713.99(432.35,1061.40) | -1.78(-2.22,-1.33) |
| United Arab Emirates | female | 3510.45(2642.02,4557.82) | 15087.20(10993.65,20243.51) | 3.30 | 2164.01(1662.49,2822.32) | 1154.83(861.74,1523.03) | -1.92(-2.74,-1.10) |
| United Kingdom | female | 224127.38(92476.50,374471.13) | 49839.51(33391.51,67249.20) | -0.78 | 429.15(178.35,719.32) | 77.17(51.99,103.78) | -6.45(-6.67,-6.22) |
| United States of America | female | 546996.86(212183.74,967359.05) | 223756.68(121227.33,337513.35) | -0.59 | 303.19(118.21,531.81) | 80.02(43.19,120.89) | -4.99(-5.29,-4.69) |
| Uruguay | female | 5328.62(1782.56,11111.91) | 3328.01(1711.47,5201.60) | -0.38 | 252.68(85.56,521.67) | 111.84(58.01,175.19) | -3.10(-3.31,-2.88) |
| Uzbekistan | female | 61029.77(23598.90,108592.23) | 236980.28(149496.33,331417.80) | 2.88 | 934.66(361.34,1666.69) | 2354.64(1479.41,3286.34) | 2.82(2.18,3.46) |
| Vanuatu | female | 54.62(11.74,155.04) | 307.86(81.58,733.38) | 4.64 | 168.46(35.33,474.44) | 343.48(92.87,815.36) | 1.97(1.69,2.25) |
| Venezuela (Bolivarian Republic of) | female | 34280.73(15064.09,56683.23) | 71787.09(47347.33,104444.56) | 1.09 | 621.34(269.77,1027.40) | 461.25(304.43,670.38) | -1.31(-1.57,-1.06) |
| Viet nam | female | 43935.43(16256.30,90666.17) | 193502.54(123386.30,272163.19) | 3.40 | 190.01(70.41,391.85) | 372.87(238.30,521.48) | 2.97(2.46,3.47) |
| Virginia | female | 13165.29(5679.21,22865.67) | 4296.95(1573.24,7581.51) | -0.67 | 325.42(141.57,567.66) | 59.59(21.89,104.55) | -6.11(-6.54,-5.67) |
| Yemen | female | 9040.52(2445.86,23555.57) | 84495.01(40605.81,139243.20) | 8.35 | 337.82(92.43,875.89) | 1150.90(552.94,1874.32) | 4.75(4.49,5.00) |
| Zambia | female | 1982.10(746.92,4212.73) | 11910.61(5956.19,20246.98) | 5.01 | 133.41(50.74,284.91) | 323.33(162.70,546.26) | 3.26(2.92,3.60) |
| Zimbabwe | female | 3372.77(1538.70,6021.85) | 11062.71(5029.89,19737.32) | 2.28 | 163.90(75.30,291.58) | 273.05(125.63,483.93) | 2.85(2.38,3.32) |
| Monaco | female | 65.89(6.68,144.64) | 51.73(29.45,76.24) | -0.21 | 158.04(15.81,346.89) | 101.76(58.23,151.03) | -1.09(-1.75,-0.43) |
| San Marino | female | 24.51(5.53,50.14) | 22.20(8.69,40.97) | -0.09 | 133.78(30.69,274.00) | 63.55(24.93,116.05) | -2.73(-3.09,-2.36) |
| Saint Kitts and Nevis | female | 101.85(36.89,203.52) | 63.20(28.88,105.44) | -0.38 | 507.32(182.69,1006.23) | 188.88(87.22,313.49) | -3.64(-4.07,-3.21) |
| Cook Islands | female | 10.07(3.85,22.94) | 11.70(2.80,27.00) | 0.16 | 158.01(60.25,363.52) | 97.22(23.21,225.98) | -1.81(-2.16,-1.45) |
| Nauru | female | 8.01(2.08,22.88) | 11.37(3.20,26.97) | 0.42 | 338.80(87.90,991.08) | 369.24(105.20,866.47) | -0.27(-0.53,-0.01) |
| Niue | female | 2.58(0.93,5.70) | 2.48(0.61,5.63) | -0.04 | 215.33(78.67,477.13) | 221.58(54.14,506.02) | -0.45(-0.66,-0.24) |
| Palau | female | 12.35(0.15,38.05) | 21.22(0.11,53.85) | 0.72 | 230.54(2.74,714.14) | 195.95(1.03,499.65) | 0.36(-0.48,1.20) |
| Tokelau | female | 2.57(0.07,9.44) | 1.71(0.01,4.73) | -0.33 | 360.15(10.01,1324.51) | 258.65(1.49,702.38) | -1.61(-1.93,-1.30) |
| Tuvalu | female | 6.15(1.50,17.63) | 15.43(6.01,33.20) | 1.51 | 150.36(36.30,431.11) | 284.11(110.86,610.11) | 1.85(1.54,2.16) |
| Afghanistan | male | 21212.72(5008.25,55750.79) | 82947.72(35428.43,154669.44) | 2.91 | 564.59(134.57,1478.00) | 1078.51(471.39,1983.17) | 2.53(2.03,3.04) |
| Albania | male | 8201.82(3959.19,13249.27) | 14369.74(10197.96,20051.05) | 0.75 | 841.54(406.54,1356.10) | 746.44(531.37,1037.08) | 0.06(-0.22,0.34) |
| Algeria | male | 122885.80(77462.71,171703.09) | 222387.74(149055.68,309866.08) | 0.81 | 1959.80(1258.23,2739.79) | 1251.21(846.02,1723.25) | -2.01(-2.22,-1.79) |
| American Samoa | male | 62.45(20.16,157.83) | 81.07(28.44,162.77) | 0.30 | 432.88(139.11,1101.96) | 323.15(113.05,646.88) | -1.30(-1.57,-1.04) |
| Andorra | male | 80.97(25.71,152.04) | 60.93(32.71,93.89) | -0.25 | 277.81(89.24,519.56) | 88.48(47.84,136.55) | -3.84(-4.05,-3.64) |
| Angola | male | 5252.16(1523.46,13025.07) | 42375.45(21535.82,71980.58) | 7.07 | 235.30(69.93,577.98) | 720.58(361.31,1204.65) | 3.99(3.68,4.29) |
| Antigua and Barbuda | male | 195.71(52.94,379.10) | 211.74(81.81,366.38) | 0.08 | 859.93(232.97,1668.75) | 433.46(167.64,749.15) | -2.57(-2.89,-2.25) |
| Argentina | male | 122446.90(48945.35,212733.96) | 84783.95(52928.77,119595.74) | -0.31 | 830.65(331.46,1447.21) | 359.00(224.14,506.88) | -3.13(-3.42,-2.84) |
| Armenia | male | 21184.00(10726.74,32374.70) | 28129.88(19661.26,36724.18) | 0.33 | 1774.00(891.03,2737.22) | 1601.04(1112.79,2096.79) | -0.54(-0.80,-0.28) |
| Australia | male | 21591.65(2288.15,51729.53) | 10609.10(2640.38,19324.34) | -0.51 | 244.04(25.79,582.46) | 59.05(14.63,106.73) | -5.31(-5.64,-4.99) |
| Austria | male | 42286.81(21343.36,64713.45) | 14818.23(11123.44,18872.92) | -0.65 | 940.85(475.73,1441.45) | 197.81(148.75,251.14) | -5.73(-5.93,-5.54) |
| Azerbaijan | male | 36710.90(17091.37,62903.88) | 92742.15(53991.60,137329.44) | 1.53 | 1650.35(772.81,2839.27) | 2148.85(1263.95,3167.60) | 0.68(0.40,0.97) |
| Bahrain | male | 4288.05(3471.26,5129.02) | 7826.90(6129.89,9970.98) | 0.83 | 3290.22(2674.30,3911.37) | 1057.20(833.58,1340.50) | -4.42(-4.70,-4.13) |
| Bangladesh | male | 112527.57(33775.08,252674.79) | 762096.73(466628.47,1108956.82) | 5.77 | 399.42(120.63,897.17) | 1092.53(669.89,1582.32) | 3.87(3.67,4.07) |
| Barbados | male | 1005.40(290.06,1835.07) | 1026.09(454.56,1674.79) | 0.02 | 857.57(249.88,1563.68) | 478.72(213.12,785.02) | -2.71(-3.10,-2.33) |
| Belarus | male | 101010.76(54841.54,151316.32) | 91231.33(62527.40,126294.96) | -0.10 | 2056.60(1099.07,3091.09) | 1512.29(1035.78,2083.78) | -1.14(-1.85,-0.42) |
| Belgium | male | 50055.28(24007.47,77979.55) | 17277.12(13120.82,21668.42) | -0.65 | 796.15(382.68,1237.57) | 181.81(138.82,227.41) | -5.19(-5.38,-4.99) |
| Belize | male | 201.49(47.53,438.57) | 814.52(320.92,1396.47) | 3.04 | 416.57(97.81,905.06) | 535.24(209.33,925.61) | -0.34(-1.02,0.35) |
| Benin | male | 2496.47(782.25,5770.00) | 11473.88(5028.29,21091.14) | 3.60 | 251.73(79.80,584.62) | 459.46(202.49,845.46) | 2.02(1.73,2.31) |
| Bermuda | male | 168.55(35.86,432.85) | 64.21(10.71,126.67) | -0.62 | 594.56(125.82,1538.31) | 116.60(19.50,229.64) | -5.90(-6.40,-5.39) |
| Bhutan | male | 259.15(63.01,651.20) | 2092.77(1146.57,3189.57) | 7.08 | 189.69(48.83,471.84) | 684.62(381.67,1039.18) | 5.55(5.16,5.94) |
| Bolivia (Plurinational State of) | male | 12754.72(4648.01,23362.07) | 24282.55(13680.92,36613.80) | 0.90 | 789.00(288.08,1456.18) | 555.36(310.83,834.02) | -1.33(-1.73,-0.93) |
| Bosnia and Herzegovina | male | 21971.53(11395.64,34146.72) | 27823.08(20987.11,36094.87) | 0.27 | 1180.35(611.01,1845.69) | 1089.49(825.66,1409.47) | -0.40(-0.67,-0.12) |
| Botswana | male | 1558.86(710.23,2765.46) | 7265.81(4451.70,10852.92) | 3.66 | 561.91(260.94,991.59) | 1062.19(667.77,1551.77) | 1.55(0.67,2.45) |
| Brazil | male | 328783.88(155026.19,558412.64) | 372524.54(264174.84,492614.69) | 0.13 | 695.24(326.20,1190.72) | 332.67(236.26,440.01) | -2.60(-2.83,-2.36) |
| Brunei Darussalam | male | 311.02(110.41,618.04) | 405.06(115.70,720.32) | 0.30 | 512.54(178.89,1030.25) | 237.04(68.51,424.81) | -2.23(-3.16,-1.29) |
| Bulgaria | male | 135947.79(76833.33,200913.26) | 85002.80(63987.07,108848.06) | -0.37 | 2455.54(1383.52,3633.51) | 1474.13(1111.88,1892.82) | -2.55(-2.96,-2.14) |
| Burkina Faso | male | 3360.45(833.42,8858.89) | 15556.63(5224.28,33538.20) | 3.63 | 157.44(39.16,409.80) | 344.16(118.36,726.98) | 3.27(3.07,3.47) |
| Burundi | male | 3434.62(895.56,9102.81) | 6790.39(2141.85,15737.75) | 0.98 | 297.17(79.73,780.88) | 253.29(82.91,578.79) | -0.82(-1.19,-0.46) |
| Cambodia | male | 5829.43(1598.36,14857.31) | 28606.19(13476.28,49227.51) | 3.91 | 276.01(76.45,697.21) | 532.11(251.69,901.93) | 2.67(2.42,2.91) |
| Cameroon | male | 10917.98(4671.09,19939.86) | 66408.81(37598.87,99544.68) | 5.08 | 472.14(201.23,853.52) | 1015.47(582.94,1488.32) | 2.67(2.43,2.92) |
| Canada | male | 52080.62(13291.08,103865.51) | 23476.69(11141.29,37788.50) | -0.55 | 360.39(91.81,720.79) | 79.95(37.75,128.50) | -5.69(-5.95,-5.42) |
| Cabo Verde | male | 368.66(149.23,699.25) | 2554.14(1723.99,3341.70) | 5.93 | 394.70(158.46,753.87) | 1314.51(898.86,1710.37) | 3.36(2.83,3.89) |
| Central African Republic | male | 2346.67(588.53,5748.52) | 6160.61(1858.87,14187.22) | 1.63 | 377.02(95.00,923.11) | 509.00(158.48,1150.61) | 1.01(0.78,1.24) |
| Chad | male | 2284.49(565.26,6407.52) | 10372.06(3504.03,23210.41) | 3.54 | 164.75(40.79,459.40) | 320.22(109.48,709.34) | 2.50(2.33,2.67) |
| Chile | male | 31491.72(15804.49,50531.97) | 43870.48(35611.39,52690.07) | 0.39 | 677.58(337.46,1089.57) | 404.31(328.96,484.84) | -1.58(-1.71,-1.45) |
| China | male | 3662816.00(1682420.45,6175236.17) | 13223362.01(10095807.99,16434452.95) | 2.61 | 876.66(404.11,1468.49) | 1411.70(1090.15,1738.55) | 2.08(1.75,2.41) |
| Colombia | male | 62920.35(29892.98,104267.10) | 93945.78(64430.67,131882.76) | 0.49 | 679.41(321.13,1131.84) | 389.00(266.59,545.03) | -2.16(-2.35,-1.98) |
| Comoros | male | 161.92(49.29,370.93) | 618.61(281.51,1078.90) | 2.82 | 144.68(46.19,330.34) | 261.36(120.87,459.04) | 1.77(1.54,2.01) |
| Congo | male | 3744.98(1270.46,7588.24) | 14523.26(8025.63,23194.53) | 2.88 | 703.46(244.47,1412.45) | 1011.94(561.38,1586.84) | 0.93(0.57,1.28) |
| Costa Rica | male | 3486.67(1629.66,6000.70) | 8513.01(5758.22,11900.94) | 1.44 | 389.88(182.76,674.58) | 352.56(238.95,490.82) | -0.55(-0.76,-0.34) |
| Côte d'Ivoire | male | 10397.70(3449.96,23521.20) | 42832.98(19990.88,73172.58) | 3.12 | 420.12(142.35,939.65) | 667.90(318.46,1132.22) | 1.32(0.91,1.72) |
| Croatia | male | 45665.58(24705.68,67578.82) | 22407.78(16566.91,29413.38) | -0.51 | 1732.87(929.48,2566.08) | 632.93(471.50,830.36) | -3.67(-3.90,-3.44) |
| Cuba | male | 43898.25(17017.46,77163.89) | 44253.92(22164.57,72025.89) | 0.01 | 861.77(333.71,1511.58) | 510.40(256.31,832.18) | -1.97(-2.24,-1.70) |
| Cyprus | male | 3170.50(1317.07,5243.65) | 3143.85(2388.09,4022.65) | -0.01 | 842.64(346.33,1396.82) | 358.11(272.82,457.62) | -3.48(-3.85,-3.10) |
| Czechia | male | 134017.68(72964.50,196807.64) | 46194.30(34805.66,60067.23) | -0.66 | 2357.29(1289.07,3463.68) | 524.28(395.83,678.35) | -5.22(-5.33,-5.10) |
| Democratic Republic of the Congo | male | 22594.72(6335.70,56189.17) | 71212.91(28068.26,138863.16) | 2.15 | 283.07(79.77,704.57) | 387.12(153.22,745.02) | 0.64(-0.02,1.31) |
| Denmark | male | 29985.11(12635.63,49877.32) | 6215.11(3875.48,8797.68) | -0.79 | 886.91(369.67,1468.97) | 125.07(77.80,176.89) | -6.99(-7.22,-6.76) |
| Djibouti | male | 352.39(109.83,783.01) | 4147.76(2000.56,6597.52) | 10.77 | 403.74(131.46,900.96) | 1109.08(547.29,1738.68) | 3.99(3.50,4.48) |
| Dominica | male | 159.96(60.57,301.81) | 206.95(90.86,358.18) | 0.29 | 555.98(211.54,1062.04) | 471.19(206.15,819.85) | -0.90(-1.16,-0.64) |
| Dominican Republic | male | 6712.51(2038.57,15141.97) | 45462.96(21075.67,81569.44) | 5.77 | 328.62(99.77,739.93) | 952.67(439.22,1708.86) | 4.62(4.32,4.92) |
| Ecuador | male | 14501.64(6845.67,24712.80) | 34128.79(21877.32,48111.15) | 1.35 | 492.57(231.77,843.13) | 451.75(288.01,636.79) | 0.01(-0.31,0.33) |
| Egypt | male | 545189.99(434195.91,672861.78) | 1179522.84(839326.34,1582598.38) | 1.16 | 3319.57(2631.33,4054.91) | 3090.34(2226.39,4107.96) | -0.22(-0.33,-0.11) |
| El Salvador | male | 6115.12(2244.80,11437.05) | 13020.83(8069.83,20062.38) | 1.13 | 405.22(147.56,757.66) | 516.21(320.83,796.01) | 0.82(0.54,1.09) |
| Equatorial Guinea | male | 315.19(77.01,854.20) | 2174.92(1244.30,3380.39) | 5.90 | 327.14(80.28,876.90) | 949.56(539.22,1434.04) | 4.16(3.96,4.36) |
| Eritrea | male | 1908.74(520.46,4785.64) | 10374.95(4118.91,19849.61) | 4.44 | 358.04(101.21,894.33) | 713.90(287.78,1336.54) | 2.01(1.69,2.32) |
| Estonia | male | 7303.71(2822.88,13462.68) | 1201.67(391.15,2182.90) | -0.84 | 963.01(370.08,1782.66) | 127.01(41.30,231.41) | -7.50(-8.32,-6.68) |
| Ethiopia | male | 16890.31(4563.11,44343.38) | 52599.12(24321.62,97179.06) | 2.11 | 145.11(40.26,379.37) | 229.25(106.39,423.48) | 1.80(1.44,2.17) |
| Micronesia (Federated States of) | male | 189.44(40.01,527.19) | 468.67(141.36,1060.43) | 1.47 | 669.35(145.44,1844.16) | 1108.22(332.57,2499.96) | 1.69(1.49,1.89) |
| Fiji | male | 1345.57(317.03,3711.58) | 3661.46(1123.00,7848.14) | 1.72 | 588.43(138.18,1627.15) | 894.26(271.38,1918.14) | 1.22(0.68,1.76) |
| Finland | male | 13826.86(1599.65,29816.14) | 3006.15(593.26,6203.37) | -0.78 | 482.05(56.10,1032.97) | 60.02(11.97,124.41) | -7.20(-7.72,-6.68) |
| France | male | 166061.93(75876.65,268991.75) | 67862.76(49494.25,87250.96) | -0.59 | 496.55(227.52,802.68) | 128.53(94.12,165.15) | -4.60(-4.83,-4.36) |
| Gabon | male | 2051.27(819.03,3841.29) | 6893.79(4264.01,10102.85) | 2.36 | 763.50(306.39,1421.29) | 1275.78(785.87,1839.70) | 1.63(1.30,1.95) |
| Georgia | male | 49361.79(25797.84,83406.50) | 32776.17(22692.89,43650.12) | -0.34 | 1979.66(1032.14,3331.49) | 1406.84(972.65,1868.98) | -1.55(-1.75,-1.35) |
| Germany | male | 512762.91(246852.73,806836.80) | 163709.64(121053.71,209243.00) | -0.68 | 1052.70(507.06,1657.94) | 207.66(154.08,264.80) | -5.58(-5.84,-5.31) |
| Ghana | male | 14968.46(6525.27,27038.39) | 83837.12(53075.94,119727.66) | 4.60 | 455.52(200.70,814.79) | 1052.51(666.75,1486.95) | 3.42(3.15,3.69) |
| Greece | male | 58354.70(28397.48,92811.55) | 36697.02(28254.34,45351.59) | -0.37 | 866.23(422.30,1375.92) | 405.18(312.80,499.43) | -2.72(-3.05,-2.39) |
| Greenland | male | 76.14(14.63,199.01) | 57.94(4.67,164.76) | -0.24 | 359.16(69.24,961.49) | 149.12(12.00,424.22) | -3.36(-3.68,-3.04) |
| Grenada | male | 285.39(81.11,612.46) | 421.80(169.26,706.60) | 0.48 | 967.48(273.47,2072.10) | 754.33(302.07,1263.69) | -1.25(-1.44,-1.06) |
| Guam | male | 223.80(36.18,596.76) | 391.68(157.90,682.95) | 0.75 | 481.91(75.93,1300.57) | 412.91(166.43,714.53) | -0.24(-0.94,0.45) |
| Guatemala | male | 6088.72(1989.27,12983.93) | 21971.03(12756.72,32636.55) | 2.61 | 306.05(100.03,652.19) | 406.57(235.13,604.70) | 0.40(-0.05,0.86) |
| Guinea | male | 2843.25(840.45,7090.99) | 11245.90(4535.79,22195.70) | 2.96 | 169.45(51.05,419.05) | 383.66(155.96,753.94) | 3.39(3.23,3.56) |
| Guinea-Bissau | male | 824.50(223.83,2063.41) | 2555.96(1061.39,4873.85) | 2.10 | 391.53(108.78,960.30) | 663.87(283.94,1249.18) | 2.08(2.00,2.16) |
| Guyana | male | 4051.70(1119.26,7973.79) | 4468.05(1943.23,8049.38) | 0.10 | 1963.30(536.06,3878.52) | 1379.13(594.07,2493.68) | -1.05(-1.25,-0.85) |
| Haiti | male | 5088.41(1360.68,12774.91) | 15267.58(5450.20,32257.63) | 2.00 | 299.00(81.00,756.17) | 424.60(152.19,883.61) | 1.51(1.30,1.72) |
| Honduras | male | 2734.18(823.45,6164.63) | 15268.36(7893.81,24213.28) | 4.58 | 247.93(74.60,556.55) | 525.76(270.89,824.43) | 3.02(2.73,3.30) |
| Hungary | male | 125304.58(69187.00,188583.57) | 53275.40(40549.63,67622.55) | -0.57 | 2072.52(1141.57,3128.27) | 715.48(546.29,909.01) | -4.03(-4.21,-3.86) |
| Iceland | male | 281.54(24.25,695.93) | 115.97(23.59,242.46) | -0.59 | 217.71(18.77,536.31) | 46.49(9.48,96.44) | -5.39(-5.59,-5.19) |
| India | male | 2014446.57(945245.86,3433411.18) | 8589277.43(6277283.69,10884331.75) | 3.26 | 758.80(357.14,1281.06) | 1425.15(1046.38,1812.78) | 2.57(2.40,2.75) |
| Indonesia | male | 368798.39(159988.72,658682.85) | 1268985.06(878779.41,1748416.54) | 2.44 | 670.28(288.07,1216.96) | 1098.28(770.43,1490.13) | 1.71(1.50,1.92) |
| Iran (Islamic Republic of) | male | 288653.98(234083.92,341890.87) | 451837.48(393436.67,514913.54) | 0.57 | 1959.26(1594.93,2310.32) | 1156.64(1005.37,1318.78) | -2.11(-2.34,-1.87) |
| Iraq | male | 113576.21(76597.97,155039.49) | 325311.25(234782.59,423822.12) | 1.86 | 2754.31(1861.30,3770.80) | 2638.43(1964.21,3346.01) | -0.28(-0.53,-0.04) |
| Ireland | male | 13085.99(3958.31,23925.00) | 3435.95(1731.62,5213.37) | -0.74 | 715.18(217.01,1305.43) | 101.01(50.92,151.96) | -7.11(-7.34,-6.87) |
| Israel | male | 19430.02(10641.23,29154.62) | 12378.65(10158.73,14608.04) | -0.36 | 885.85(487.33,1324.70) | 241.93(199.27,284.64) | -5.05(-5.26,-4.84) |
| Italy | male | 303676.50(166949.66,446593.65) | 124412.06(100499.20,150628.67) | -0.59 | 822.70(454.54,1208.96) | 216.90(175.30,262.10) | -4.74(-4.84,-4.65) |
| Jamaica | male | 2665.51(1167.26,4827.43) | 5735.19(3549.57,8446.37) | 1.15 | 324.28(142.07,589.27) | 399.45(247.72,587.28) | 0.77(0.31,1.23) |
| Japan | male | 253052.30(84241.53,460235.47) | 230648.24(160291.61,314917.66) | -0.09 | 341.74(113.58,623.83) | 185.83(129.48,253.51) | -2.16(-2.40,-1.92) |
| Jordan | male | 12362.28(9503.68,15399.95) | 36555.89(27645.83,47383.03) | 1.96 | 1584.77(1221.60,1962.46) | 931.07(709.27,1201.63) | -2.20(-2.38,-2.02) |
| Kazakhstan | male | 86940.72(43939.07,145097.46) | 107908.63(70725.08,151747.31) | 0.24 | 1635.98(833.23,2749.36) | 1453.90(966.13,2042.65) | -1.13(-1.76,-0.50) |
| Kenya | male | 7067.18(3000.44,13714.26) | 44923.08(25069.90,71159.30) | 5.36 | 159.15(67.64,311.03) | 381.73(209.53,600.52) | 3.71(3.19,4.23) |
| Kiribati | male | 140.75(33.41,377.32) | 315.62(91.41,778.91) | 1.24 | 643.56(154.00,1735.05) | 771.06(223.25,1899.94) | -0.29(-0.60,0.02) |
| Kuwait | male | 9600.95(8406.58,10851.63) | 29294.95(23332.39,36646.89) | 2.05 | 1735.91(1504.05,1966.06) | 1395.36(1121.82,1746.93) | -0.38(-0.84,0.08) |
| Kyrgyzstan | male | 15311.52(6627.01,27451.53) | 32239.14(20303.56,45389.00) | 1.11 | 1194.04(520.99,2142.75) | 1521.42(959.23,2152.78) | 0.58(0.12,1.04) |
| Lao People's Democratic Republic | male | 3655.40(976.07,8953.56) | 14885.38(7204.85,24820.97) | 3.07 | 342.38(92.01,824.49) | 630.71(309.74,1038.21) | 2.15(1.87,2.43) |
| Latvia | male | 27023.44(14218.25,41388.63) | 9089.20(6187.81,12921.22) | -0.66 | 2037.03(1070.26,3126.72) | 653.50(446.65,926.47) | -4.29(-4.99,-3.60) |
| Lebanon | male | 21283.87(15067.85,28261.53) | 36490.89(26293.35,46634.07) | 0.71 | 1797.95(1284.00,2378.10) | 1523.97(1108.86,1947.57) | -0.15(-0.50,0.20) |
| Lesotho | male | 1621.76(670.12,3199.47) | 5811.08(2949.06,9235.85) | 2.58 | 359.47(148.95,700.14) | 970.91(507.98,1519.40) | 3.97(3.61,4.33) |
| Liberia | male | 1471.13(477.33,3408.73) | 4458.30(1909.78,8443.34) | 2.03 | 244.49(80.12,564.68) | 375.21(162.15,694.28) | 2.51(2.04,2.98) |
| Libya | male | 13003.72(8277.43,18635.43) | 40535.33(28113.29,59668.22) | 2.12 | 1159.14(733.11,1678.53) | 1354.95(936.96,1959.83) | 0.23(-0.04,0.51) |
| Lithuania | male | 25827.84(12171.95,41680.05) | 10726.32(6843.17,15301.85) | -0.58 | 1453.03(685.96,2351.87) | 531.62(341.25,760.88) | -3.22(-3.87,-2.57) |
| Luxembourg | male | 1786.54(714.55,3016.93) | 564.08(372.37,784.73) | -0.68 | 792.55(318.01,1343.69) | 125.16(82.75,173.72) | -6.41(-6.66,-6.17) |
| North Macedonia | male | 21099.89(12740.12,28964.21) | 25245.28(19351.56,32422.23) | 0.20 | 2331.84(1394.13,3208.18) | 1716.18(1335.40,2176.43) | -1.48(-1.82,-1.15) |
| Madagascar | male | 5229.16(1752.06,11934.07) | 21661.55(8713.46,43536.94) | 3.14 | 178.31(59.55,402.56) | 337.73(137.65,665.61) | 2.59(2.41,2.76) |
| Malawi | male | 2381.00(664.17,6378.74) | 9203.98(3689.78,18477.47) | 2.87 | 120.99(35.15,318.32) | 250.69(102.18,503.65) | 3.06(2.77,3.35) |
| Malaysia | male | 80352.84(38872.31,120658.14) | 124788.36(86471.15,172108.09) | 0.55 | 1604.73(762.25,2434.27) | 852.17(591.64,1171.08) | -2.02(-2.15,-1.89) |
| Maldives | male | 282.44(86.45,640.76) | 703.04(453.49,1013.02) | 1.49 | 491.80(152.73,1120.73) | 331.59(212.21,480.97) | -2.21(-2.55,-1.86) |
| Mali | male | 3007.68(711.08,7995.94) | 10700.60(3519.17,22132.46) | 2.56 | 149.87(36.69,392.48) | 240.90(80.21,495.19) | 2.09(1.84,2.33) |
| Malta | male | 1563.69(653.16,2602.70) | 1014.33(743.13,1299.59) | -0.35 | 821.93(341.46,1367.81) | 259.70(190.71,329.53) | -3.80(-4.12,-3.47) |
| Marshall Islands | male | 50.21(11.02,135.94) | 194.03(67.02,397.07) | 2.86 | 503.12(109.30,1355.49) | 846.73(289.81,1724.95) | 1.73(1.46,2.01) |
| Mauritania | male | 2852.78(1095.85,5426.29) | 7196.20(4034.83,11000.04) | 1.52 | 570.18(222.99,1075.20) | 669.26(378.37,997.90) | 0.44(0.15,0.73) |
| Mauritius | male | 5391.64(2869.49,8275.34) | 4861.42(2404.83,7528.56) | -0.10 | 1402.50(746.30,2162.57) | 596.14(292.61,922.02) | -3.76(-4.22,-3.30) |
| Mexico | male | 127396.10(67893.93,194989.22) | 266776.09(190342.53,349623.04) | 1.09 | 575.71(304.91,885.61) | 468.66(336.23,614.41) | -1.03(-1.29,-0.76) |
| Republic of Moldova | male | 25563.75(12398.54,41805.00) | 21642.74(12535.37,31819.71) | -0.15 | 1435.94(693.39,2347.37) | 914.21(526.96,1344.89) | -1.43(-2.05,-0.81) |
| Mongolia | male | 6889.07(2972.47,12268.12) | 36716.91(24835.70,50972.74) | 4.33 | 1366.34(591.13,2417.88) | 2999.63(2066.78,4107.74) | 2.52(2.13,2.91) |
| Montenegro | male | 4746.53(2775.51,6837.16) | 5106.94(3871.58,6430.22) | 0.08 | 1682.19(981.22,2431.48) | 1173.33(889.91,1471.62) | -1.32(-1.56,-1.08) |
| Morocco | male | 79534.58(40715.98,124531.74) | 300091.60(215584.75,388032.30) | 2.77 | 1099.33(570.75,1704.74) | 1885.17(1365.65,2408.07) | 1.62(1.47,1.77) |
| Mozambique | male | 2617.77(638.51,7471.30) | 16056.49(6543.73,33848.92) | 5.13 | 86.39(21.58,245.34) | 289.11(118.50,611.46) | 5.51(5.08,5.94) |
| Myanmar | male | 75285.35(25089.46,167491.88) | 226923.01(133949.63,325828.68) | 2.01 | 622.41(208.57,1366.99) | 1042.71(618.35,1490.39) | 2.21(1.91,2.51) |
| Namibia | male | 1889.82(842.56,3276.38) | 5821.58(3474.64,8639.95) | 2.08 | 553.00(245.30,953.21) | 920.06(553.92,1349.64) | 1.78(1.40,2.16) |
| Nepal | male | 16805.66(4554.43,40481.02) | 114252.41(67290.95,166837.52) | 5.80 | 320.65(88.69,761.62) | 1045.68(617.46,1523.98) | 4.75(4.42,5.08) |
| Netherlands | male | 69210.81(33247.93,109621.39) | 21705.96(16021.55,27585.90) | -0.69 | 815.62(392.00,1296.18) | 147.91(109.53,186.54) | -6.33(-6.58,-6.09) |
| New Zealand | male | 4155.74(249.46,10472.46) | 1954.70(344.82,3965.58) | -0.53 | 235.94(14.05,599.61) | 58.83(10.44,117.65) | -5.49(-5.78,-5.19) |
| Nicaragua | male | 1384.85(459.17,3007.67) | 8026.31(4280.39,12958.51) | 4.80 | 189.06(62.22,406.80) | 405.09(217.69,636.57) | 2.56(2.29,2.83) |
| Niger | male | 3014.64(640.13,8810.93) | 10114.13(2601.69,25148.32) | 2.36 | 193.70(42.11,552.73) | 252.45(67.63,627.26) | 1.08(0.89,1.27) |
| Nigeria | male | 85790.16(34125.27,192687.62) | 289442.25(165207.36,448609.61) | 2.37 | 363.09(149.09,795.28) | 662.62(386.32,1020.10) | 2.14(1.90,2.37) |
| Democratic People's Republic of Korea | male | 53360.06(19975.05,99180.85) | 206660.47(122677.57,296148.57) | 2.87 | 771.08(286.16,1414.05) | 1461.01(879.00,2051.25) | 2.50(2.37,2.62) |
| Northern Mariana Islands | male | 98.97(29.70,241.57) | 138.10(70.06,223.46) | 0.40 | 568.23(168.19,1360.83) | 453.61(230.49,732.34) | -0.58(-1.14,-0.03) |
| Norway | male | 15189.86(3965.57,28968.48) | 2307.89(850.98,4027.97) | -0.85 | 543.31(142.55,1030.65) | 54.56(20.31,95.69) | -8.08(-8.36,-7.81) |
| Oman | male | 9805.82(5375.95,15438.13) | 22181.85(16410.86,28775.27) | 1.26 | 2263.55(1251.29,3548.18) | 1954.72(1490.40,2492.40) | -0.82(-1.40,-0.24) |
| Pakistan | male | 146042.47(48429.49,304888.86) | 1051194.62(671847.02,1535066.49) | 6.20 | 444.06(148.04,927.60) | 1569.19(1009.91,2276.68) | 4.75(4.37,5.14) |
| Palestine | male | 5587.61(2798.89,8792.05) | 18677.45(13911.41,23557.35) | 2.34 | 1379.97(692.21,2162.81) | 1515.40(1127.45,1900.69) | -0.27(-0.74,0.20) |
| Panama | male | 3008.86(1394.16,5400.71) | 4964.58(2875.04,7459.61) | 0.65 | 383.96(177.29,693.01) | 244.34(141.11,366.38) | -1.03(-1.30,-0.76) |
| Papua New Guinea | male | 2667.49(474.77,8482.04) | 12054.15(2727.08,30289.73) | 3.52 | 232.87(42.03,739.86) | 384.22(88.34,977.77) | 1.77(1.59,1.95) |
| Paraguay | male | 3657.59(1546.66,6704.25) | 10099.80(6216.45,15674.54) | 1.76 | 324.38(137.48,595.23) | 358.90(222.49,556.18) | 0.18(-0.05,0.42) |
| Peru | male | 37367.73(17503.98,61207.83) | 56129.88(37758.86,81103.13) | 0.50 | 591.64(276.13,973.12) | 352.20(236.12,509.99) | -1.53(-2.01,-1.05) |
| Philippines | male | 66683.36(32998.47,114777.10) | 411917.23(275775.87,577391.57) | 5.18 | 418.76(207.37,725.44) | 952.88(645.18,1325.37) | 3.28(2.34,4.23) |
| Poland | male | 425751.69(248461.95,600660.92) | 217978.72(166885.58,287182.09) | -0.49 | 2294.21(1334.17,3237.17) | 768.84(586.70,1007.28) | -4.16(-4.34,-3.98) |
| Portugal | male | 33119.28(10341.36,61477.96) | 10607.40(6138.12,15391.32) | -0.68 | 566.48(177.80,1054.14) | 114.10(66.29,166.21) | -6.05(-6.42,-5.69) |
| Puerto Rico | male | 3630.34(21.68,11953.52) | 2233.71(459.23,4173.72) | -0.38 | 217.66(1.30,718.05) | 81.71(17.01,153.94) | -4.13(-4.72,-3.54) |
| Qatar | male | 2935.38(2311.33,3733.72) | 10427.60(7760.54,13839.72) | 2.55 | 3023.77(2407.17,3715.54) | 1207.95(927.00,1541.29) | -3.50(-3.78,-3.22) |
| Romania | male | 179595.18(93190.48,275348.46) | 127424.97(97844.61,164268.10) | -0.29 | 1454.32(757.11,2213.11) | 844.58(650.88,1088.49) | -2.70(-3.13,-2.26) |
| Russian Federation | male | 1219397.12(541565.69,1974489.91) | 775138.53(436699.55,1146986.72) | -0.36 | 1861.16(804.12,3043.36) | 863.39(482.15,1266.74) | -2.96(-3.74,-2.17) |
| Rwanda | male | 5206.88(1470.68,12153.09) | 11086.34(4460.08,21565.92) | 1.13 | 365.68(104.14,852.84) | 395.48(161.21,767.69) | -0.49(-0.93,-0.06) |
| Saint Lucia | male | 306.87(109.89,615.96) | 593.13(268.41,981.21) | 0.93 | 786.11(279.02,1579.58) | 572.13(258.58,948.47) | -1.62(-1.94,-1.31) |
| Saint Vincent and the Grenadines | male | 225.91(66.92,473.48) | 486.08(195.90,832.85) | 1.15 | 689.09(204.06,1451.60) | 701.21(282.29,1203.86) | -0.42(-0.70,-0.15) |
| Samoa | male | 289.59(70.37,748.21) | 494.32(140.58,1099.31) | 0.71 | 618.84(150.86,1601.52) | 615.42(177.97,1355.10) | -0.27(-0.39,-0.15) |
| Sao Tome and Principe | male | 66.72(24.44,141.26) | 387.59(197.15,636.08) | 4.81 | 220.06(82.08,463.58) | 695.07(363.50,1125.18) | 4.59(4.30,4.87) |
| Saudi Arabia | male | 56400.79(30542.49,88678.98) | 328274.85(250426.33,421494.66) | 4.82 | 1431.26(788.22,2243.39) | 2238.31(1785.22,2744.01) | 1.32(0.73,1.91) |
| Senegal | male | 5835.27(1949.70,13021.56) | 20299.46(9668.10,35639.41) | 2.48 | 342.10(114.33,755.24) | 529.90(252.61,921.31) | 1.35(1.15,1.55) |
| Serbia | male | 97088.93(56323.11,139237.98) | 79869.33(60993.45,101862.92) | -0.18 | 1840.38(1054.52,2633.08) | 1160.09(891.27,1479.22) | -1.93(-2.34,-1.51) |
| Seychelles | male | 269.74(148.13,420.33) | 401.08(212.43,604.35) | 0.49 | 1038.81(569.68,1627.72) | 672.40(358.72,1015.03) | -2.16(-2.40,-1.91) |
| Sierra Leone | male | 2623.26(792.83,6417.56) | 7851.43(2990.02,16141.63) | 1.99 | 260.04(79.57,631.71) | 395.25(154.19,797.87) | 1.93(1.63,2.22) |
| Singapore | male | 11188.64(3942.17,19271.84) | 11291.87(7272.96,15124.89) | 0.01 | 995.36(346.28,1727.52) | 281.70(182.79,378.66) | -4.50(-4.83,-4.18) |
| Slovakia | male | 61862.63(33889.57,90128.49) | 30734.21(22177.02,40427.17) | -0.50 | 2403.78(1311.44,3510.58) | 797.73(584.71,1042.14) | -3.88(-4.11,-3.65) |
| Slovenia | male | 11694.73(6219.11,18656.19) | 5537.26(4036.19,7357.59) | -0.53 | 1171.76(620.90,1863.48) | 307.54(224.04,407.85) | -5.27(-5.51,-5.03) |
| Solomon Islands | male | 308.21(53.28,1001.11) | 1659.28(432.54,4379.24) | 4.38 | 338.86(59.37,1092.45) | 813.93(209.89,2136.34) | 2.92(2.79,3.04) |
| Somalia | male | 1371.73(265.86,4345.76) | 3983.61(856.74,12128.33) | 1.90 | 98.20(20.06,306.82) | 115.81(25.45,346.51) | 0.98(0.72,1.25) |
| South Africa | male | 82095.81(62641.79,103032.79) | 156595.90(126161.16,192255.49) | 0.91 | 775.84(588.39,977.30) | 752.95(607.67,923.08) | -0.15(-0.63,0.34) |
| Republic of Korea | male | 222520.24(123041.61,325866.38) | 139797.95(113154.83,175481.68) | -0.37 | 1587.82(870.76,2364.25) | 351.50(283.09,437.88) | -5.40(-5.75,-5.06) |
| South Sudan | male | 3108.54(995.75,7000.08) | 6408.84(2504.57,12113.57) | 1.06 | 220.78(72.14,487.65) | 300.04(118.95,566.69) | 1.37(1.20,1.54) |
| Spain | male | 103755.62(40560.00,179807.54) | 45228.39(30704.52,61064.52) | -0.56 | 458.67(179.06,798.23) | 117.06(79.43,157.97) | -4.77(-5.08,-4.46) |
| Sri Lanka | male | 38194.61(16090.85,69027.54) | 68080.19(43196.43,99588.69) | 0.78 | 654.73(276.29,1185.40) | 592.56(379.74,867.50) | -0.13(-0.66,0.41) |
| Sudan | male | 34611.75(11671.73,77223.16) | 201135.32(118150.81,319788.39) | 4.81 | 666.88(227.19,1466.18) | 1821.06(1072.46,2852.70) | 3.74(3.64,3.84) |
| Suriname | male | 1453.19(529.27,2605.52) | 2749.05(1302.61,4603.90) | 0.89 | 1090.10(398.10,1943.57) | 931.19(443.66,1561.41) | -0.77(-1.16,-0.38) |
| Eswatini | male | 629.45(282.33,1102.46) | 2527.74(1395.10,3874.08) | 3.02 | 450.04(203.36,787.68) | 931.70(526.88,1413.16) | 2.75(2.27,3.22) |
| Sweden | male | 23971.90(4172.87,49749.92) | 4152.06(1095.22,8234.16) | -0.83 | 378.85(65.88,783.87) | 45.74(12.01,90.82) | -7.29(-7.64,-6.94) |
| Switzerland | male | 26399.80(11669.71,43193.98) | 7555.97(5013.68,10137.69) | -0.71 | 615.00(272.26,1007.75) | 100.67(67.20,134.70) | -6.24(-6.43,-6.06) |
| Syrian Arab Republic | male | 83379.15(59745.24,110650.58) | 143190.66(100729.47,197787.75) | 0.72 | 2656.46(1902.07,3523.06) | 2167.78(1549.79,2957.39) | -1.05(-1.23,-0.86) |
| Taiwan (Province of China) | male | 66447.18(36644.17,103077.74) | 79025.54(60776.77,103040.53) | 0.19 | 753.54(413.88,1171.41) | 443.51(341.19,576.48) | -1.84(-1.98,-1.69) |
| Tajikistan | male | 12097.57(4125.02,23882.39) | 54525.68(29206.06,83382.36) | 3.51 | 944.09(321.82,1863.55) | 2296.99(1217.58,3540.01) | 3.28(3.03,3.53) |
| United Republic of Tanzania | male | 7928.14(2707.48,18108.47) | 37777.87(16944.18,70835.67) | 3.77 | 140.74(49.08,319.06) | 303.93(138.39,562.56) | 2.81(2.67,2.95) |
| Thailand | male | 152026.42(73417.24,243789.55) | 308654.55(224966.86,423465.36) | 1.03 | 815.41(391.27,1298.95) | 666.54(487.18,908.13) | -1.35(-1.65,-1.05) |
| Bahamas | male | 628.32(167.40,1193.75) | 1078.30(309.32,2028.10) | 0.72 | 820.83(215.37,1574.91) | 552.94(157.40,1047.99) | -1.65(-1.83,-1.47) |
| Gambia | male | 539.11(153.71,1320.00) | 3223.21(1541.05,5571.82) | 4.98 | 267.27(78.62,653.33) | 641.11(309.71,1094.53) | 3.11(2.95,3.28) |
| Timor-Leste | male | 192.97(44.93,548.99) | 2031.62(837.61,3760.45) | 9.53 | 116.03(27.55,323.80) | 485.09(200.72,887.81) | 6.40(5.69,7.12) |
| Togo | male | 2196.84(783.30,4799.79) | 12295.29(5611.88,21888.38) | 4.60 | 340.40(123.54,731.26) | 670.28(312.20,1167.44) | 2.37(2.10,2.63) |
| Tonga | male | 92.26(24.92,235.37) | 182.41(58.64,393.31) | 0.98 | 324.41(86.97,822.68) | 473.13(152.72,1013.86) | 1.12(0.81,1.44) |
| Trinidad and Tobago | male | 5765.17(1257.18,11258.01) | 7095.88(2410.30,12866.93) | 0.23 | 1368.10(292.85,2686.44) | 789.33(270.13,1424.29) | -2.51(-2.83,-2.19) |
| Tunisia | male | 35034.54(23132.02,47984.51) | 78463.87(52038.28,110625.53) | 1.24 | 1316.56(864.96,1799.19) | 1267.28(843.08,1786.89) | -0.44(-0.76,-0.11) |
| Turkey | male | 276779.80(205733.71,343504.07) | 341304.49(259273.83,444680.84) | 0.23 | 1444.15(1070.76,1784.35) | 792.59(604.40,1025.65) | -2.32(-2.52,-2.11) |
| Turkmenistan | male | 20991.92(8820.88,34431.92) | 50519.70(30776.24,74381.42) | 1.41 | 2402.32(1015.02,3977.98) | 2604.04(1579.50,3819.07) | -0.38(-0.81,0.04) |
| Uganda | male | 4686.69(1340.60,11642.90) | 26877.43(12137.25,49257.86) | 4.73 | 140.19(40.33,348.14) | 381.26(176.04,684.26) | 3.82(3.53,4.11) |
| Ukraine | male | 469414.07(232246.26,733857.69) | 478834.84(280024.79,702361.95) | 0.02 | 1825.86(893.65,2878.09) | 1690.55(1000.57,2482.83) | -0.75(-1.23,-0.26) |
| United Arab Emirates | male | 11495.66(8309.42,16452.52) | 82863.31(55740.35,122884.21) | 6.21 | 2604.45(1923.13,3549.70) | 1581.72(1091.88,2208.83) | -2.17(-2.46,-1.88) |
| United Kingdom | male | 330183.78(138209.43,543236.97) | 82854.23(55002.22,112585.97) | -0.75 | 891.00(376.69,1461.90) | 158.27(105.04,213.85) | -6.43(-6.63,-6.22) |
| United States of America | male | 791818.31(306915.19,1398599.66) | 338207.99(180645.76,510873.59) | -0.57 | 593.47(230.22,1054.43) | 143.33(77.07,215.46) | -5.29(-5.53,-5.06) |
| Uruguay | male | 8146.10(2543.91,16606.87) | 4459.11(2181.97,7017.36) | -0.45 | 486.78(153.09,987.65) | 207.44(101.60,326.11) | -3.21(-3.43,-2.98) |
| Uzbekistan | male | 89247.59(38399.20,147182.43) | 362773.49(232675.01,504247.08) | 3.06 | 1775.38(753.37,2937.01) | 3774.02(2399.34,5246.42) | 2.36(1.76,2.96) |
| Vanuatu | male | 183.49(38.87,500.59) | 833.77(236.65,1917.80) | 3.54 | 429.53(91.96,1158.53) | 804.33(227.82,1863.70) | 1.77(1.57,1.98) |
| Venezuela (Bolivarian Republic of) | male | 50468.07(22893.89,81834.66) | 123900.37(79211.79,182393.81) | 1.46 | 977.16(444.03,1593.54) | 870.11(558.11,1282.14) | -0.59(-0.85,-0.32) |
| Viet nam | male | 91334.45(33284.85,190684.20) | 463948.19(317783.51,624809.11) | 4.08 | 517.84(189.31,1070.27) | 1075.72(749.09,1423.35) | 3.09(2.55,3.64) |
| Virginia | male | 19746.66(8406.79,33915.45) | 6425.66(2275.66,11285.41) | -0.67 | 643.67(273.88,1107.09) | 105.07(36.92,183.74) | -6.51(-6.89,-6.13) |
| Yemen | male | 17344.74(4671.53,44206.42) | 133837.80(66722.53,217270.97) | 6.72 | 645.11(177.36,1623.93) | 1782.23(894.00,2865.99) | 3.92(3.67,4.16) |
| Zambia | male | 3704.72(1314.87,7782.57) | 24687.65(12264.38,40967.75) | 5.66 | 231.91(83.67,488.64) | 672.46(337.53,1099.65) | 3.86(3.73,4.00) |
| Zimbabwe | male | 5769.24(2470.88,10627.56) | 14675.02(6939.15,25204.48) | 1.54 | 282.02(121.64,512.50) | 456.76(221.01,772.91) | 1.29(0.71,1.88) |
| Monaco | male | 88.85(8.82,189.38) | 71.26(41.32,104.58) | -0.20 | 325.29(32.35,697.36) | 187.75(107.85,272.50) | -1.55(-2.18,-0.92) |
| San Marino | male | 35.33(8.02,72.09) | 30.82(12.35,55.44) | -0.13 | 241.50(54.93,489.24) | 113.14(45.37,205.56) | -3.05(-3.56,-2.55) |
| Saint Kitts and Nevis | male | 104.55(35.34,207.87) | 109.84(47.80,187.37) | 0.05 | 669.19(225.54,1318.15) | 318.05(138.80,535.73) | -3.62(-4.17,-3.07) |
| Cook Islands | male | 23.40(8.49,56.27) | 23.99(4.32,56.84) | 0.03 | 318.07(115.27,770.53) | 214.25(38.61,505.18) | -1.39(-1.75,-1.04) |
| Nauru | male | 23.05(6.54,64.52) | 20.62(4.84,48.15) | -0.11 | 763.01(215.88,2164.65) | 637.61(147.86,1496.70) | -1.21(-1.49,-0.92) |
| Niue | male | 5.01(1.96,11.36) | 4.23(0.76,9.80) | -0.16 | 526.85(205.91,1193.45) | 420.55(76.09,970.22) | -1.40(-1.68,-1.11) |
| Palau | male | 31.89(0.27,97.59) | 59.33(0.20,148.90) | 0.86 | 547.51(4.73,1678.49) | 462.51(1.54,1158.57) | 0.31(-0.53,1.15) |
| Tokelau | male | 2.18(0.05,8.14) | 1.65(0.01,4.54) | -0.24 | 372.41(9.30,1394.44) | 246.68(0.98,670.71) | -1.84(-2.15,-1.53) |
| Tuvalu | male | 11.95(2.97,34.87) | 25.40(9.11,55.15) | 1.13 | 363.77(91.06,1051.69) | 472.90(170.64,1029.89) | 0.59(0.37,0.80) |
